# A recyclable type‐I photosensitizer to enable red‐light‐driven gram‐scale aerobic photocatalysis

**DOI:** 10.1002/smo.20250003

**Published:** 2025-06-04

**Authors:** Shirong Yan, Lu Qiao, Lei Chen, Wu‐Jie Guo, Hui‐Qing Peng

**Affiliations:** ^1^ Beijing Advanced Innovation Center for Soft Matter Science and Engineering Beijing University of Chemical Technology Beijing China

**Keywords:** organic photosensitizer, purely organic photocatalyst, reactive oxygen species, superoxide radical, type‐I photosensitizer

## Abstract

Developing metal‐free, purely organic photocatalysts with high recyclability and the ability to utilize red light to yield specific reactive oxygen species for aerobic photocatalysis is both crucial and challenging in current research. Herein, we first found that a type‐I photosensitizer, **EtNBS‐H**, can achieve red‐light‐driven aerobic photocatalysis with remarkable catalytic performance and facile recoverability. Upon irradiation with red light, **EtNBS‐H** exclusively generates O_2_^−•^, enabling the efficient hydroxylation of arylboronic acids, and oxidization of thioethers and other substrates with conversion exceeding 99%. Significantly, **EtNBS‐H** stands out for its simple recovery and reuse through a facile pH‐tunable acid‐base reaction. This allows for the attainment of high‐purity products through extraction, and enables the retrieval of the photocatalyst from the reaction medium for subsequent reuse with an average recovery rate exceeding 94%. Moreover, utilizing **EtNBS‐H** as a photocatalyst in the scale‐up reaction, the gram‐scale products with a yield of >95% and purity of >99% were obtained, highlighting its potential for the guidance of developing recyclable organic photocatalysts that harness red light. This work offers a promising approach for sustainable and large‐scale photocatalytic organic synthesis.

## Graphical abstract

We report a recyclable type‐I photosensitizer, **EtNBS‐H**, which enables efficient red‐light‐driven photooxidation with conversion exceeding 99%. Its strong absorption of red light enhances its scalability for industrial applications. **EtNBS‐H** can be easily recovered and reused through a simple pH‐tunable acid‐base reaction, achieving a recovery rate of over 94%, making it a promising candidate for sustainable green synthesis.

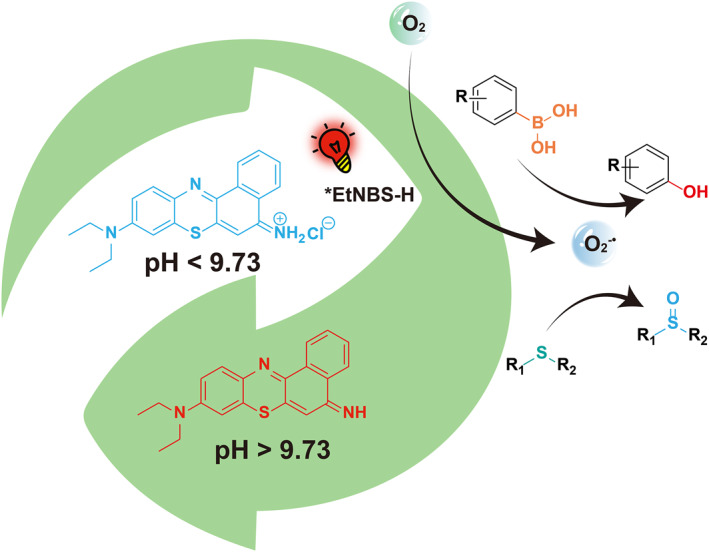

## 1 INTRODUCTION

Aerobic photocatalysis, which enables intricate organic transformations in the presence of light, molecular oxygen (O_2_), and a photocatalyst, has attracted significant attention for its potential to align with the principles of sustainable chemistry and minimize waste generation.^[1]^ Among the diverse range of reported photocatalysts, organic photocatalysts are distinguished by their advantages, including well‐defined molecular structures, extensive photophysical and photochemical properties, and the significant potential for structural modifications.^[2]^ Particularly from a green chemistry perspective, the purely organic photocatalysts (photosensitizers with catalytic capability) are regarded as eco‐friendly and sustainable alternatives due to the absence of metal components.^[3]^ Upon exposure to light, the excited photocatalysts can activate O_2_ to yield reactive oxygen species (ROS) via two distinct mechanisms: electron transfer (type‐I pathway) and energy transfer (type‐II pathway). The type‐I pathway leads to the generation of oxygen radicals, such as hydroxyl radical (•OH) and superoxide radical (O_2_^−•^), whereas the type‐II pathway yields singlet oxygen (^1^O_2_).^[4]^ These ROS are capable of efficiently transforming a wide range of substrates into desired products. Particularly, different ROS exhibit distinct catalytic scenarios and performances, such as reaction type, conversion, and selectivity, which presents substantial opportunities in achieving precise control over photocatalytic processes.^[5]^ Among these ROS, O_2_^−•^ possesses redox property, free radical nature, nucleophilicity, and basicity, which demonstrates a great potential in organic redox synthesis.^[6]^ Generating O_2_^−•^ exclusively using a photocatalyst is critical for enhancing the selectivity of target products, reducing the yield of byproducts, and understanding the photocatalytic reaction process and mechanism. However, the number of reported organic photocatalysts capable of exclusively producing O_2_^−•^ remains limited. This might be because organic photocatalysts often accompany the type‐II pathway while generating O_2_^−•^ through the type‐I pathway.^[7]^ Hence, it is both highly significant and challenging to seek and develop organic photocatalysts that can individually generate O_2_^−•^.

To date, photocatalyst‐mediated aerobic photocatalysis is predominantly activated by short‐wavelength light sources (<620 nm) due to the requirement of high‐energy photon irradiation for exciting photocatalysts to their desired excited states.^[8]^ However, the photons in the ultraviolet and short visible regions are more readily absorbed by the substrate or product. This not only reduces the excitation of photocatalysts but also leads to the generation of byproducts.^[9]^ Additionally, the limited penetration depth of short‐wavelength light into reaction media hinders the reaction rate and conversion, presenting substantial obstacles for large‐scale applications. To mitigate these issues, a viable approach is to develop photocatalysts capable of utilizing red and near infrared (NIR) light (>620 nm),^[10]^ which is still an extremely challenging undertaking. Another critical aspect in photocatalysis is the recyclability of the photocatalyst, which directly impacts the sustainability, cost‐effectiveness, and greenness of the process.^[11]^ A recyclable and robust photocatalyst minimizes resource consumption, lowers production costs, and streamlines product purification processes, all of which are essential for the large‐scale application of photocatalytic scenarios. While homogeneous organic photocatalysts offer advantages in terms of efficiency and tunability, they usually lack the recyclability characteristic of heterogeneous inorganic photocatalysts.^[12]^ Building upon the aforementioned points, the pursuit of developing a long‐wavelength light‐absorbing organic photocatalyst that can efficiently generate O_2_^−•^ in isolation, coupled with exceptional recyclability, is a formidable yet indispensable challenge.

**FIGURE 1:**
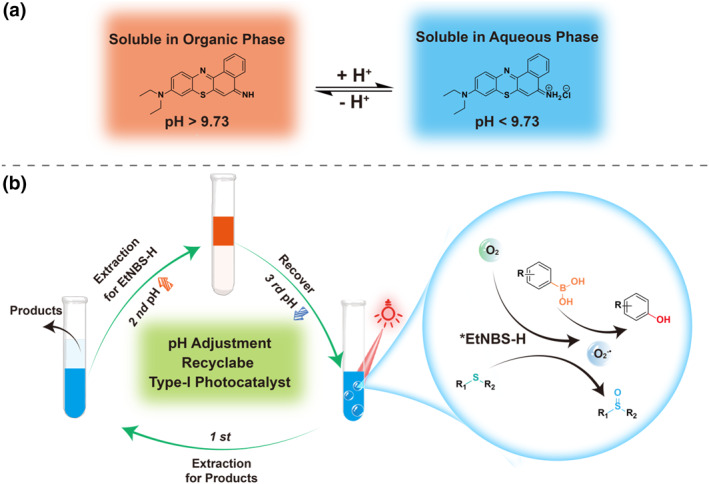
Schematic illustration. (a) The chemical structure of **EtNBS‐H** (in blue) and its reversible acid‐base reaction. (b) The recyclable type‐I photocatalyst **EtNBS‐H** for red‐light‐driven photooxidation. The immiscible solvents are diethyl ether and water, respectively.

With these in mind, we herein selected **EtNBS‐H**, the core structure of Nile blue derivative, as the target to investigate its photophysical properties and photocatalytic performance. The rationales are as follows: First, it demonstrates absorption of red light (*λ*_abs_ = 650 nm) and possesses water solubility due to the cationization; Second, **EtNBS‐H** has a high p*K*_a_, ensuring its structural stability during photocatalytic reactions and enabling a broader range of applicable substrates; Third, our theoretical calculations demonstrate that **EtNBS‐H** exhibits a high intersystem crossing (ISC) efficiency with Δ*E*_ST_ = 0.2278 eV. Moreover, the planar structure of **EtNBS‐H** promotes favorable O_2_ adsorption, while the overlap between its molecular orbitals in the excited triplet state and O_2_ facilitates through‐space charge transfer. These factors significantly contribute to the generation of O_2_^−•^. Experimentally, **EtNBS‐H** was successfully synthesized and was demonstrated to serve as a green and multifunctional photocatalyst applicable in various aerobic photocatalysis in both water and organic solvents (i.e., photooxidation of arylboronic acids, thioethers, and other substrates) with substrate conversion as high as > 99%. Adjusting the pH of the photocatalytic reaction system can render **EtNBS‐H** hydrophobic due to the presence of an iminium group at the 5‐position of **EtNBS‐H** that facilitates reversible acid‐base reactions (Figure 1a and Figure S1). This allows for the separation of **EtNBS‐H** from the product through extraction (as illustrated in Figure 1b), resulting in the attainment of high‐purity products (>99%), and enabling the retrieval of the photocatalyst from the reaction medium for subsequent reuse (with an average recovery rate exceeding 94%). Notably, **EtNBS‐H** has shown potential for scalability in gram scale photocatalytic reactions, underscoring its viability for green synthesis and commercial application. To the best of our knowledge, this is the first study to utilize a homogeneous type‐I photocatalyst for red‐light‐driven photooxidation, demonstrating its high catalytic efficiency, outstanding recyclability and stability, aligning with green synthesis principles.

## 2 RESULTS AND DISCUSSION

### 2.1 Synthesis and photophysical properties

**FIGURE 2:**
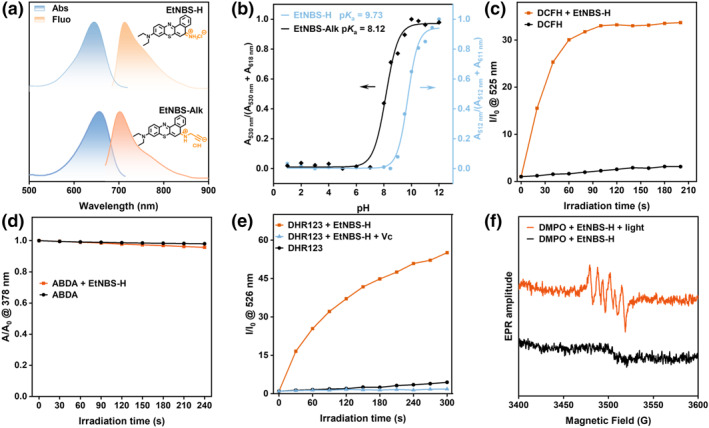
Photophysical properties and reactive oxygen species (ROS) generation capacity of **EtNBS‐H**. (a) Absorbance and PL spectra of **EtNBS‐H** and **EtNBS‐Alk** in MeOH. (b) The pH‐dependent absorption ratio of **EtNBS‐H** and **EtNBS‐Alk** in PBS buffer solutions. (c) ROS generation of **EtNBS‐H** (5 μM) using DCFH (1 μM) as an indicator. (d) Absorbance decay of ABDA (100 μM) in the absence and presence of **EtNBS‐H** (5 μM). (e) O_2_^−•^ generation and quenching of **EtNBS‐H** (5 μM) using DHR123 (10 μM) as an indicator and Vc as the O_2_^−•^ quencher. (f) EPR spectra to detect O_2_^−•^ production from **EtNBS‐H** under irradiation, using DMPO as the spin‐trap agent.

**EtNBS‐H** was synthesized from the starting materials of *N,N*‐diethyl‐p‐phenylenediamine and 1‐naphthylamine. The synthetic details are provided in the Supporting Information (Figure S2) and their chemical structures were characterized by ^1^H NMR, ^13^C NMR, and high‐resolution mass spectrometry (Figures S3–S11). As shown in Figure 2a, the **EtNBS‐H** solution displays a strong absorption band in the red‐light region with a peak at 650 nm (*ε* = 5.8 × 10^4^ M^−1^ cm^−1^) and gives an intense emission peak at 714 nm upon light irradiation. Moreover, in spite of its purely organic structure, the cationization of **EtNBS‐H** renders it readily water soluble, which makes it suitable for green aqueous phase photocatalysis. Considering the feasibility and convenience of recovering the homogeneous organic photocatalyst by utilizing acid‐base reaction and subsequent liquid‐liquid extraction, we investigated the acid dissociation constant (p*K*_a_) of **EtNBS‐H** (Figure 2b and Figure S12). It is determined that the p*K*_a_ value of **EtNBS‐H** is 9.73. This characteristic provides an opportunity to recover **EtNBS‐H** from the mixed reaction solution.

Besides, in photocatalysis, tertiary aliphatic amines, which are organic bases, are widely employed as sacrificial electron donors to reduce the oxidized photocatalyst. As a result, the high p*K*_a_ value of **EtNBS‐H** suggests a strong likelihood of surpassing those of the sacrificial donors, thereby ensuring its robust structural stability in photocatalytic systems. Further studies found 5‐position functional groups (highlighted in orange on the structure) play a pivotal role in determining the acidity strengths of the hydrochloride salts. When using a propargyl group to replace the hydrogen atom, the obtained **EtNBS‐Alk** shows negligible differences in absorption and emission characteristics (*λ*_abs_ = 655 nm and *λ*_em_ = 703 nm) when compared to **EtNBS‐H**. However, the p*K*_a_ value of **EtNBS‐Alk** significantly changes to be 8.12, which is unfavorable for its stability during photocatalytic processes. The validity of this point was corroborated through photocatalytic experiments, which revealed that **EtNBS‐Alk** exhibits diminished stability in a catalytic environment containing triethanolamine (TEOA), leading to a substantial decline in photocatalytic efficiency (Figure S13). Therefore, future research on such photocatalysts should place particular emphasis on the controlling of the 5‐position functional group.

### 2.2 Investigation of ROS generation

The ROS generation capacity of **EtNBS‐H** was assessed using 2′,7′‐dichlorodihydrofluorescein (DCFH) as a fluorescent probe. Upon red light irradiation, the fluorescence intensity of DCFH exhibits a rapid enhancement in the presence of **EtNBS‐H**, while no significant increase in fluorescence signal is observed for the solution containing DCFH alone (Figure 2c and Figure S14). This finding suggests that **EtNBS‐H** can be an efficient photocatalyst for the generation of ROS. To confirm the specific forms of ROS, 9,10‐anthracenediyl‐bis(methylene)‐dimalonic acid (ABDA) was employed to detect ^1^O_2_ (Figure 2d and Figure S15). It is observed that the presence of **EtNBS‐H** does not lead to obvious attenuation in ABDA absorbance upon light irradiation, indicating that no ^1^O_2_ was detected. Furthermore, we have studied the oxygen radical production from the photocatalyst via dihydrorhodamine 123 (DHR123). Significant increase in the fluorescence intensity of DHR123 is observed upon exposure to illumination when its solution is treated with **EtNBS‐H**. The radical quencher vitamin C (Vc) was introduced to verify the contribution of oxygen radicals to the observed fluorescence intensity enhancement of DHR123. As shown in Figure 2e and Figure S16, the emission of DHR123 is remarkably weakened upon the addition of Vc to quench radicals, providing convincing evidence that **EtNBS‐H** effectively produces free radical ROS through the type‐I pathway. To further elucidate the specific type of ROS, an electron paramagnetic resonance investigation was conducted using 5,5‐dimethyl‐1‐pyrroline‐N‐oxide (DMPO) as the spin trap for capturing free radicals (Figure 2f). Upon red light irradiation of the **EtNBS‐H** and DMPO mixture, a characteristic paramagnetic adduct is observed, which correlates with the O_2_^−•^ signal, thereby demonstrating the generation of O_2_^−•^. The aforementioned results comprehensively demonstrate that **EtNBS‐H** is an efficient red‐light‐absorbing photocatalyst with unique ability to generate O_2_^−•^, positioning it as a promising candidate in aerobic photocatalysis for organic redox synthesis.

### 2.3 Theoretical calculations

**FIGURE 3:**
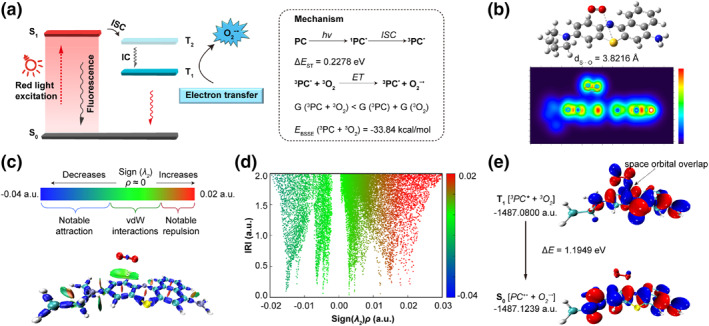
Theoretical calculations. (a) Schematic illustration of O_2_^−•^ generation via electron transfer process. (b) Lowest energy configuration of [**EtNBS‐H**…O_2_] with ELF analysis (XZ view). (c) Interaction distribution regions and (d) scatter plots showing intra/intermolecular interactions within [**EtNBS‐H**…O_2_], as depicted by IRI. The IRI isosurfaces, colored by the sign (*λ*_2_)*p* function, illustrate the interaction nature: red to green to blue indicates repulsion, significant interaction, and attraction, respectively (vdW for van der Waals). (e) Molecular orbital and energy changes pre‐ and post‐electron transfer.

Comprehensive understanding of the photophysical and photochemical behaviors underlying the type‐I ROS generator, **EtNBS‐H**, is essential for gaining profound insights into its photocatalytic performance. Therefore, we have systematically studied these mechanisms by theoretical technologies including Gaussian 09, MOPAC, molclus, and Multiwfn programs.^[13]^ Ground states and excited states of **EtNBS‐H** were evaluated through density functional theory (DFT) and time‐dependent DFT (TD‐DFT) calculations at the M06‐2X/6‐311+G* level. As shown in Figure 3a and Table S1, it is found that the singlet (S_1_) and triplet (T_2_) excited states of **EtNBS‐H** have a small Δ*E*_ST_ value of 0.2278 eV, which is conducive to a high ISC rate.^[14]^ Notably, the calculated lowest triplet state (T_1_) energy of 1.1339 eV is lower than the 1.63 eV energy of O_2_ (^1^Ʃg^+^), effectively preventing the way from transition O_2_ (^1^Ʃg^+^) to O_2_ (^1^Δg), and thus reducing the likelihood of ^1^O_2_ generation. The efficiency of O_2_^−•^ production is critically dependent on the spontaneous electron transfer from the excited state of **EtNBS‐H** derivatives to molecular oxygen.^[15]^ Therefore, considering the importance of close proximity to efficient electron transfer, a conformation search for the [**EtNBS‐H**…O_2_] dimer was conducted, and the lowest energy configuration was selected for further mechanism exploration. The observed decrease in Gibbs‐free energy suggests that spontaneous association occurs in solution, while a binding energy of −33.84 kcal/mol indicates that the dimer is more stable compared to its dispersed form. Structural optimization and electron localization function (ELF) analysis reveal that the sulfur atom in **EtNBS‐H** attracts the lone pair electrons of the O_2_ (Figure 3b). In the XZ view of the ELF map, it is evident that **EtNBS‐H** remains in a planar conformation, which has been proven to be more favorable for superoxide radical generation.^[16]^ Furthermore, the interaction region indicator (IRI) was employed to reveal the interaction between **EtNBS‐H** and O_2_, simultaneously showing covalent and noncovalent interactions in a single map. As shown in the isosurface map (IRI = 1.1), a broad green isosurface with a sign(*λ*_2_)*ρ* value < −0.005 is observed in the middle of [**EtNBS‐H**…O_2_], indicating a van der Waals (vdW) attraction (Figure 3c,d). Interestingly, when the excited state of [**EtNBS‐H**…O_2_] is investigated, it is found that the molecular orbital of [^3^ EtNBS‐H* + ^3^O_2_] is formed through space orbital overlap, indicating the presence of through‐space charge transfer. The energy difference between the electron‐exchanged ground state [**EtNBS‐H** ^+•^ + O_2_^−•^] and [^3^ EtNBS‐H* + ^3^O_2_] was calculated to be 1.1949 eV, ensuring a high probability of electron transfer from **EtNBS‐H** to oxygen molecules. Taken together, the reasons why **EtNBS‐H** facilitates the production of O_2_^−•^ can be concluded as follows: (i) the small energy gap between ^1^ EtNBS‐H* and ^3^ EtNBS‐H* facilitates efficient ISC, resulting in the abundant ^3^photocatalyst*, which provides a significant opportunity for producing ROS through interaction with O_2_. (ii) The planar structure of **EtNBS‐H** in its triplet state, attained upon red light excitation, enhances oxygen adsorption. (iii) the abundant ^3^photocatalyst* and favorable oxygen adsorption promote through‐space charge transfer, leading to efficient O_2_^−•^ generation. (iv) The T_1_ energy level of 1.13 eV is lower than the 1.63 eV required for the high‐efficiency transition from excited triplet electrons to O_2_ (^1^Ʃg^+^), thereby significantly reducing the likelihood of a type‐II pathway.

### 2.4 Aerobic oxidative hydroxylation of arylboronic acids

Phenolic compounds and sulfoxides are integral to the fields of pesticides, pharmaceuticals, and material science. However, traditional synthetic methods frequently necessitate the use of strong oxidants and harsh reaction conditions, which limits their sustainability. Aerobic hydroxylation of phenylboronic acids and oxidation of sulfides utilizing O_2_ as an oxidant under light irradiation represents a green, mild, and sustainable approach, which has garnered significant attention. Therefore, we primarily selected these two types of aerobic photocatalytic reactions as model reactions to study the photocatalytic performance of **EtNBS‐H** as a red‐light‐absorbing photocatalyst, providing insights for the development of photocatalysts applicable to other aerobic photocatalytic processes. We first investigated the potential application of **EtNBS‐H** in the hydroxylation of arylboronic acids (Figure S17). In reactions, a sacrificial donor, for example, TEOA, is typically required to supply electrons that reduce the oxidized photocatalyst. Its evidently lower p*K*_a_ value of 7.82, compared to that of **EtNBS‐H**, ensures the structural stability of the photocatalyst in the presence of TEOA. Encouraged by the water solubility of **EtNBS‐H**, we selected 4‐carboxyphenylboronic acid as a substrate (5 mM) to perform the synthesis of 4‐hydroxybenzoic acid in an aqueous medium of **EtNBS‐H** (1 mol%) and TEOA (50 mM). As shown in Table 1, under red light irradiation (660–665 nm, 45.6 mW·cm^−2^), the conversion of 4‐carboxyphenylboronic acid in water can be up to > 99% towards 4‐hydroxybenzoic acid by optimizing the concentration of **EtNBS‐H** from 0 mol% to 2 mol%. No conversion of arylboronic acids is observed without the photocatalyst, highlighting its crucial role in the system (Figure S18). Moreover, it is found that the red‐light‐driven photooxidation can boost the catalytic efficiency by 1.62‐fold compared to that activated by white light (420–650 nm, 80.5 mW·cm^−2^), which highlights the advantage of **EtNBS‐H** for red‐light‐driven photocatalysis in the aspect of conversion and energy‐saving effectiveness.

**TABLE 1:** The reaction condition optimization for aerobic oxidative hydroxylation of arylboronic acids.

Entry	Light	EtNBS‐H (mol%)	Solvent	Conversion (%)
1	White	1	Water	42
2	Red	0	Water	0
3	Red	1	Water	68
4	Red	1.5	Water	90
5	Red	2	Water	>99

**FIGURE 4:**
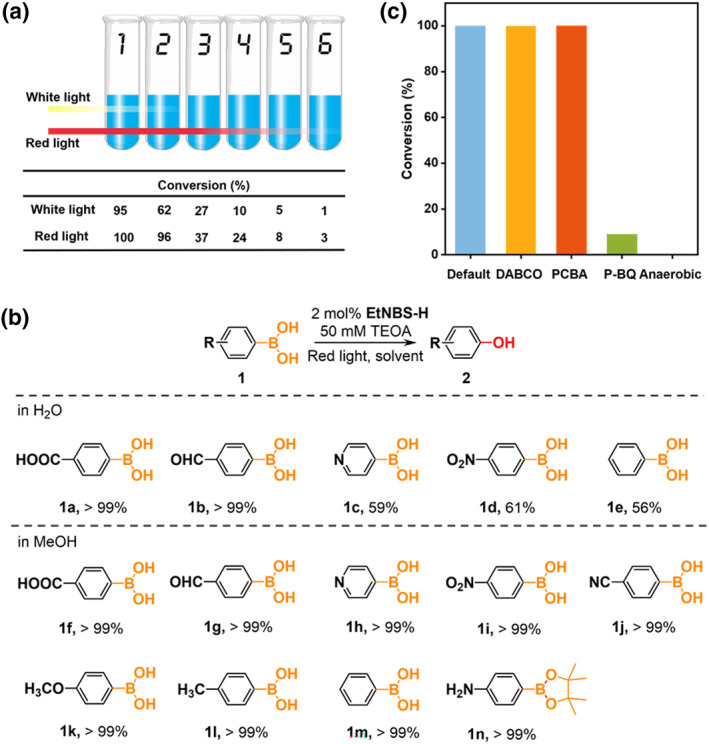
Penetration effect and aerobic oxidative hydroxylation of arylboronic acids. (a) Penetration effect of white light and red light irradiation (2 h) assessed by evaluating the aerobic oxidative hydroxylation reaction conversion of 4‐carboxyphenylboronic in 6 bundled reaction tubes, utilizing **EtNBS‐H** as the photocatalyst. (b) The scope of substrates for aerobic oxidative hydroxylation of arylboronic acids. Reaction condition: substrate 1 (5 mM), TEOA (50 mM), and **EtNBS‐H** (2 mol%) in H_2_O (2 mL) or MeOH (2 mL) were irradiated with red light for 2 h at room temperature under ambient atmosphere. Reaction conversion was determined by ^1^H NMR analysis. (c) Reaction conversion of aerobic oxidative hydroxylation of arylboronic acids with or without reactive oxygen species (ROS) quenchers.

Encouraged by the success of the red‐light‐driven photooxidation using **EtNBS‐H**, we sought to further compare the conversion of 4‐carboxyphenylboronic upon irradiation with a white light versus a red light source. This experimental study aims to emphasize the penetration depth of long‐wavelength lights into reaction media, thereby potentially enhancing the scalability of photocatalysis for industrial applications. Specifically, a row of 6 reaction tubes was arranged together, with the light source exclusively irradiating the tubes from the left side while all other sides were shielded using aluminum foil. The conversion in each reaction tube after 2 h of irradiation at either white light or red light is depicted in Figure 4a and Figures S19–S20. The white light source induced a decent conversion of 95% in the first tube, whereas the second one demonstrated a significantly decreased conversion of 62%. In contrast, the utilization of red light led to an exceptional conversion >99% in the first tube, while also achieving a satisfactory conversion of 96% in the second tube. Particularly, although the conversion of the remaining tubes from the 3rd to the 5th decreases after irradiation with either white light or red light, the photocatalytic performance driven by red light surpasses that initiated by white light. Especially, the optical power density measurements revealed a more pronounced decline in transmitted white light intensity compared to red light in the first two test tubes, which is consistent with the reduction trend in photocatalytic conversion rates (Table S2). This phenomenon clearly demonstrates the advantageous ability of **EtNBS‐H** to absorb red light.

**FIGURE 5:**
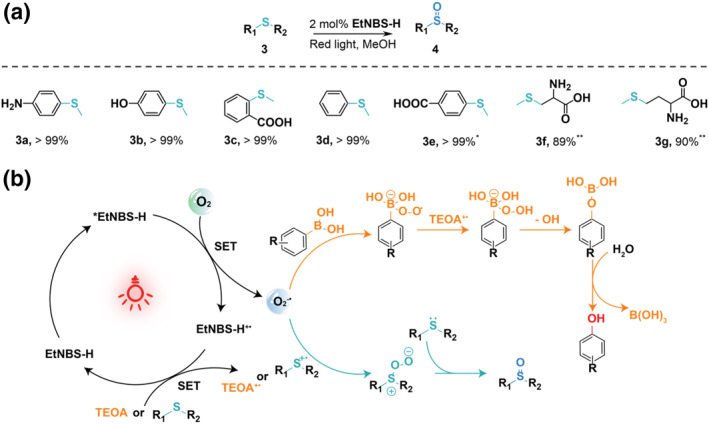
Photooxidation of sulfur ether and proposed mechanism. (a) Substrate scope for thioether oxidation. Reaction condition: substrate 3 (5 mM) and **EtNBS‐H** (2 mol%) in MeOH (2 mL) were irradiated with red light for 2 h at room temperature under ambient atmosphere. Reaction conversion was determined by ^1^H NMR analysis. *The irradiation time was 4 h.** Reaction solvent was H_2_O. (b) Proposed mechanism for the aerobic oxidative hydroxylation and oxidation of sulfur ether.

After optimizing the reaction conditions, our investigation was expanded to include a diverse array of arylboronic acid substrates. Taking advantage of the water solubility of **EtNBS‐H**, we examined the catalytic activity of **EtNBS‐H** in aqueous environments towards other substrates. As shown in Figure 4b and Figures S21–S25, for water‐soluble substrates, the conversion of the photocatalytic reaction under red light can be above 99% in aqueous environments. Therefore, **EtNBS‐H** demonstrates a promising prospect for achieving highly efficient photocatalytic reactions in green aqueous environments. For substrates that are not soluble in water, methanol can be used as a solvent instead, and the conversion of the reaction can also be above 99% (Figure 4b and Figures S26–S34). These experiments confirm the high effectiveness of this photocatalyst in using red light to catalyze the hydroxylation of arylboronic acids. To clarify the significance of ROS in driving aerobic photocatalysis, control experiments were conducted employing diverse ROS quenchers (Figure 4c and Figure S35). The presence of triethylenediamine (DABCO) and 4‐chlorobenzoic acid (PCBA), as quenchers for ^1^O_2_ and •OH, respectively, was found to have negligible impact on the conversion of 4‐carboxybenzene boronic acid. In a sharp contrast, upon the addition of *p*‐benzoquinone (*p*‐BQ), an O_2_^−•^ quencher, the conversion decreased from >99% to 9% after 2 h irradiation at red light, highlighting the necessity of O_2_^−•^ in the photocatalytic oxidative hydroxylation of arylboronic acids. We thus propose a plausible mechanism that adequately reflects the involvement of red‐light absorption and the type‐I pathway for photocatalytic hydroxylation of arylboronic acids (Figure 5b).

### 2.5 Photooxidation of sulfur ether and other substates

To further evaluate the broad applicability of **EtNBS‐H** as a photocatalyst for red‐light‐driven photooxidation, the transformations of thioethers and other substrates were investigated (Figure 5a and Figures S36–S42). Under red light irradiation, the conversion of thioethers (5 mM) catalyzed by **EtNBS‐H** easily exceeds 99% (**4a‐e**). Additionally, utilizing the identical light source and photocatalyst, the synthesis of sulfoxides from water‐soluble substrates can be realized in aqueous solutions with remarkable efficiency (**4f‐g**). Figure S43–S44 clearly demonstrates that the absence of O_2_ and the presence of O_2_^−•^ quencher obviously decrease the conversion, thereby suggesting an electron transfer photocatalytic mechanism for the oxidation of thioethers. Besides arylboronic acids and thioethers, the red‐light‐driven photooxidation by **EtNBS‐H** was also exploited in another two specific reactions: the bromination of aromatic compounds and the oxidation of *α*‐pinene. Under mild ambient conditions, the organic synthesis demonstrated highly efficient conversion to brominated aromatics and 4‐isopropyltoluene, respectively, with conversion ranging from 80% to 99% (Figures S45–S47). These results highlight the significance of **EtNBS‐H** in facilitating photocatalytic oxidation by generating oxygen radicals via the type‐I pathway (Figure 5b).

### 2.6 Recoverability and recyclability of the photocatalyst and gram‐scale of the photocatalytic reactions

**FIGURE 6:**
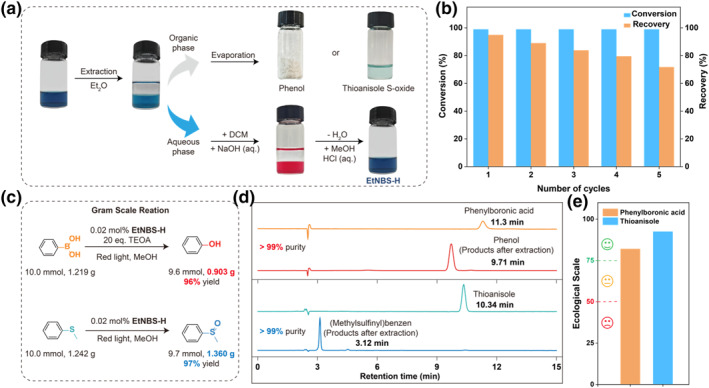
Recoverability and recyclability of the **EtNBS‐H** and gram‐scale of the photocatalytic reactions. (a) Acid‐base reaction color changes and recovery process of **EtNBS‐H**. (b) Recyclability tests of **EtNBS‐H** in the oxidation of sulfur ether. (c) Gram‐scale synthesis of phenol and sulfoxide by **EtNBS‐H**. (d) HPLC spectra of the product obtained by extraction after photocatalysis with phenylboronic acid (detection wavelength: 254 nm, methanol:water = 30:70, v/v) and thioanisole (detection wavelength: 254 nm, methanol:water = 60:40, v/v). (e) Ecological scale calculations of the photocatalytic reaction (>75, Excellent; >50, Acceptable; <50, Inadequate).

The primary challenges in developing homogeneous organic molecules as cost‐effective and eco‐friendly photocatalysts lie in ensuring the efficient and convenient separation and purification of reaction products, while also maintaining the photostability and recyclability of the photocatalysts for use. The photoinduced degradation of photocatalysts would lead to diminished catalytic performance and reduced recovery rates in catalyst recycling and reusing processes. Fortunately, red light, with lower energy compared to ultraviolet and short visible lights, offers a solution to mitigate photobleaching effects caused by high‐energy photons. For this reason, the absorption of **EtNBS‐H** displayed negligible changes after exposure to red light for 2 h, highlighting its potential in maintaining integrity during photocatalysis (Figure S48). To enable both high‐purity product separation and efficient photocatalyst recovery, we herein developed a feasible and straightforward method based on reversible acid‐base reaction on the iminium group of **EtNBS‐H**. Firstly, as a hydrochloride salt, **EtNBS‐H** and its corresponding base product display different solubilities in immiscible solvents, such as water and diethyl ether (Et_2_O). By simply adjusting the pH after the photocatalytic reaction, the photocatalyst can be toggled between its protonated and deprotonated states, allowing for straightforward liquid‐liquid extraction that effectively separates the photocatalyst from the reaction products. This method not only guarantees easy recovery and reuse of the photocatalyst, but also enables the efficient purification of the target products (if the selectivity of photocatalysis reaction is sufficiently high). Secondly, **EtNBS‐H** exhibits distinct differences in absorption compared to the base product, thereby leading to a significant change in solution color from blue to orange after the acid‐base reaction (Figure 6a and Figure S49). Such visually appearance alterations facilitate catalyst recycling by enabling easy observation by naked eyes during the extraction. To convert the photocatalyst into its corresponding base, an aqueous solution of the used catalyst **EtNBS‐H** was titrated with a sodium hydroxide solution. The base product was successfully transferred from the aqueous phase to dichloromethane (DCM) through extraction. Subsequently, we obtained the recovered catalyst **EtNBS‐H** by acidification and vacuum drying for repeating use, achieving a consistent recovery rate of over 94% over each catalytic cycle and maintaining a total recovery of over 70% after 5 cycles (Figure 6b, Figure S50, and Table S3). Importantly, the conversion of thioanisole in 5 cycles were all steady (>99%), suggesting an excellent photocatalytic activity of the recovered **EtNBS‐H** (Figure 6b and Figure S51).

To further demonstrate the potential of **EtNBS‐H** in the large‐scale photocatalytic organic synthesis applications, gram‐scale reactions were performed using phenylboronic acid and thioanisole as typical substrates. In the gram‐scale experiments, the substrate concentration was increased by a factor of 100 (10 mmol, 0.5 M), while the concentration of **EtNBS‐H** remained unchanged (100 μM, 0.02 mol%). Remarkably, the isolated product yields of phenol and thioether following liquid‐liquid extraction after 24 h of red light irradiation were 96% and 97%, respectively (based on weight, Figure 6c). Both high performance liquid chromatography and ^1^H NMR analysis confirmed that the purity of the products obtained from these reactions was more than 99% (Figure 6d and Figures S52–S54). Specifically, the retention time for phenylboronic acid was observed at 11.3 min. After the involvement of **EtNBS‐H** in the photocatalytic oxidative hydroxylation of phenylboronic acid, the corresponding signal disappeared, and a new signal corresponding to phenol appeared at 9.71 min, with no additional impurity peaks detected. Similar results are also obtained in the photocatalytic oxidation reaction of thioanisole. The calculations using established methods revealed that even a low concentration (0.2 mmol%) of **EtNBS‐H** can also result in satisfactory turnover numbers (TON) and turnover frequencies (TOF), outperforming other homogeneous photocatalysts,^[17]^ and indicating a high catalytic efficiency of **EtNBS‐H** in the photooxidation process (Figure S55). The excellent ecological scale parameters of 82 and 92.5 further highlight the environmentally friendly nature of the photocatalytic systems based on **EtNBS‐H**, reinforcing its position as an exemplary green synthesis approach from both commercial and ecological perspectives (Figure 6e and Tables S4–S5). In essence, this protocol signifies a sustainable and commercially potential method, aligning with green synthesis principles while promoting efficiency and purity in photocatalytic reactions.

## 3 CONCLUSION

This work demonstrated that **EtNBS‐H**, a recyclable type‐I photocatalyst, effectively facilitated red‐light‐driven photooxidation reactions with superior catalytic efficiency, scalability, and recovery rates. Upon irradiation with red light, such an organic photocatalyst exclusively produced O_2_^−•^, achieving the hydroxylation of arylboronic acids, and oxidization of thioethers and other substrates with conversion as high as > 99%. The recyclability of **EtNBS‐H** was easily accomplished through a simple pH‐tunable acid‐base reaction, allowing for easy separation and reuse of the photocatalyst with a consistent recovery rate above 94% over each catalytic cycle. Moreover, the efficient absorption of red light by **EtNBS‐H**, which has a greater ability to penetrate the reaction medium compared to shorter wavelengths, enhances the photocatalytic efficiency and renders the **EtNBS‐H**‐mediated photocatalytic process scalable for industrial applications. Thus, the study successfully validated the scalability and commercial viability of **EtNBS‐H** through gram‐scale reactions and favorable metrics in green chemistry. The demonstrated efficacy, scalability, and recyclability of **EtNBS‐H** in organic transformations position it as a valuable asset for future practical applications in green synthesis.

## CONFLICT OF INTEREST STATEMENT

The authors declare no conflicts of interest.

## ETHICS STATEMENT

This study did not involve human participants, animal experiments, or any sensitive data collection. All experimental procedures and data analyses were conducted in compliance with institutional and national research ethics standards.

## Supporting information

Supporting Information S1

## Data Availability

The data that support the findings of this study are available from the corresponding author upon reasonable request.
