# Supplementary material for: A recyclable type‐I photosensitizer to enable red‐light‐driven gram‐scale aerobic photocatalysis
Source: Smart Mol. 2025 Jun 4;3(3):e20250003. doi: 10.1002/smo.20250003 (PMC12483128; doi:10.1002/smo.20250003)
Supplement: Supplementary file 1 — Supporting Information S1 [file SMO2-3-e20250003-s001.docx]

Supporting Information

A recyclable type-I photosensitizer to enable red-light-driven gram-scale aerobic photocatalysis

Shirong Yan^a^, Lu Qiao^a^, Lei Chen, Wu-Jie Guo, Hui-Qing Peng*

S. Yan, L. Qiao, L. Chen, W.-J. Guo, H.-Q. Peng

Beijing Advanced Innovation Center for Soft Matter Science and Engineering

Beijing University of Chemical Technology

Beijing 100029, China

E-mail: hqpeng@mail.buct.edu.cn

**Materials and Methods**

Unless stated otherwise, all analytical grade chemicals and solvents used in this paper were purchased from commercial vendors. The organic solvents are analytically pure. ^1^H NMR and ^13^C NMR spectra were obtained on a Bruker Advance ΙΙΙ spectrometer. UV-vis spectra. Photoluminescence (PL) spectra, molar absorption values were measured on a Shimadzu UV-2600i spectrophotometer and a Shimadzu RF-6000 spectrophotometer, respectively. High resolution mass spectrometry (HR-MS) measurements were performed on a Waters UPLC/Premier mass spectrometry. High performance liquid chromatography (HPLC) spectra were measured on a Agilent 1260 Infinity II using a Sapphiresil C18 column. ROS testing using PLS-SXE300 Xenon lamp (15 mW·cm^-2^) and red light (λ = 660-665 nm, 45.6 mW·cm^-2^). White light (30 W, 18-28 V, 1200 mA, λ = 420-650 nm, 80.5 mW·cm^-2^) and red light (20 W, 10-18 V, 1200 mA, λ = 660-665 nm, 45.6 mW·cm^-2^) were used as the irradiation light source for photocatalytic experiment, and Pyrex tubes were used as the reaction vessel. The distance from the white/red light to Pyrex tubes is less than 5 mm. No filter was used during irradiation.

**Detection of ROS production.** DCFH was used as an indicator for the detection of ROS in the solution. When ROS is generated in the system, the non-fluorescent DCFH will be oxidized and emit obvious fluorescence at 525 nm. **EtNBS-H** (5 μM) were dissolved in 2 mL water containing 1 μM of DCFH. The mixture was then placed in a cuvette and irradiated under Xenon lamp irradiation (15 mW·cm^-2^). The fluorescence intensity change of the sample at 525 nm was recorded by the fluorescence spectrometer.

**Detection of ^1^O_2_ production.** ABDA was used as an indicator to evaluate ^1^O_2_ generation of **EtNBS-H** in solution. ABDA (100 μM) solution was mixed with the **EtNBS-H** in water and then exposed to red light (45.6 mW·cm^-2^) irradiation. The absorbance decrease of ABDA at 378 nm was recorded at various irradiation time.

**Detection of O_2_^−•^ production.** DHR123 was used as an indicator for the detection of O_2_^−•^ in solution. When O_2_^−•^ is generated in the system, the DHR 123 will be oxidized and emit strong fluorescence centered at 526 nm. 5 μM of **EtNBS-H** were dissolved in 2 mL water containing 10 μM of DHR 123. The mixture was then placed in a cuvette and irradiated under red light (45.6 mW·cm^-2^) irradiation. The fluorescence change of the sample at 526 nm was recorded by the fluorescence spectrometer.

**Electron paramagnetic resonance (EPR) Measurement.** EPR measurement was used to identify the type of ROS using 5,5-dimethyl-1-pyrroline-N-oxide (DMPO) as the radical indicator. Samples were prepared by mixing 50 μL, 1 mM of **EtNBS-H** and 10 μL of DMPO (200 mM) in methanol. EPR signals were recorded by adding samples through a capillary tube under Xenon lamp (15 mW·cm^-2^) irradiation at for 2 min.

**Theoretical Calculations.** The ground-state geometries were optimized using B3LYP density function theory (DFT) at the basis set level of 6-311+G^*^. The excited-state geometries in water were optimized using the time-dependent density function theory (TD-DFT) method, with M06-2X hybrid functional at the basis set level of 6-311+G^*^, performed using Gaussian 09, supported by high performance computing platform of BUCT. To understand how interactions between **EtNBS-H** and O_2_, more than 200 initial structures were generated using the Molclus software package, which served as the initial structures for a semiempirical quantum mechanical optimization at the PM6-DH+ using the MOPAC program. The dimer with the lowest energy was utilized as a candidate structure and further optimized by the all-electron DFT method. Basis set superposition (BSSE) corrections were applied to calculate interaction energies.

**Aerobic Oxidative Hydroxylation of** **Arylboronic Acids.** Conditions for the aerobic oxidative hydroxylation reaction of the arylboronic acid series: arylboronic acid substrate (5 mM), TEOA (50 mM), and **EtNBS-H** (2 mol%) in solution (2 mL) were irradiated with white light or red light for 2 h. The solvent was spin-dried and then deuterium reagent was added, and the conversion was determined by ^1^H NMR. In the scavenging control experiment, the concentration of quencher was 25 mM.

**P****hotooxidation of Thioethers.** Conditions for the photooxidation of thioether series: thioether substrate (5 mM) and **EtNBS-H** (2 mol%) in solution (2 mL) were irradiated with red light for 2 h. The solvent was spin-dried and then deuterium reagent was added, and the conversion was determined by ^1^H NMR. In the scavenging control experiment, the concentration of quencher was 25 mM.

**Bromination of Aromatic Compounds.** Conditions for the bromination of aromatic compounds: aromatic compounds substrate (5 mM), HBr (6 mM), and 2 mol% **EtNBS-H** in acetonitrile solution (2 mL) were irradiated with red light for 2 h. The solvent was spin-dried, and deuterium reagent was added, and the conversion was determined by ^1^H NMR.

**Oxidation of α-pinene.** Conditions for the oxidation of α-pinene: α-pinene (5 mM) and **EtNBS-H** (2 mol%) in acetonitrile solution (2 mL) were irradiated with red light for 2 h. After spin-drying the solvent, deuterium reagent was added, and the conversion was determined by ^1^H NMR.

**Recoverability and Recyclability of EtNBS-H.** To determine the recoverability and recyclability of **EtNBS-H**, the photooxidation reaction of thioanisole (5 mM) in 20 mL of methanol was chosen as a model reaction, using 2 mol% **EtNBS-H** as the photocatalyst. After the completion of the reaction, the solvent was evaporated under reduced pressure and subsequently extracted with ether and water. At this stage, the reaction product was in the organic layer and the photocatalyst remained in the aqueous layer. The organic layer portion was then separated, and the extraction continued using an aqueous solution of NaOH (pH = 12) and 20 mL of dichloromethane. Finally, the organic layer was collected to quantify the change in UV absorption of the photosensitizer for assessing the recoverability of **EtNBS-H**. After measurement, the solution was subjected to evaporation and subsequently treated with MeOH and acidified using1 drop of hydrochloric acid until the solution exhibited a blue color. **EtNBS-H** was reused for the next thioether oxidation cycle. The same procedure was followed for four cycles. A linear relationship between **EtNBS-H** concentration and absorbance was obtained based on Rombauer's law. The absorbance was brought to obtain the actual **EtNBS-H** concentration, which was divided by the theoretical **EtNBS-H** concentration to obtain the recovery.

**Gram-scale reaction.** Phenylboronic acid (10 mmol, 0.5 M), TEOA (1 M), and **EtNBS-H** (100 μM, 0.02 mol%) were dissolved in methanol (20 mL) and irradiated with red light for 24 h. Similarly, thioanisole (10 mmol, 0.5 M) and **EtNBS-H** (100 μM, 0.02 mol%) were dissolved in methanol (20 mL) and irradiated with red light for 24 h. Upon completion, the reaction solution was extracted with diethyl ether (Et_2_O) and water. The organic layer contained the reaction product, while the photocatalyst remained in the aqueous layer. The Et_2_O was then evaporated under reduced pressure to obtain the product. The isolated yield of the product was determined by weighing after extraction, and its purity was assessed by ¹H NMR and HPLC.

**Evaluation of green chemistry metrics.** In order to explore the greater potential of **EtNBS-H**, its equivalent was further reduced to 0.2% mmol. Surprisingly, the reaction still proceeded well, and based on this, the green chemistry parameters were calculated. The green chemistry metrics including Turnover Number (TON) and Turnover Frequency (TOF) were calculated according to the reported method. EcoScale was calculated according to the reported method.

$TON = \frac{Amout of desired product (mmol)}{Amout of catalyst used (mmol)}$ (1)

$TOF = \frac{TON}{\mathrm{Time}\left( \mathrm{hour} \right)}$ (2)

**Synthesis and Characterization**

***Synthesis of compound Ⅰ.*** *N,N*-diethyl-p-phenylenediamine (1.000 g, 12.195 mmol), aluminum sulfate (4.464 g, 13.04 mmol), sodium thiosulfate (6.938 g, 28 mmol), zinc chloride (1.743 g, 12.78 mmol), and 20 mL of water were placed into a round-bottomed flask and cool in an ice bath with stirring. Potassium dichromate (0.989 g, 3.36 mmol) was dissolved in 10 mL of water and slowly added to the reaction flask over a period of 20 min, and the reaction was continued in an ice bath for 2 h. The reaction was then filtered, and the solid product was rinsed with acetone. The solid product was added to 20 mL of methanol, heated to reflux in a water bath and stirred for 1 h. It was filtered and dried to obtain the light green compound I.

***Synthesis of compound Ⅱ.*** 1-Naphthylamine (1.430 g, 10 mmol), 3-bromopropargyl (1.190 g, 10 mmol), K_2_CO_3_ (1.380 g, 10 mmol), and 25 mL of *N,N*-dimethylformamide were placed in a round-bottomed flask and stirred at room temperature for 12 h. The product was purified by column chromatography using 1% ethyl acetate in petroleum ether as eluent to obtain an orange oily liquid compound Ⅱ (1.087 g, 60%). ^1^H NMR (400 MHz, Chloroform-*d*)): δ 7.93 (d, *J* = 8.1 Hz, 1H), 7.85 (d, *J* = 8.3 Hz, 1H), 7.61-7.48 (m, 3H), 7.46 (d, *J* = 8.2 Hz, 1H), 6.82 (d, *J* = 7.4 Hz, 1H), 4.16 (d, *J* = 2.4 Hz, 2H), 2.40 (q, *J* = 2.3 Hz, 1H). ^13^C NMR (101 MHz, Chloroform-*d*): δ 142.21, 134.37, 128.81, 126.52, 126.00, 125.13, 123.92, 120.13, 118.87, 105.60, 81.09, 71.85, 33.95. HR-MS: m/z ([C_13_H_11_N] ^+^) = 182.0992; calcd =181.0891.

***Synthesis of EtNBS-H.*** Compound I (298 mg, 1.0 mmol), 1-Naphthylamine (258 mg, 1.8 mmol), and 25 mL of methanol were placed in a round bottom flask. The reaction mixture was heated to reflux. Silver carbonate (606 mg, 2.2 mmol) was added slowly and a strong color change was observed within 5 min. The reaction was continued at reflux for 30 min and then the reaction flask was cooled to room temperature. The mixture was filtered and spun dry. The crude product was dissolved in 25 mL of dichloromethane and washed with saturated sodium carbonate solution to dryness. The organic phase was filtered and acidified with 0.4 mL of concentrated hydrochloric acid. The product was purified by column chromatography using 2-10% methanol in dichloromethane as eluent to obtain a dark blue solid (133 mg, 36%). ^1^H NMR (400 MHz, Chloroform-*d*)): δ 8.71 (dd, *J* = 7.7, 1.7 Hz, 1H), 8.32 (dd, *J* = 7.5, 1.8 Hz, 1H), 7.66-7.57 (m, 2H), 7.55 (d, *J* = 9.0 Hz, 1H), 6.68 - 6.60 (m, 2H), 6.40 (d, *J* = 2.8 Hz, 1H), 3.41 (q, *J* = 7.1 Hz, 4H), 1.23 (t, *J* = 7.1 Hz, 6H). ^13^C NMR (101 MHz, Chloroform-*d*)): δ 162.94, 148.14, 140.52, 134.02, 133.56, 131.57, 131.12, 130.01, 129.63, 129.54, 125.32, 124.99, 123.95, 119.04, 111.15, 105.18, 44.68, 12.70. HR-MS: m/z ([C_20_H_20_N_3_S]^+^) = 334.1510; calcd =334.4605.

***Synthesis of EtNBS-Alk.*** Compound I (298 mg, 1.0 mmol), compound Ⅱ (325.8 mg, 1.8 mmol), and 25 mL of methanol were placed in a round bottom flask. The reaction mixture was heated to reflux. Silver carbonate (606 mg, 2.2 mmol) was added slowly and a strong color change was observed within 0-5 min. The reaction was continued at reflux for 30 min and then the reaction flask was cool to room temperature. The mixture was filtered and spun dry. The crude product was dissolved in 25 mL of dichloromethane and washed with saturated sodium carbonate solution to dryness. The organic phase was filtered and acidified with 0.4 mL of concentrated hydrochloric acid. The product was purified by column chromatography using 2-10% methanol in dichloromethane as eluent to obtain a dark blue solid (163 mg, 40%). ^1^H NMR (400 MHz, Methanol-*d_4_*): δ 8.76 (s, 1H), 8.01 (d, *J* = 8.2 Hz, 1H), 7.77 (dd, *J* = 10.4, 8.4 Hz, 2H), 7.68 (q, *J* = 7.5, 7.0 Hz, 1H), 7.29 (dd, *J* = 9.6, 2.7 Hz, 1H), 7.13 (s, 1H), 7.05 (d, *J* = 2.6 Hz, 1H), 4.42 (d, *J* = 2.4 Hz, 1H), 3.66 (q, *J* = 7.1 Hz, 4H), 3.04 (t, *J* = 2.4 Hz, 1H), 1.35 (t, *J* = 7.2 Hz, 6H). ^13^C NMR (101 MHz, Methanol-*d_4_*): δ 152.10, 151.61, 139.13, 137.37, 133.55, 133.15, 132.71, 130.66, 129.24, 124.75, 123.89, 121.81, 118.23, 104.83, 101.72, 77.71, 73.49, 53.75, 45.73, 32.56, 11.78. HR-MS: m/z ([C_23_H_22_N_3_S]^+^) = 372.1500; calcd =372.1529.


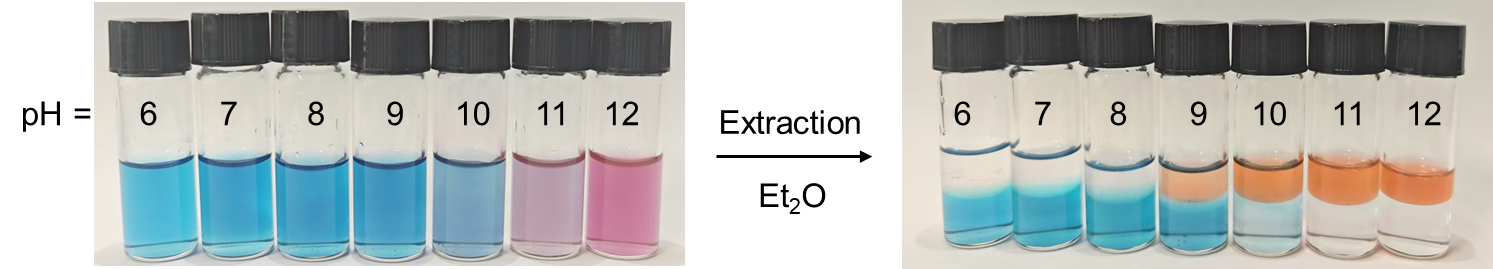


**Figure S1.** The color changes of **EtNBS-H** (100 μM) in water and after extraction with Et_2_O at different pH values.


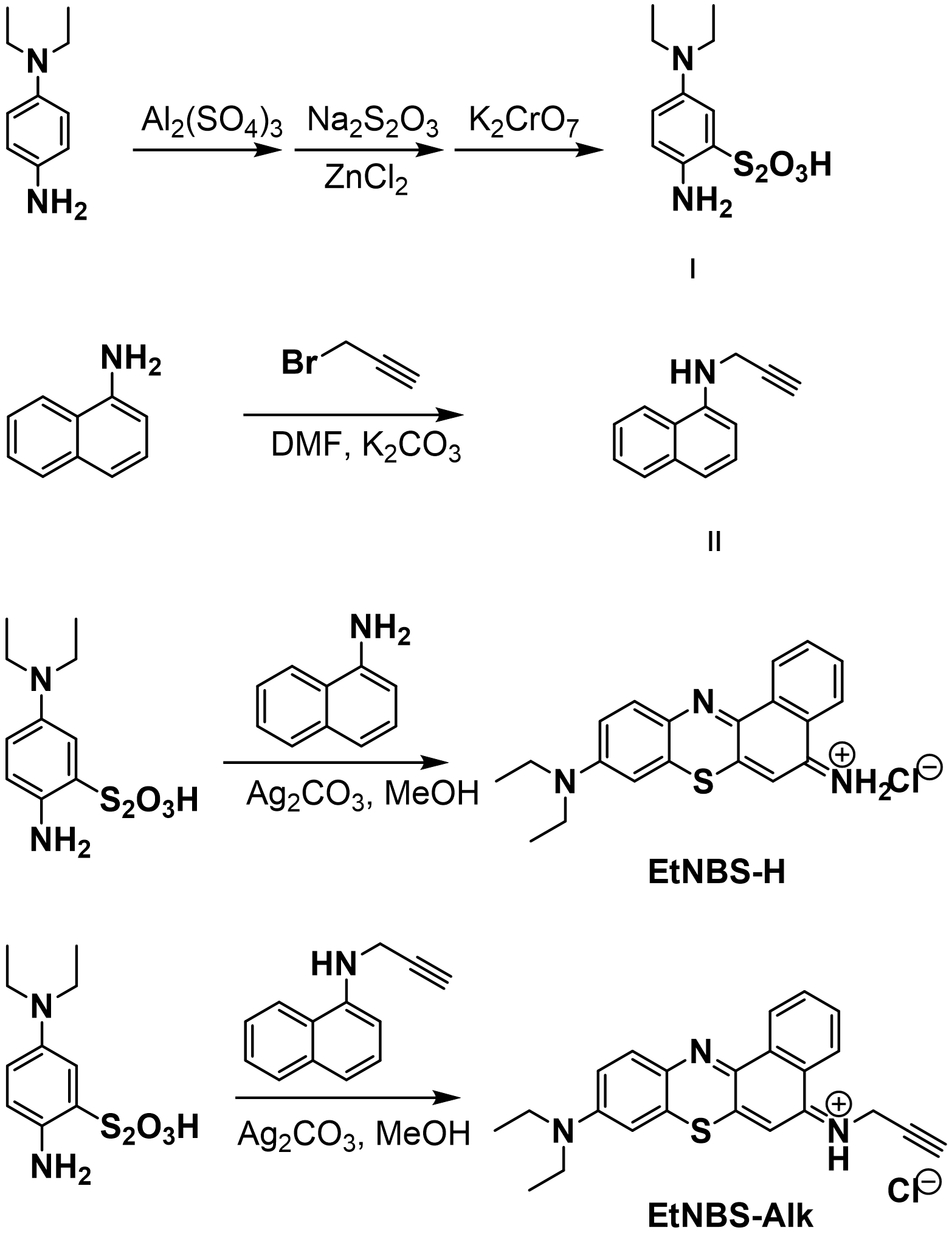


**Figure S2.** The synthesis of **EtNBS-H** and **EtNBS-Alk**.

**
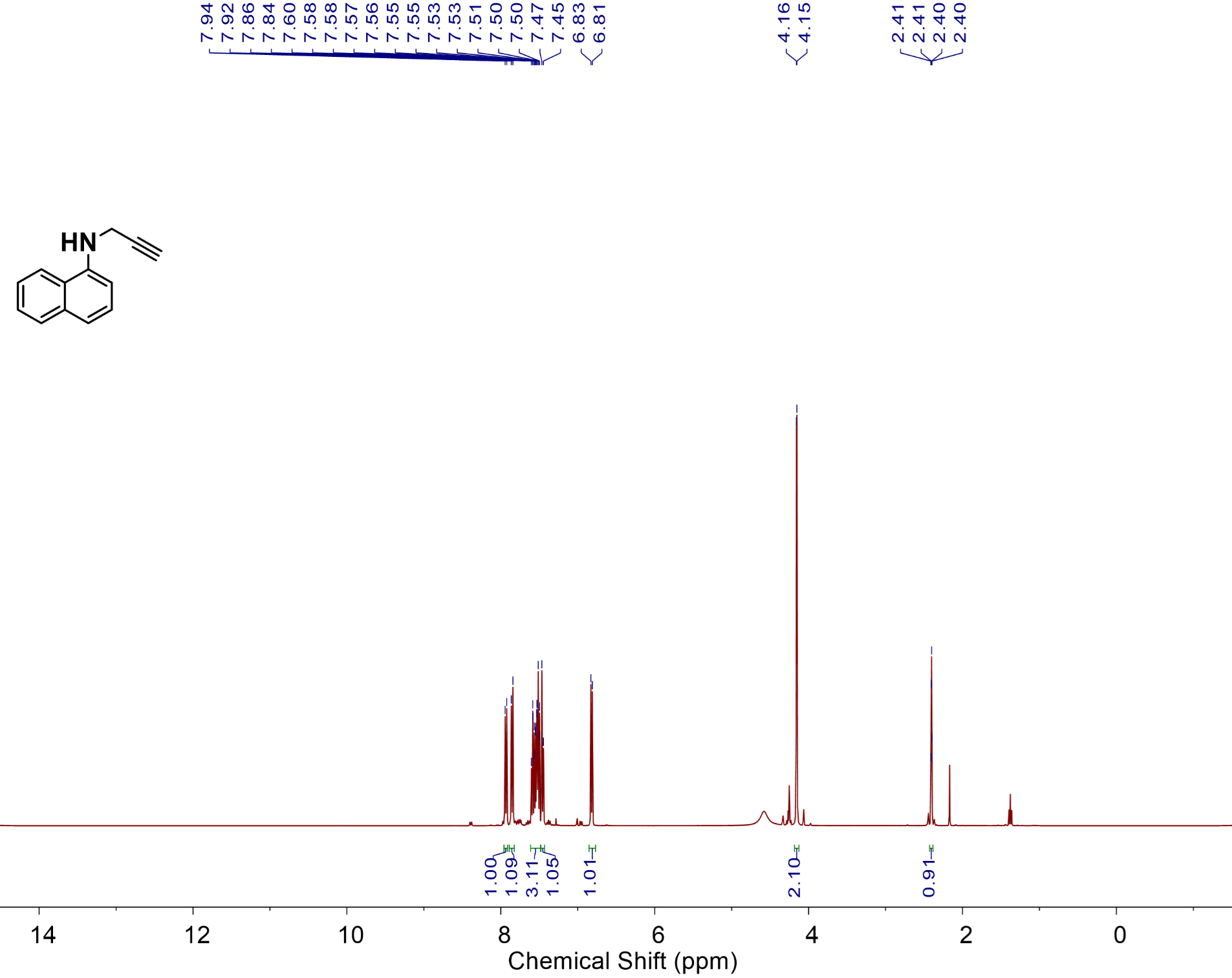
**

**Figure S3.** ^1^H NMR spectrum of compound Ⅱ in CDCl_3_.


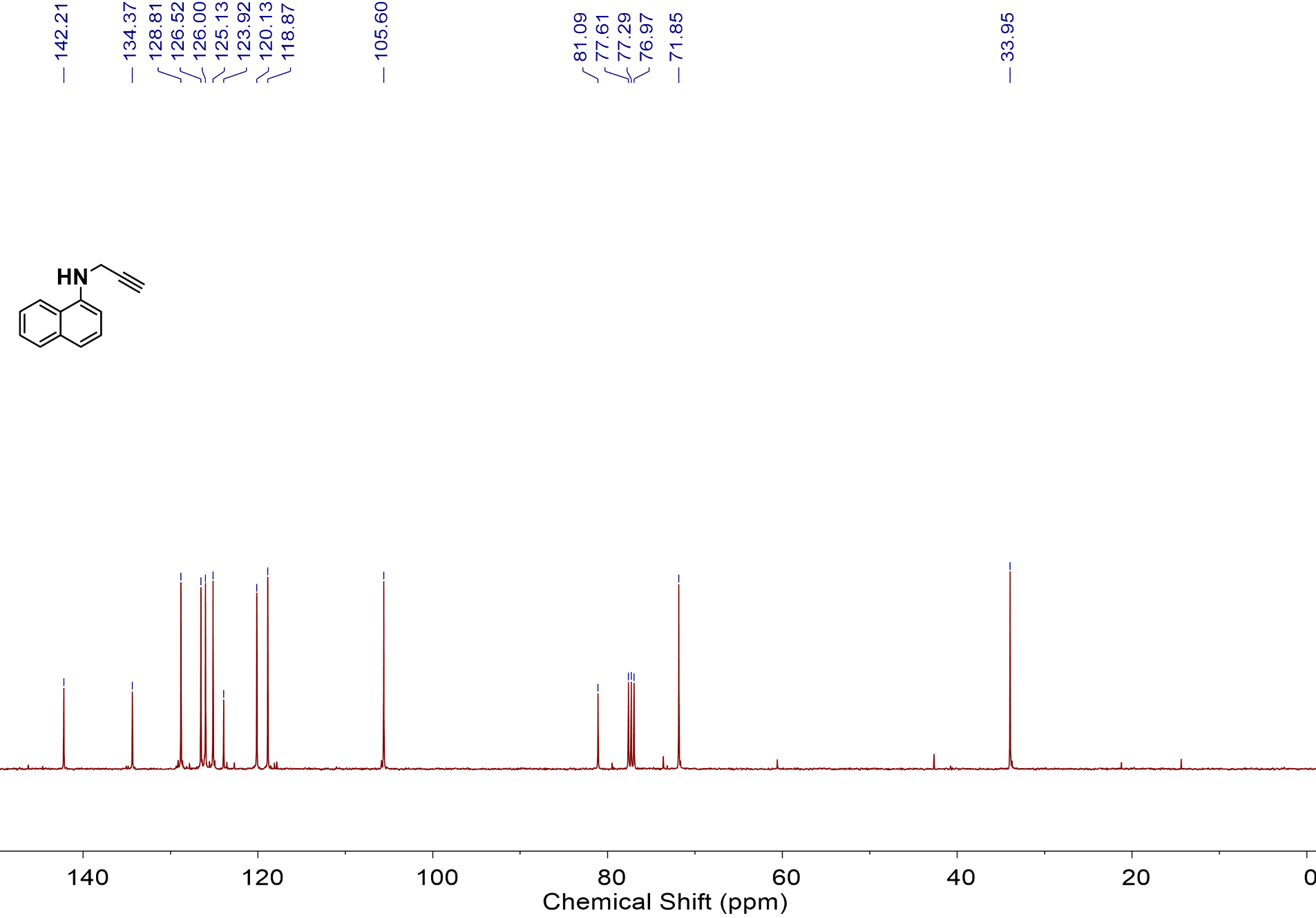


**Figure S4.** ^13^C NMR spectrum of compound Ⅱ in CDCl_3_.

**
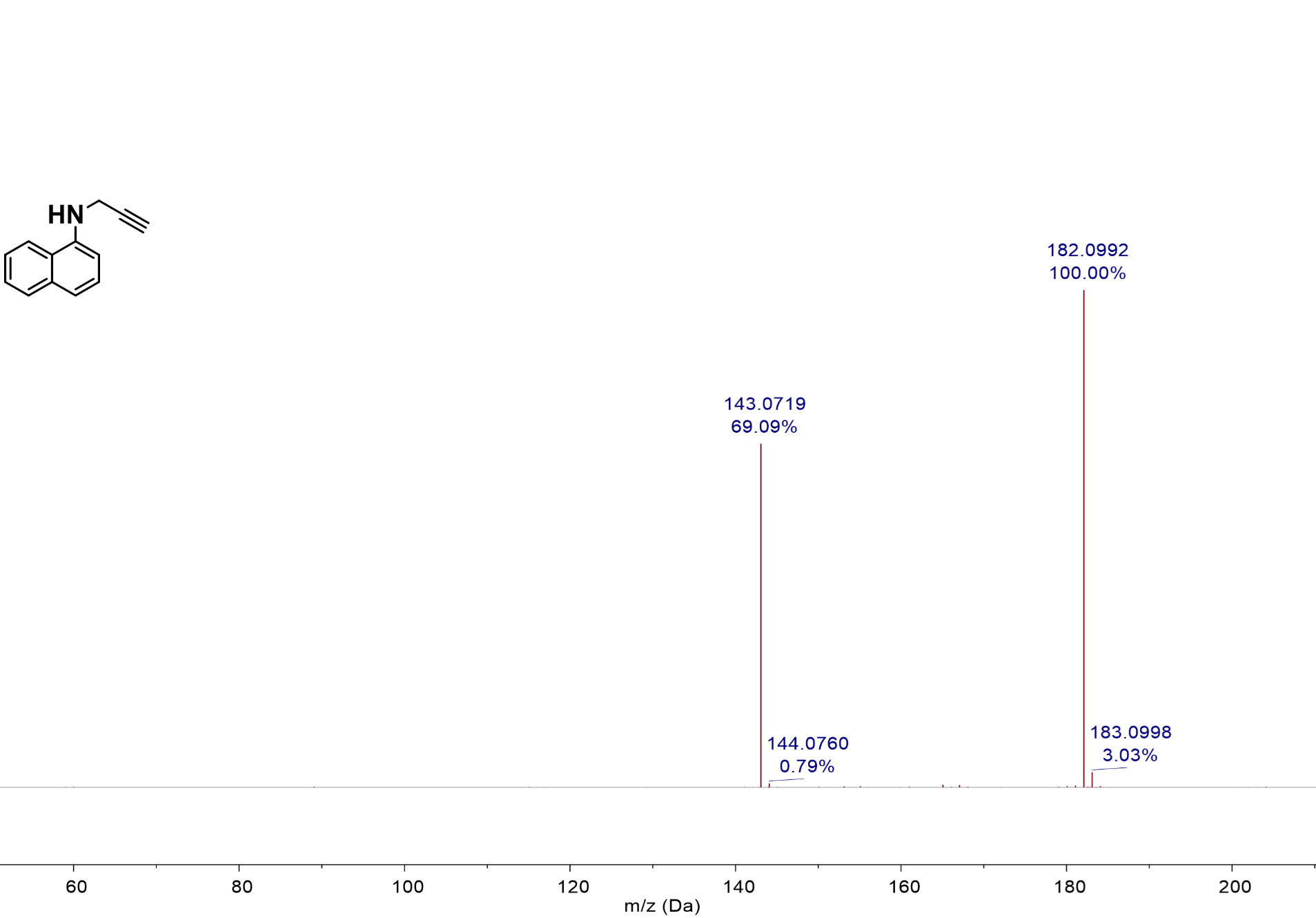
**

**Figure S5.** HR-MS spectrum of compound Ⅱ.


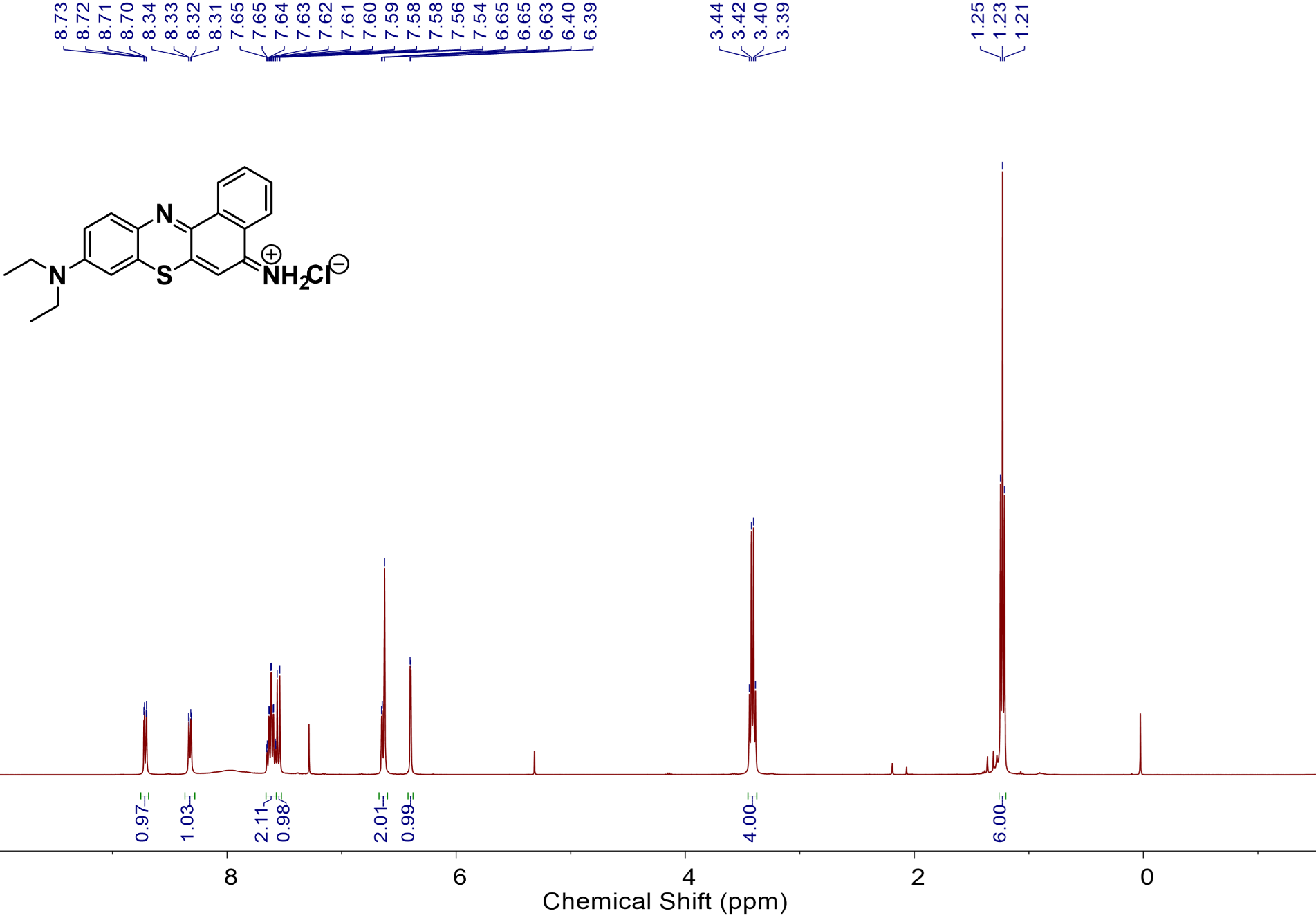


**Figure S6.** ^1^H NMR spectrum of **EtNBS-H** in CDCl_3_.


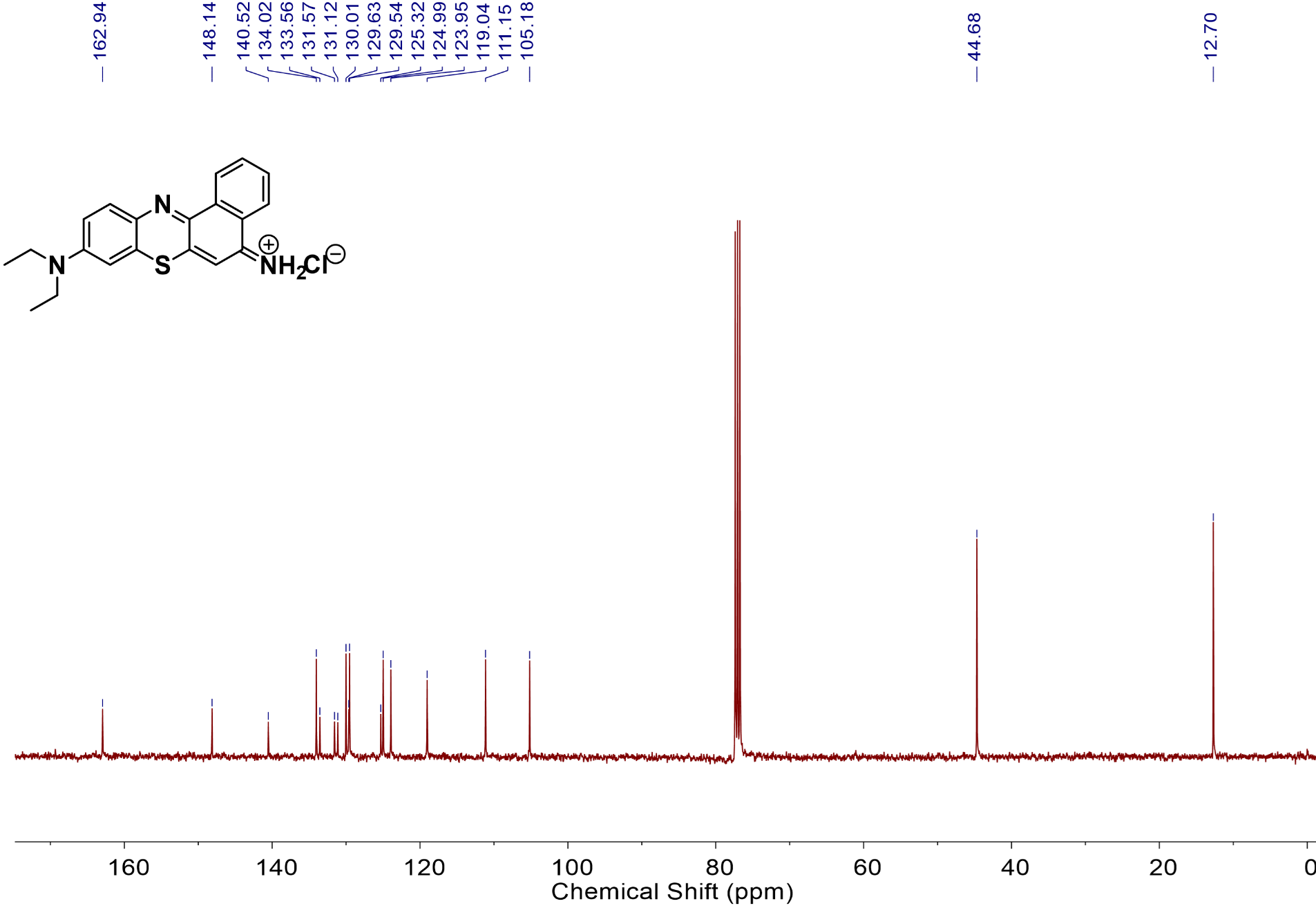


**Figure S7.** ^13^C NMR spectrum of **EtNBS-H** in CDCl_3_.


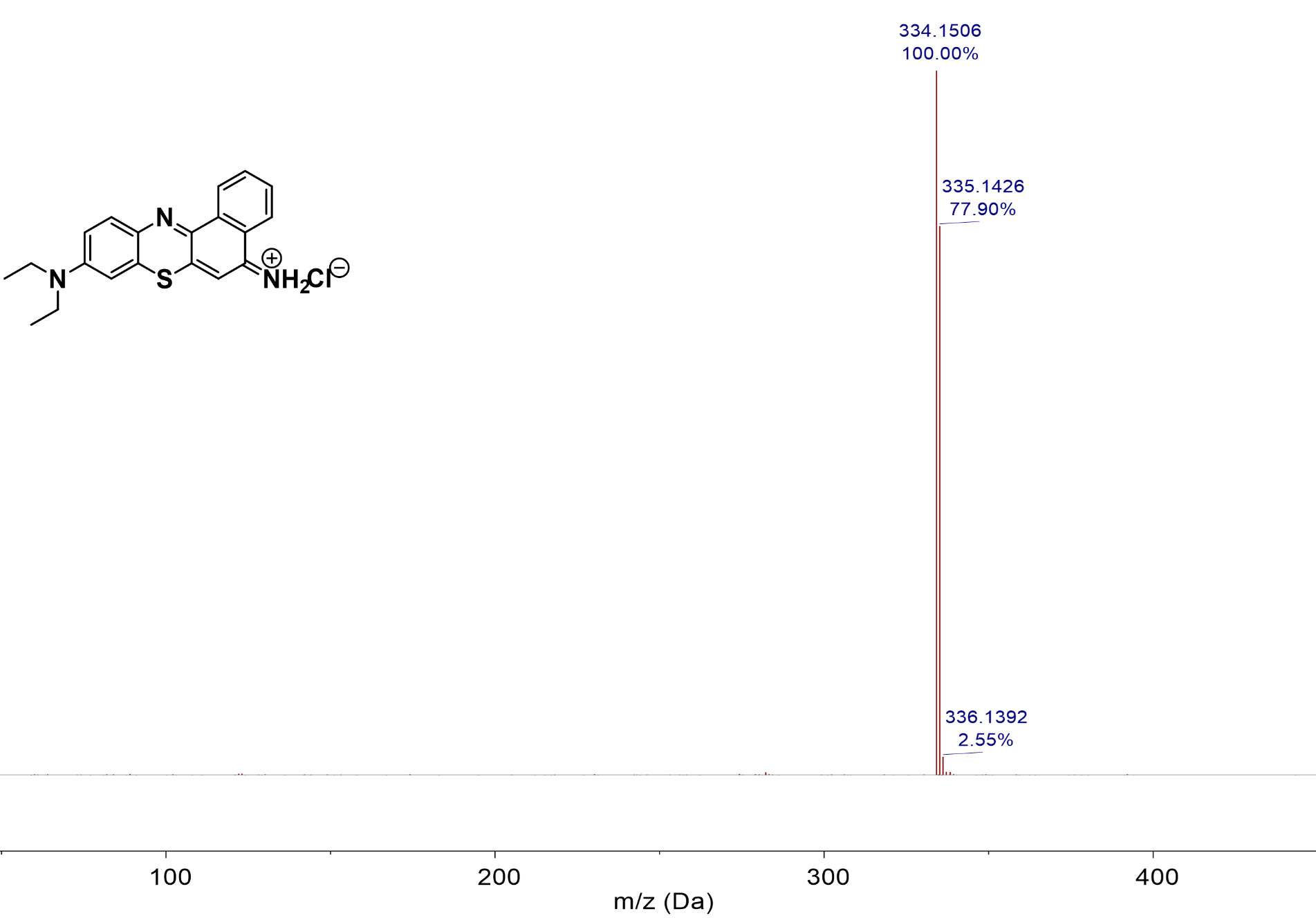


**Figure S8.** HR-MS spectrum of **EtNBS-H**.


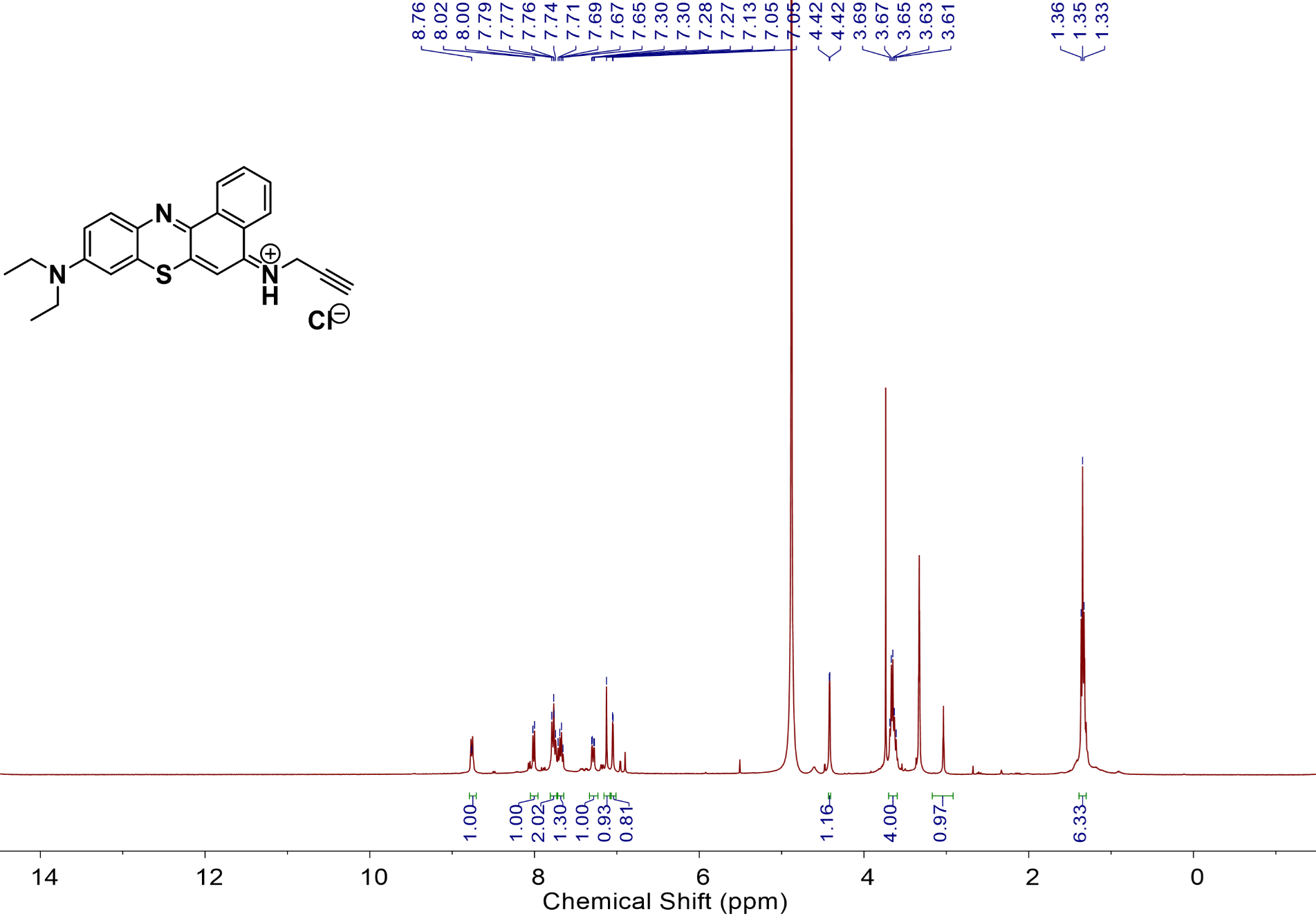


**Figure S9.** ^1^H NMR spectrum of **EtNBS-Alk** in Methanol-*d_4_*.


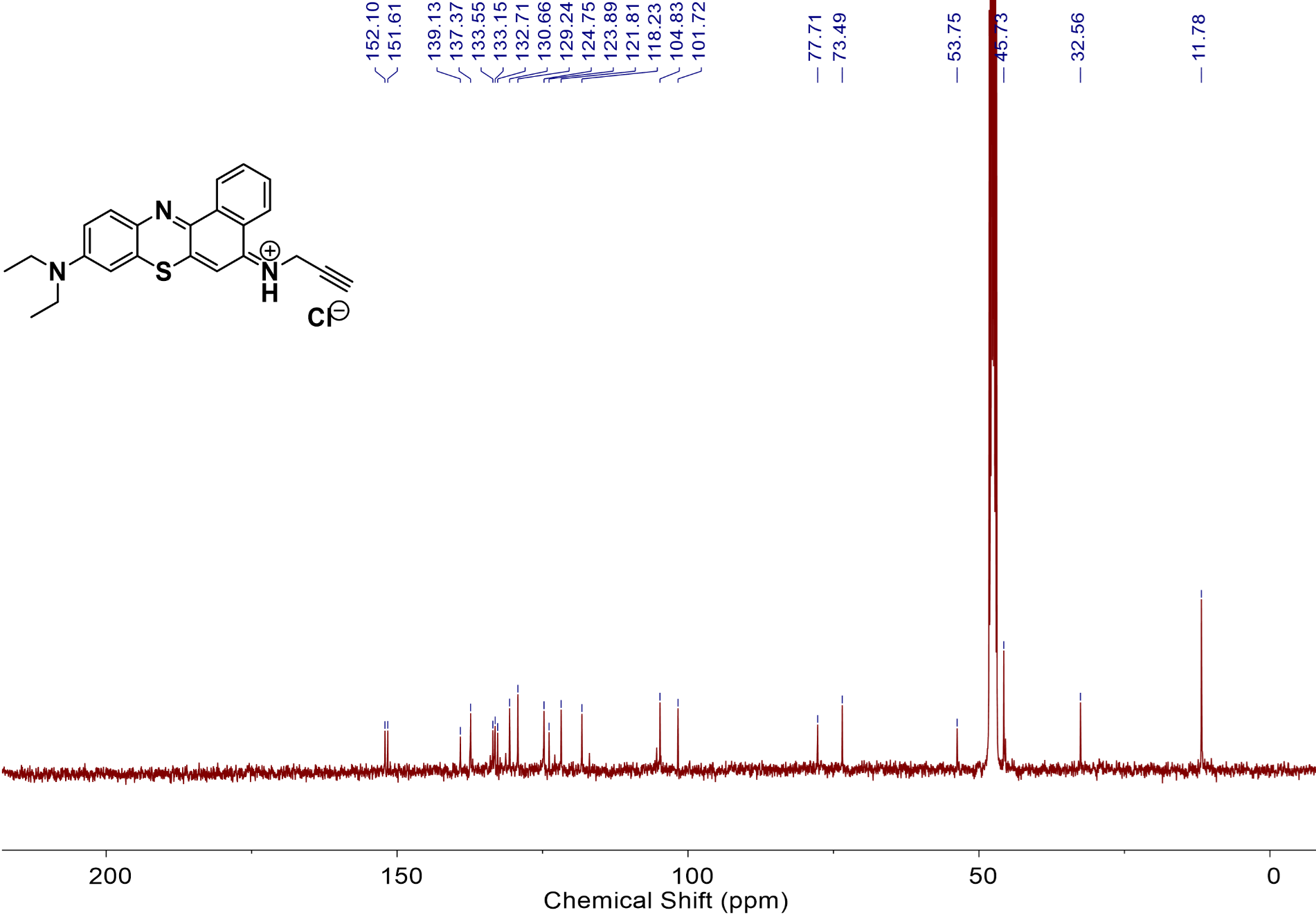


**Figure S10.** ^13^C NMR spectrum of **EtNBS-Alk** in Methanol-*d_4_*.


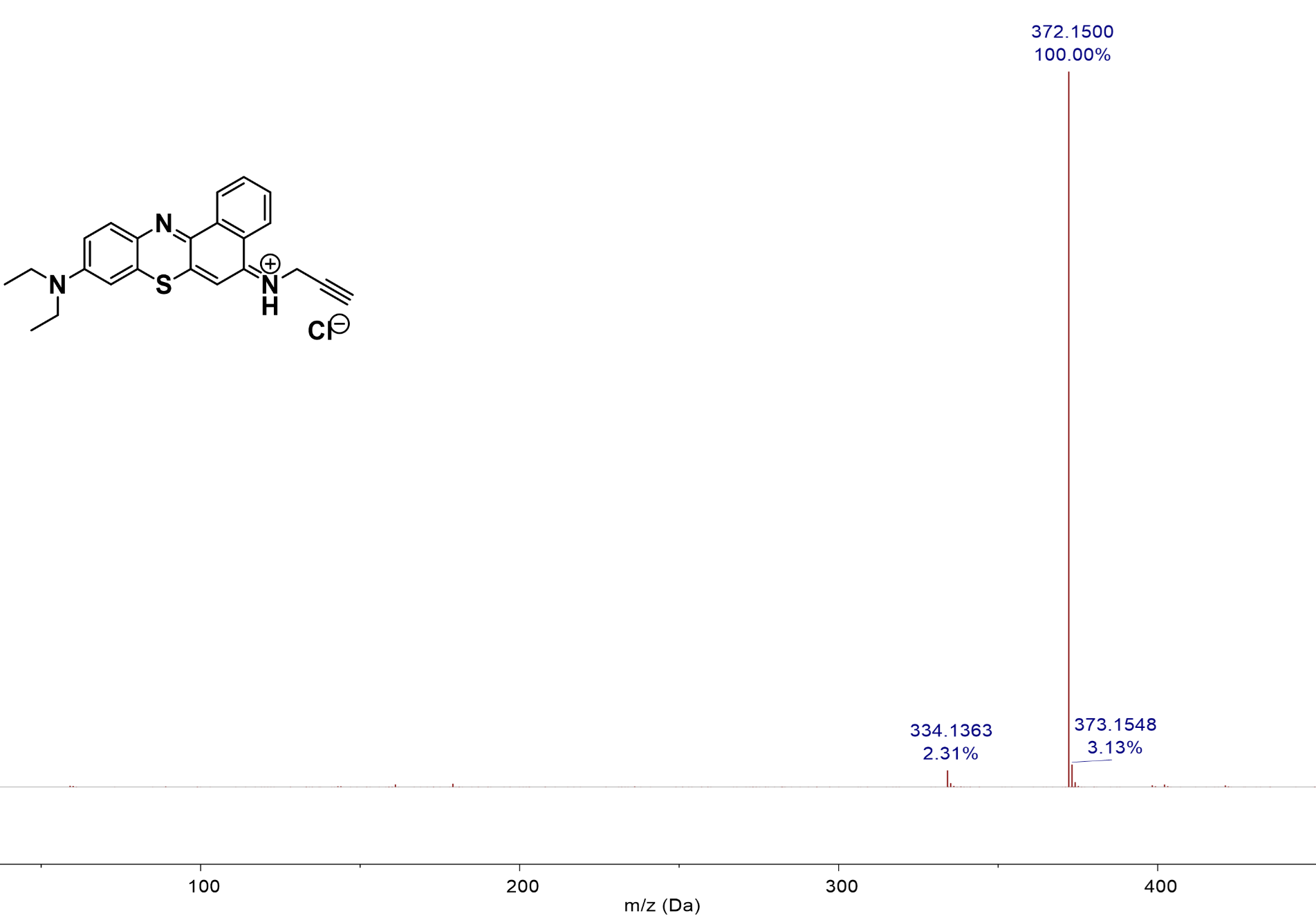


**Figure S11.** HRMS spectrum of **EtNBS-Alk**.


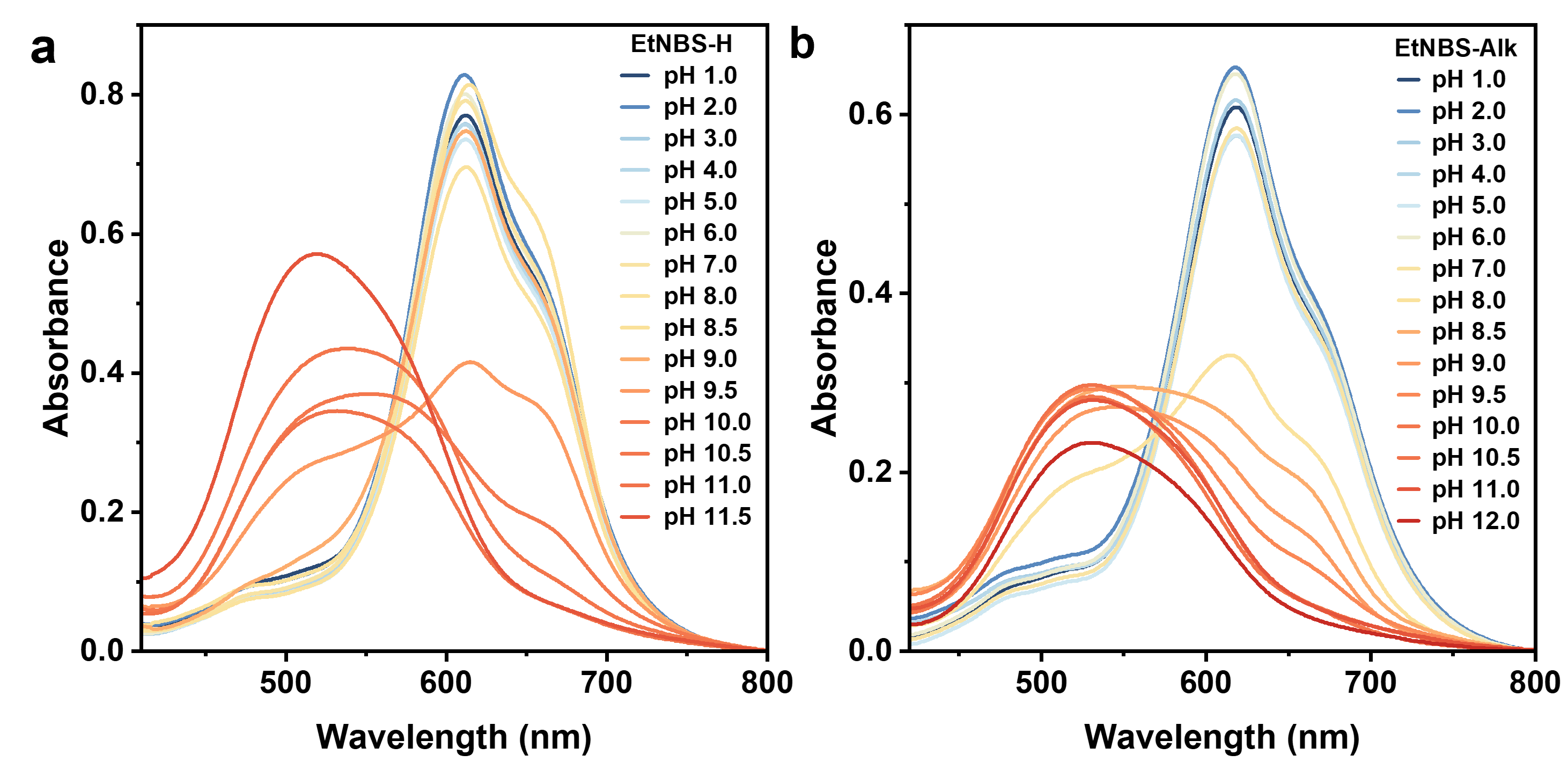


**Figure S12.** Absorbance spectra of (a) **EtNBS-H** (50 µM) and (b) **EtNBS-Alk** (50 µM) in PBS buffer solutions at different pH values.


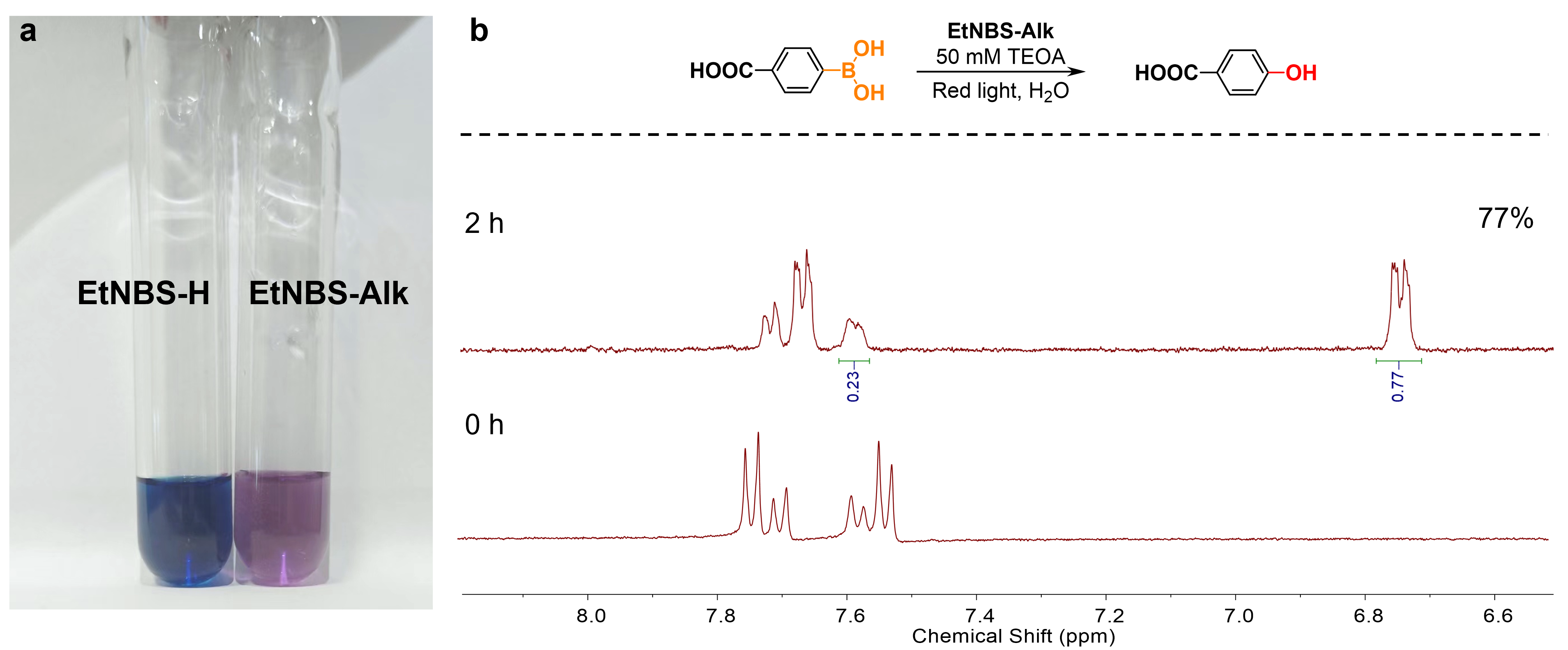


**Figure S13.** (a) The solution color changes of **EtNBS-Alk** and **EtNBS-H** under the same reaction conditions, which suggests the instability of **EtNBS-Alk**. Reaction conditions: 4-carboxyphenylboronic (5 mM), **EtNBS-H** or **EtNBS-Alk** (2 mol%), and TEOA (50 mM) in H_2_O (2 mL). (b) ^1^H NMR spectra of the products resulting from the photocatalytic oxidative hydroxylation of 4-carboxyphenylboronic in D_2_O with **EtNBS-Alk** as the photocatalyst.


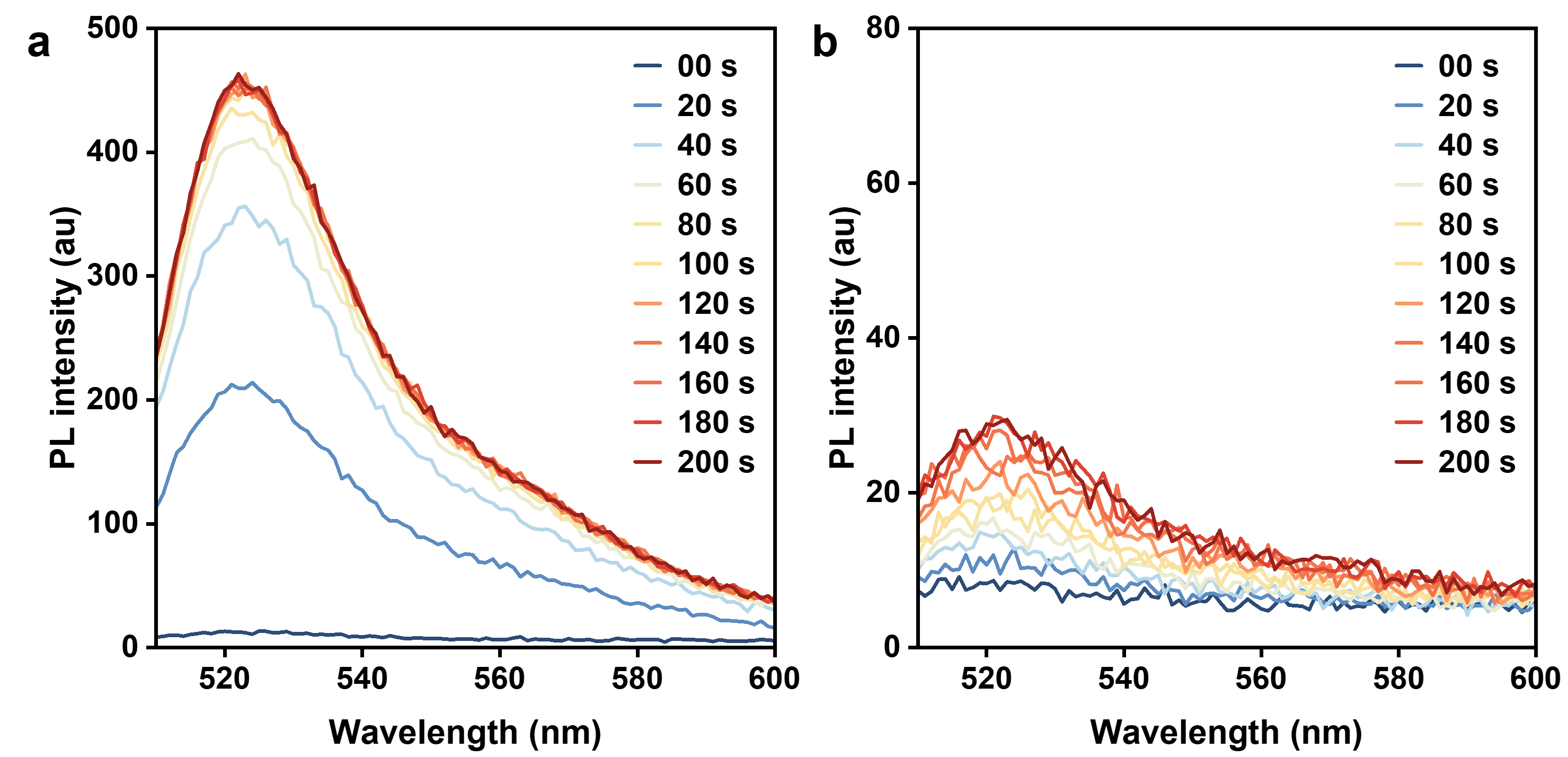


**Figure S14.** PL spectra of DCFH in the (a) presence and (b) absence of **EtNBS-H** with Xenon lamp (15 mW·cm^-2^) irradiation for different times (DCFH: 1 μM; **EtNBS-H**: 5 μM).


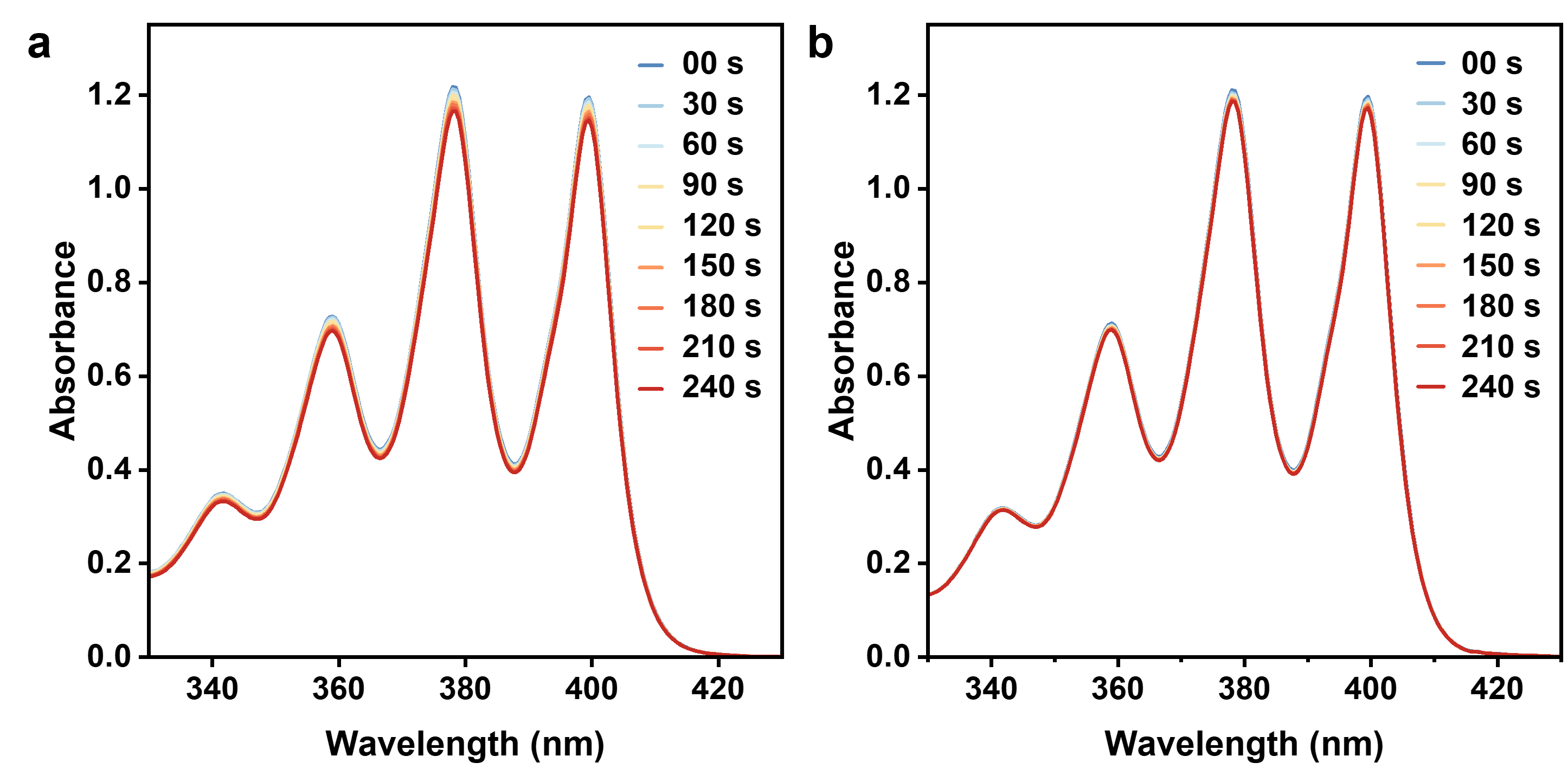


**Figure S15.** Absorption spectra of ABDA in the (a) presence and (b) absence of **EtNBS-H** with red light (45.6 mW·cm^-2^) irradiation for different times (ABDA: 100 μM; **EtNBS-H**: 5 μM).


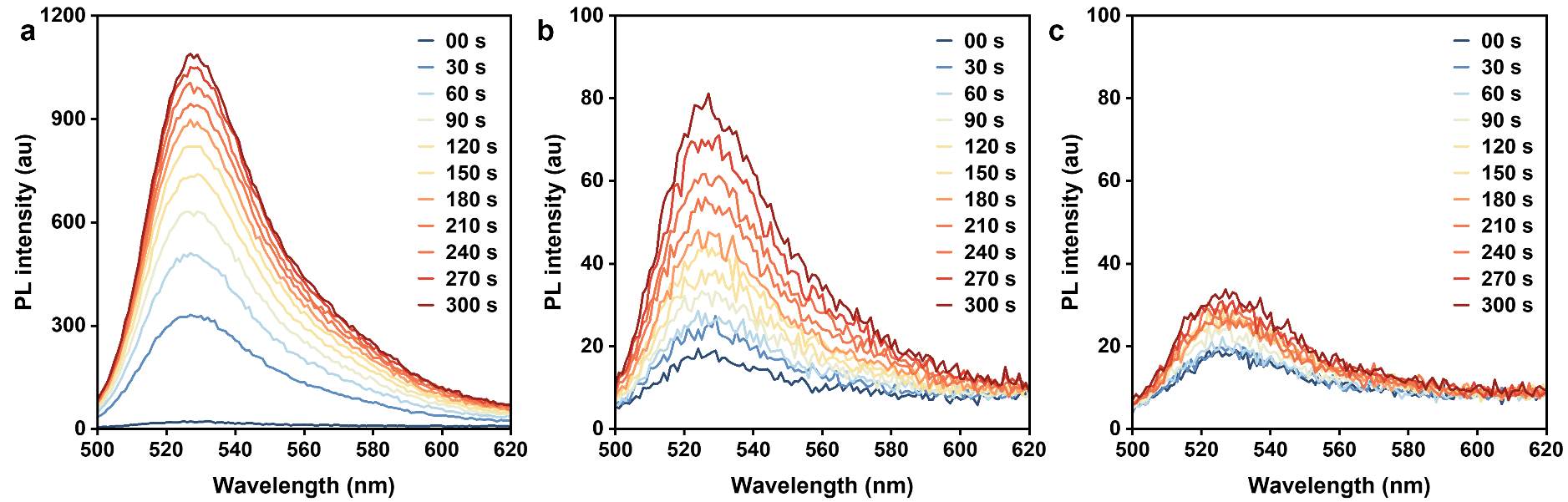


**Figure S16.** PL spectra of DHR123 solutions in the presence of (a) **EtNBS-H**, (b) blank, and (c) **EtNBS-H** with Vc upon red light (45.6 mW·cm^-2^) irradiation for different times (DHR123: 10 μM; Vc: 100 μM; **EtNBS-H**: 5 μM).

**Table S1.** The singlet and triplet excited states energy of **EtNBS-H** reveal by TD-DFT calculation.

|  | **S_1_** | **S_2_** | **S_3_** | **T_1_** | **T_2_** | **T_3_** |
| --- | --- | --- | --- | --- | --- | --- |
| **EtNBS-H** | 2.3904 eV | 3.0918 eV | 3.3822 eV | 1.1339 eV | 2.1626 eV | 2.9306 eV |

**
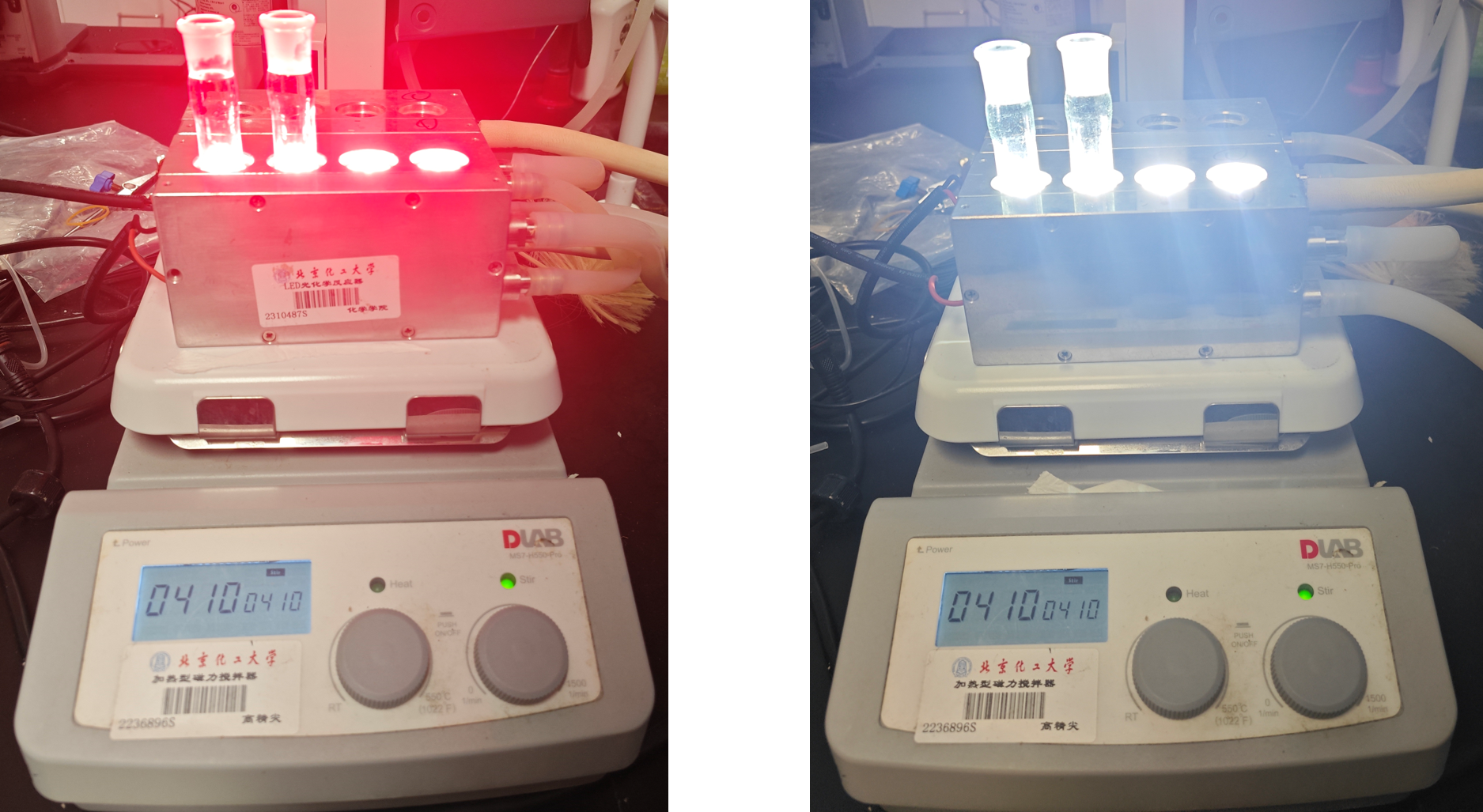
**

**Figure S17.** Reaction devices (white light and red light).

**
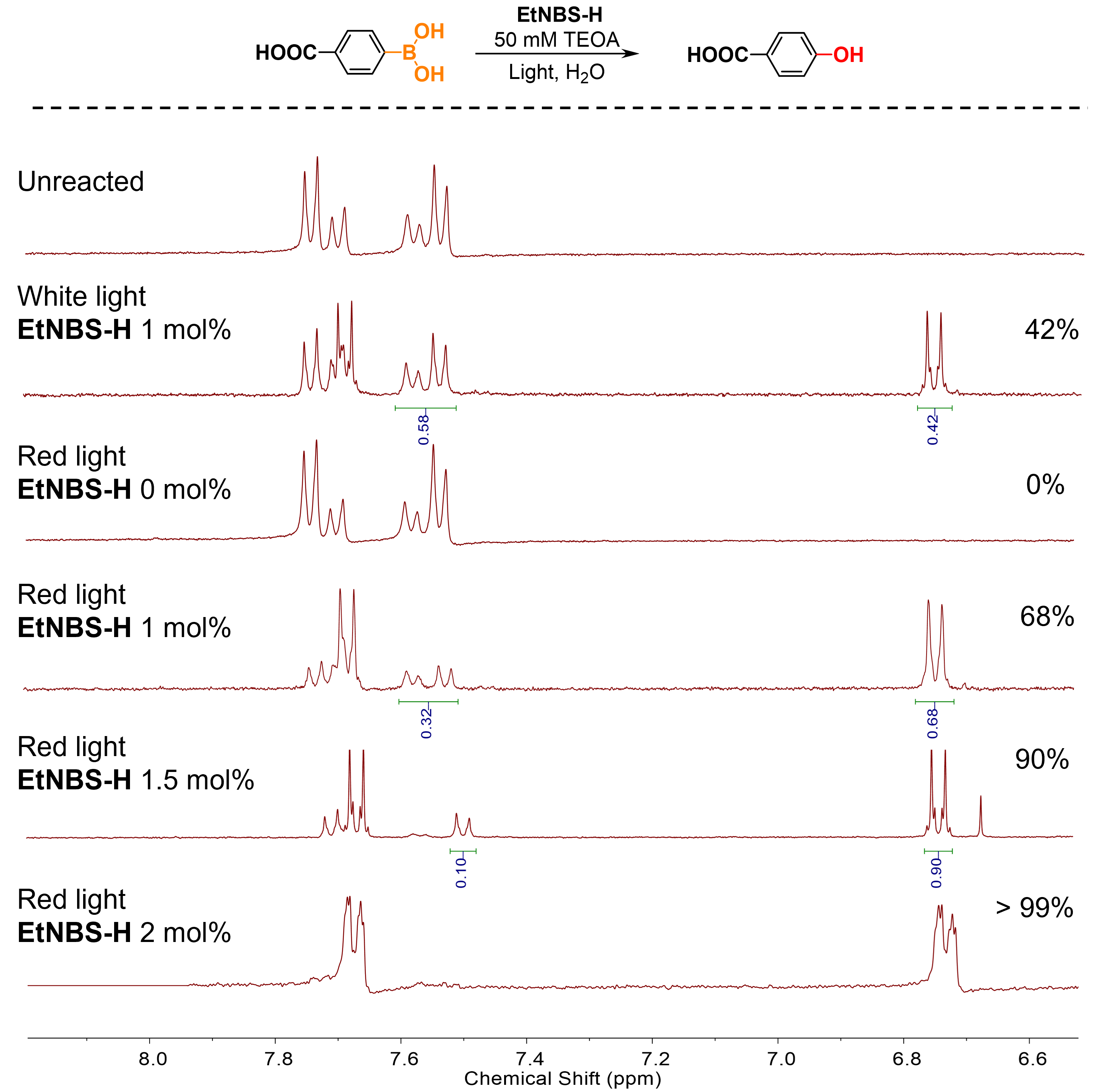
**

**Figure S18.** ^1^H NMR spectra of the products resulting from the photocatalytic oxidative hydroxylation of 4-carboxyphenylboronic in D_2_O, under different light irradiation and different concentrations of **EtNBS-H**.


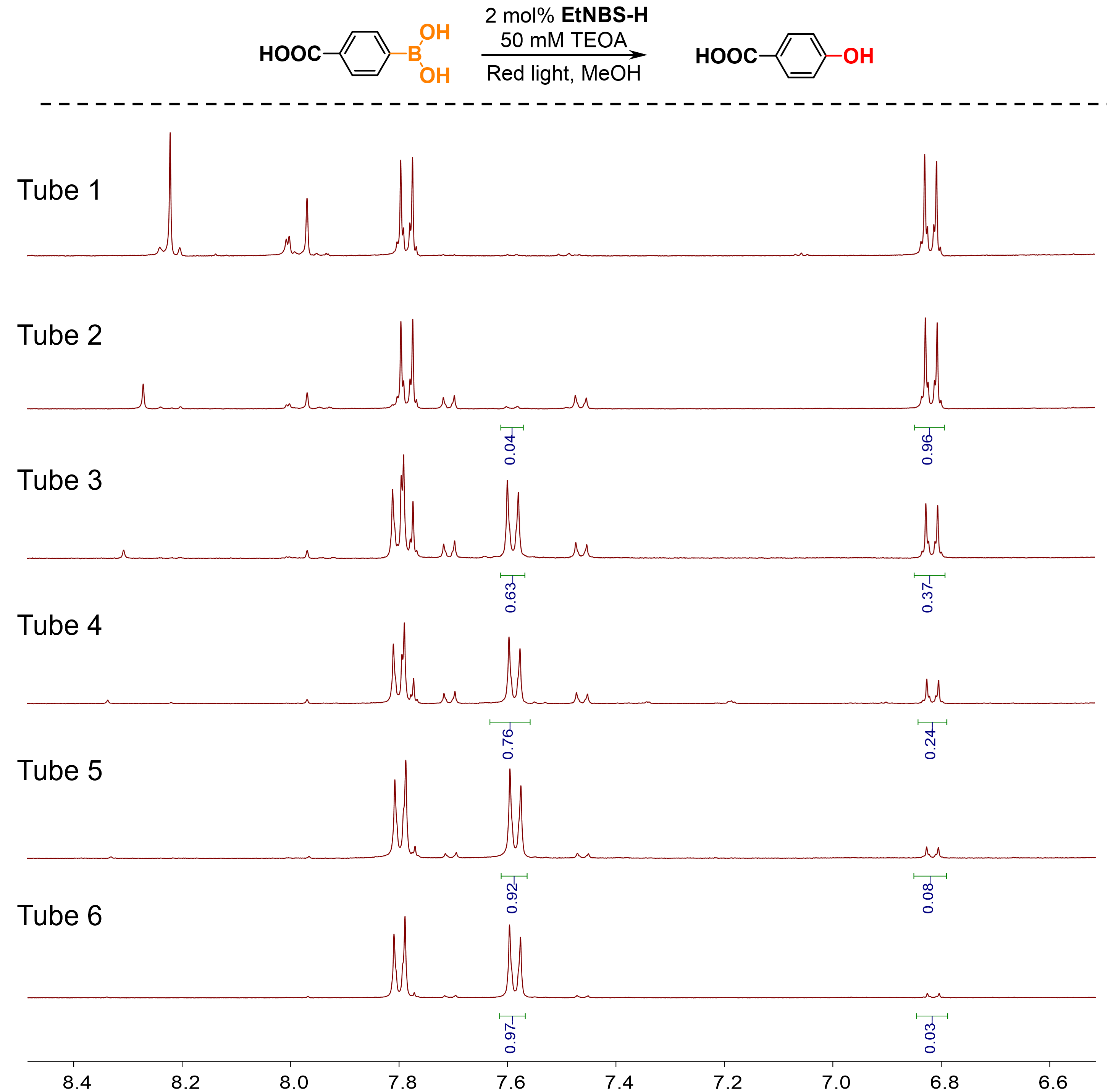


**Figure S19.** ^1^H NMR spectra of the products resulting from the photocatalytic oxidative hydroxylation of 4-carboxyphenylboronic in DMSO-*d_6_*. Reaction conditions: 4-carboxyphenylboronic (5 mM), **EtNBS-H** (2 mol%), and TEOA (50 mM) in MeOH (2 mL), irradiated with red light for 2 h at room temperature under ambient atmosphere.


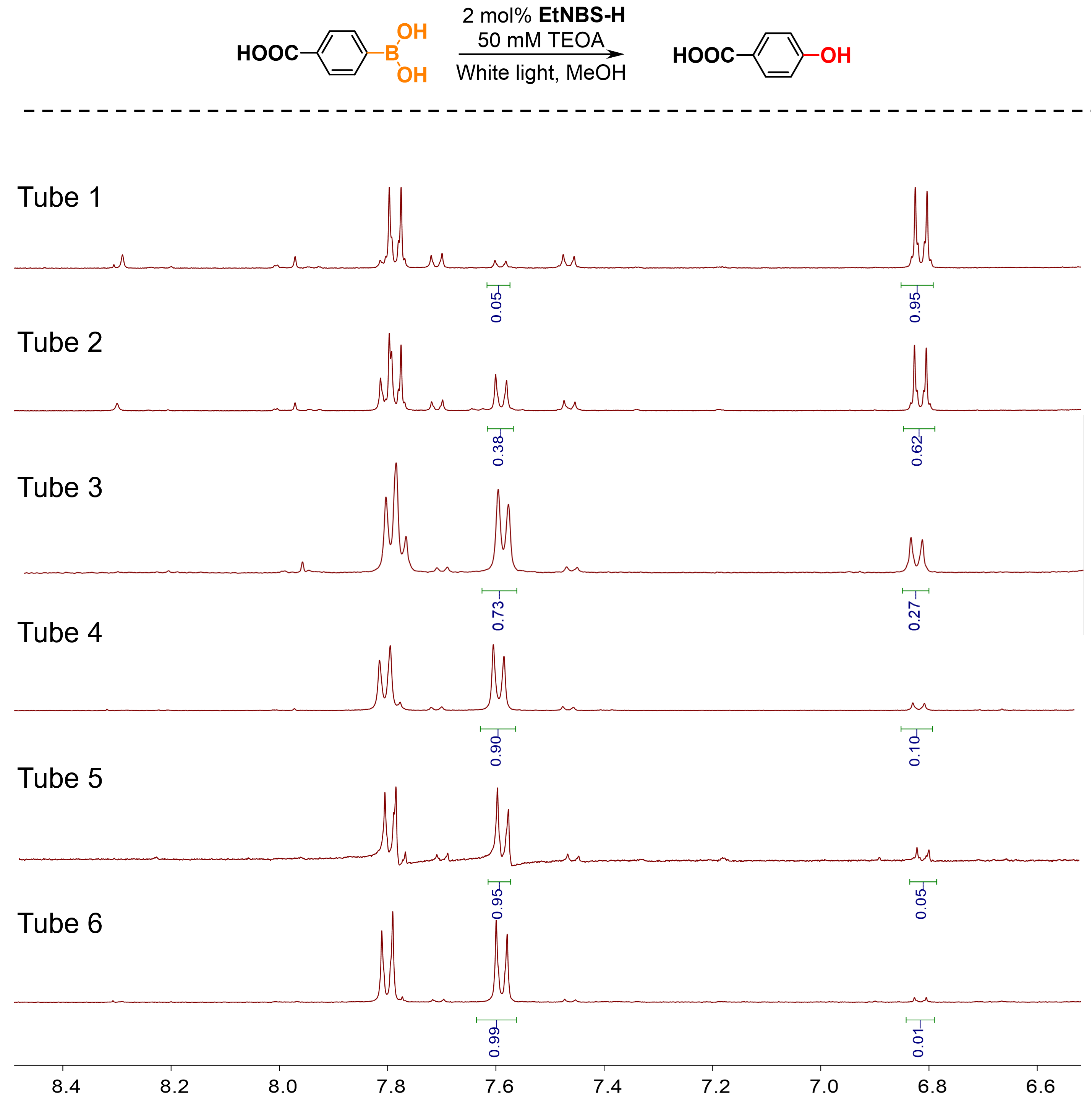


**Figure S20.** ^1^H NMR spectra of the products resulting from the photocatalytic oxidative hydroxylation of 4-carboxyphenylboronic in DMSO-*d_6_*. Reaction conditions: 4-carboxyphenylboronic (5 mM), **EtNBS-H** (2 mol%), and TEOA (50 mM) in MeOH (2 mL), irradiated with white light for 2 h at room temperature under ambient atmosphere.

**Table S2.** Light power density of different reaction tubes.

| Light | Light power density (mW·cm^-2^) | | | | | |
| --- | --- | --- | --- | --- | --- | --- |
|  | Tube 1 | Tube 2 | Tube 3 | Tube 4 | Tube 5 | Tube 6 |
| White light | 80.5 | 42.7 | 26.2 | 17.2 | 11.3 | 8.4 |
| Red light | 45.6 | 31.2 | 14.5 | 8.9 | 6.3 | 5.8 |


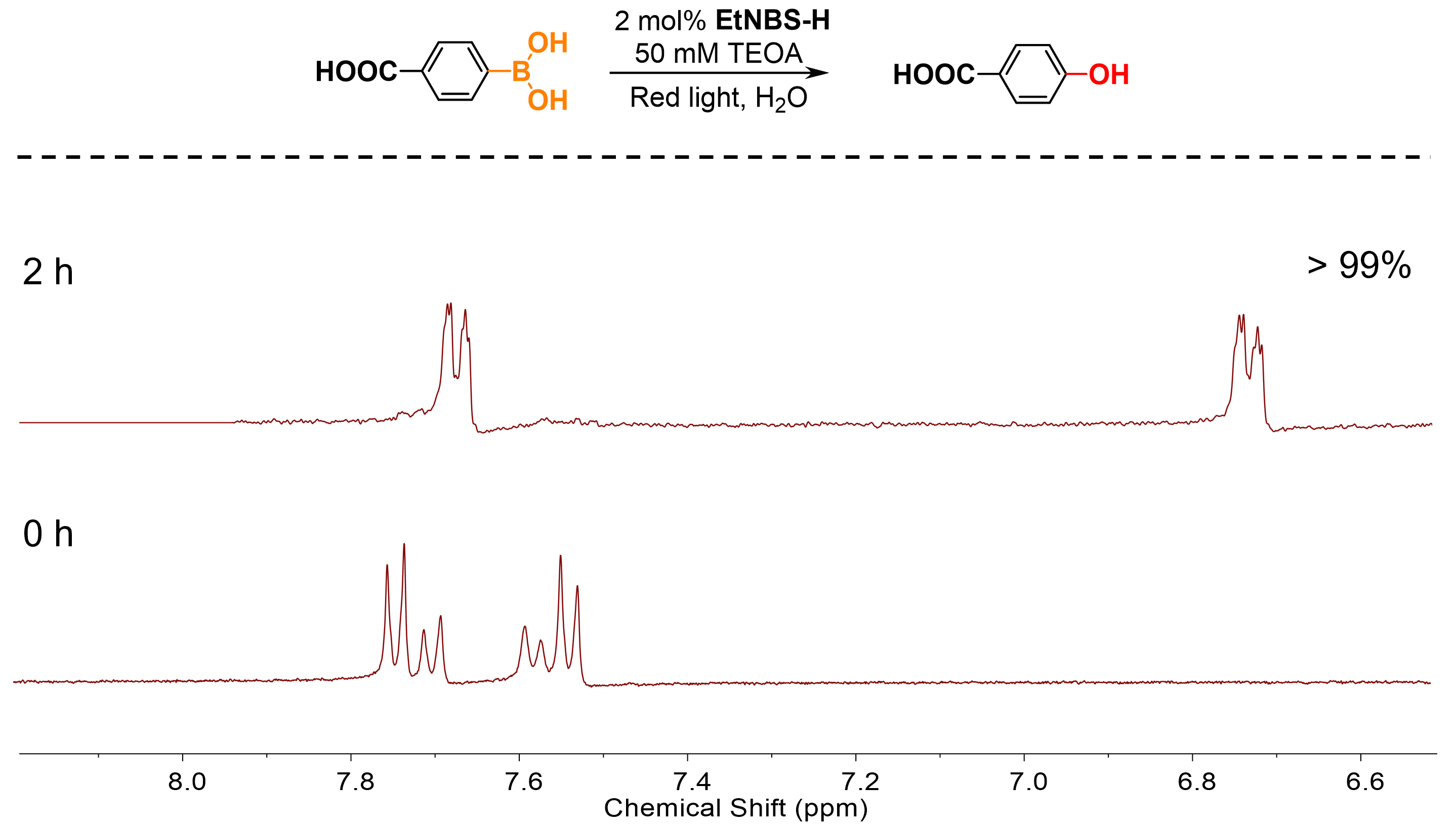


**Figure S21.** ^1^H NMR spectra of the products resulting from the photocatalytic oxidative hydroxylation of 4-carboxyphenylboronic in D_2_O.


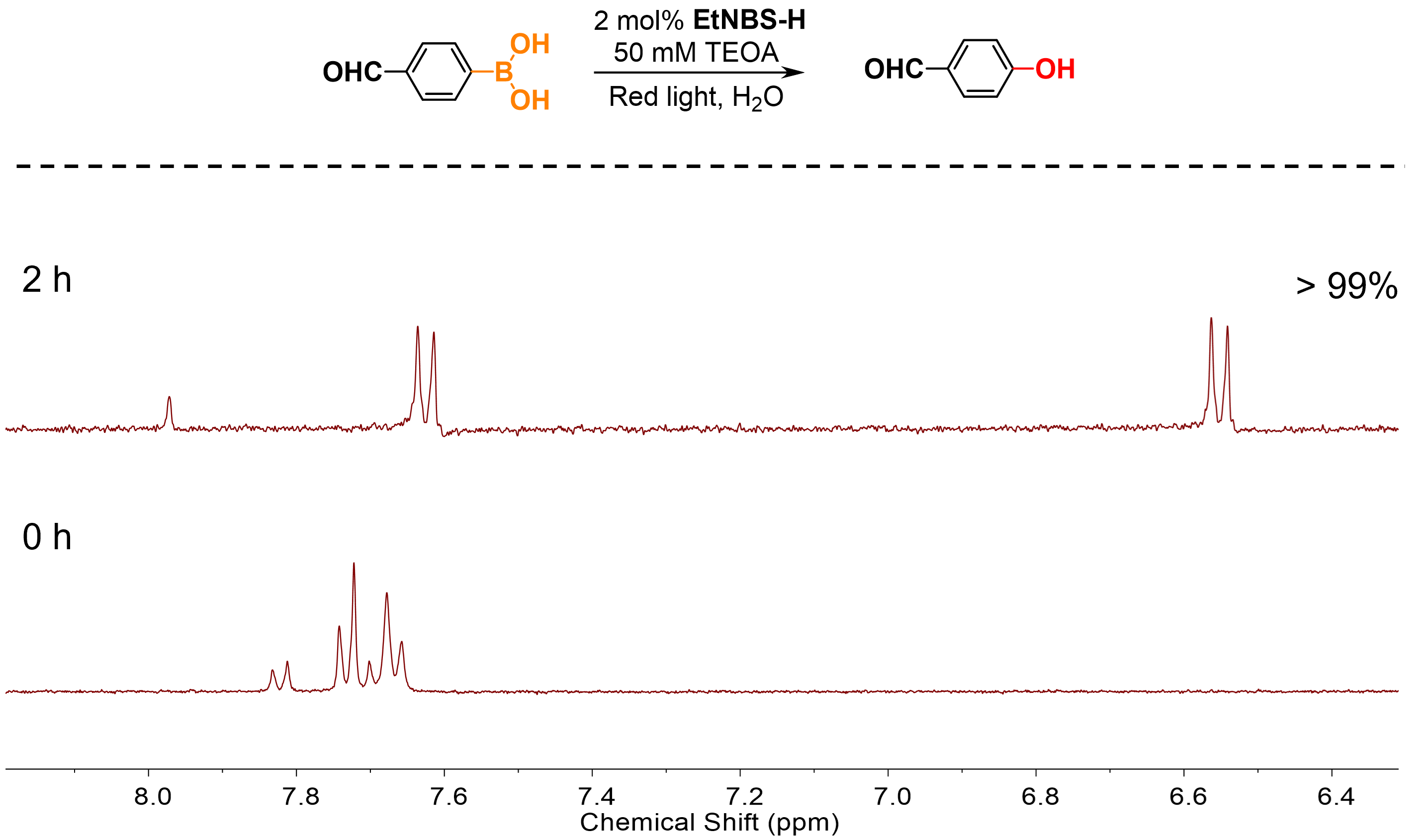


**Figure S22.** ^1^H NMR spectra of the products resulting from the photocatalytic oxidative hydroxylation of 4-formylphenylboronic acid in D_2_O.


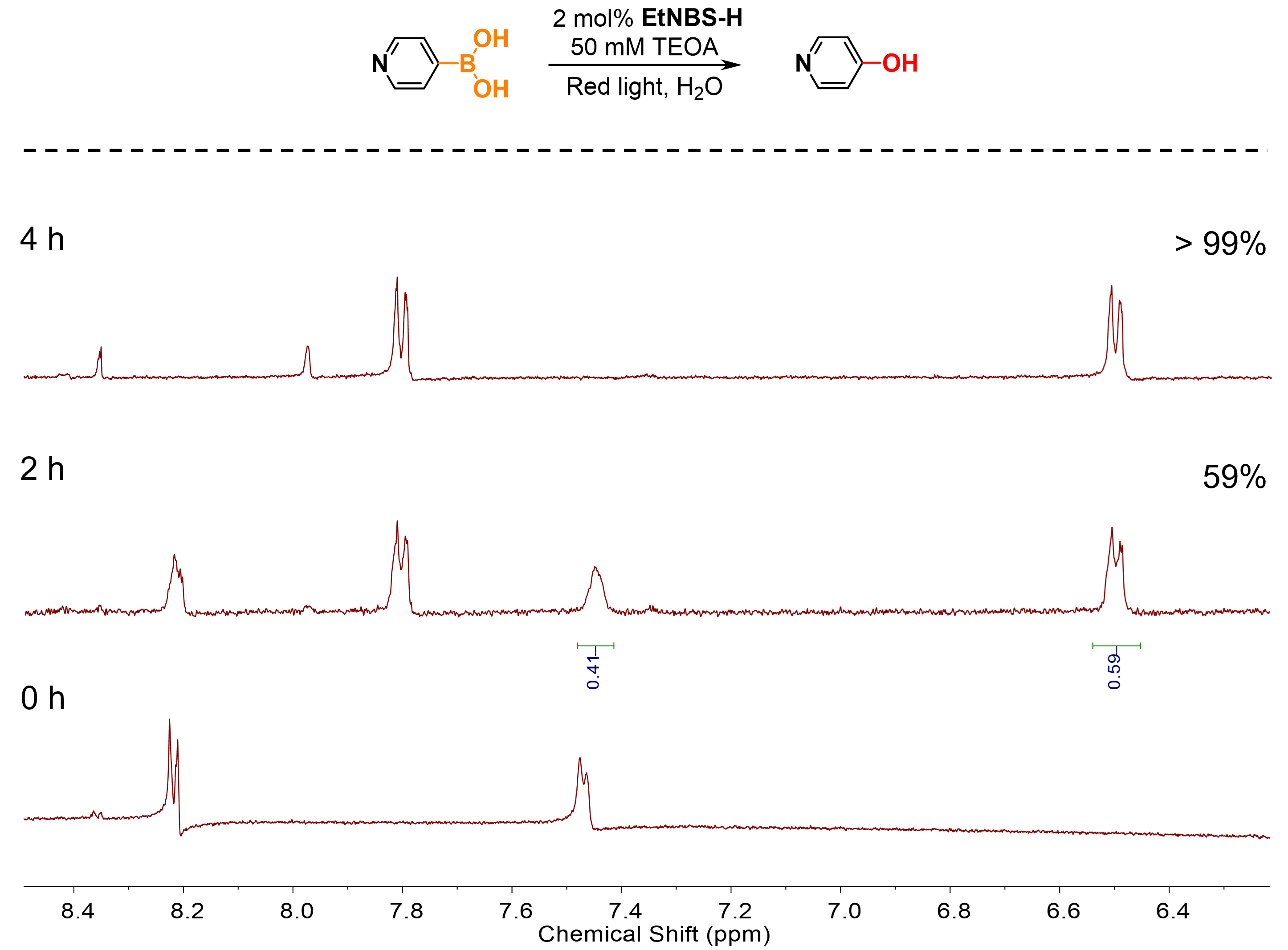


**Figure S23.** ^1^H NMR spectra of the products resulting from the photocatalytic oxidative hydroxylation of 4-boronopyridine in D_2_O.


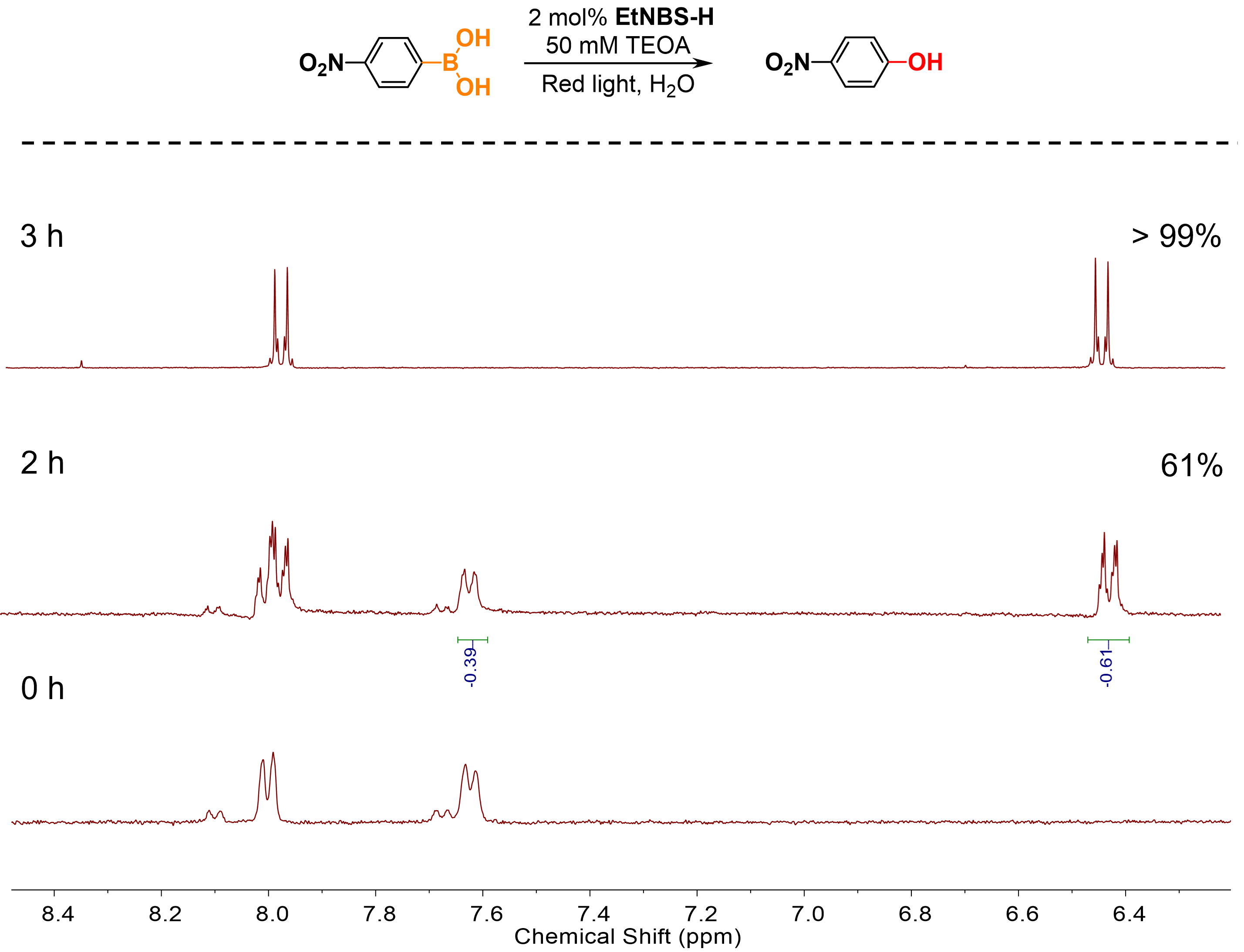


**Figure S24.** ^1^H NMR spectra of the products resulting from the photocatalytic oxidative hydroxylation of 4-nitrobenzeneboronic acid in D_2_O.


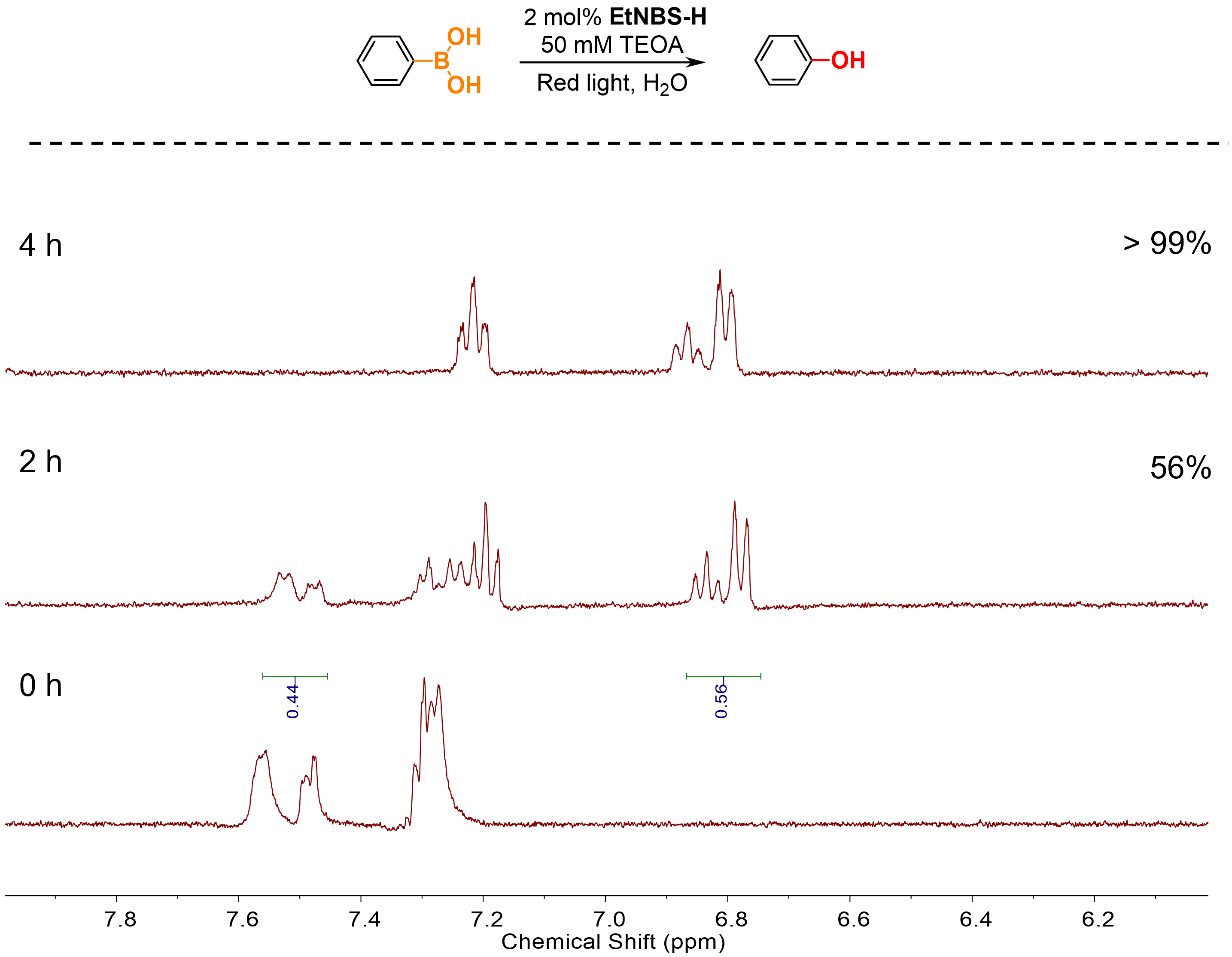


**Figure S25.** ^1^H NMR spectra of the products obtained from the photocatalytic oxidative hydroxylation of phenylboronic acid in D_2_O.


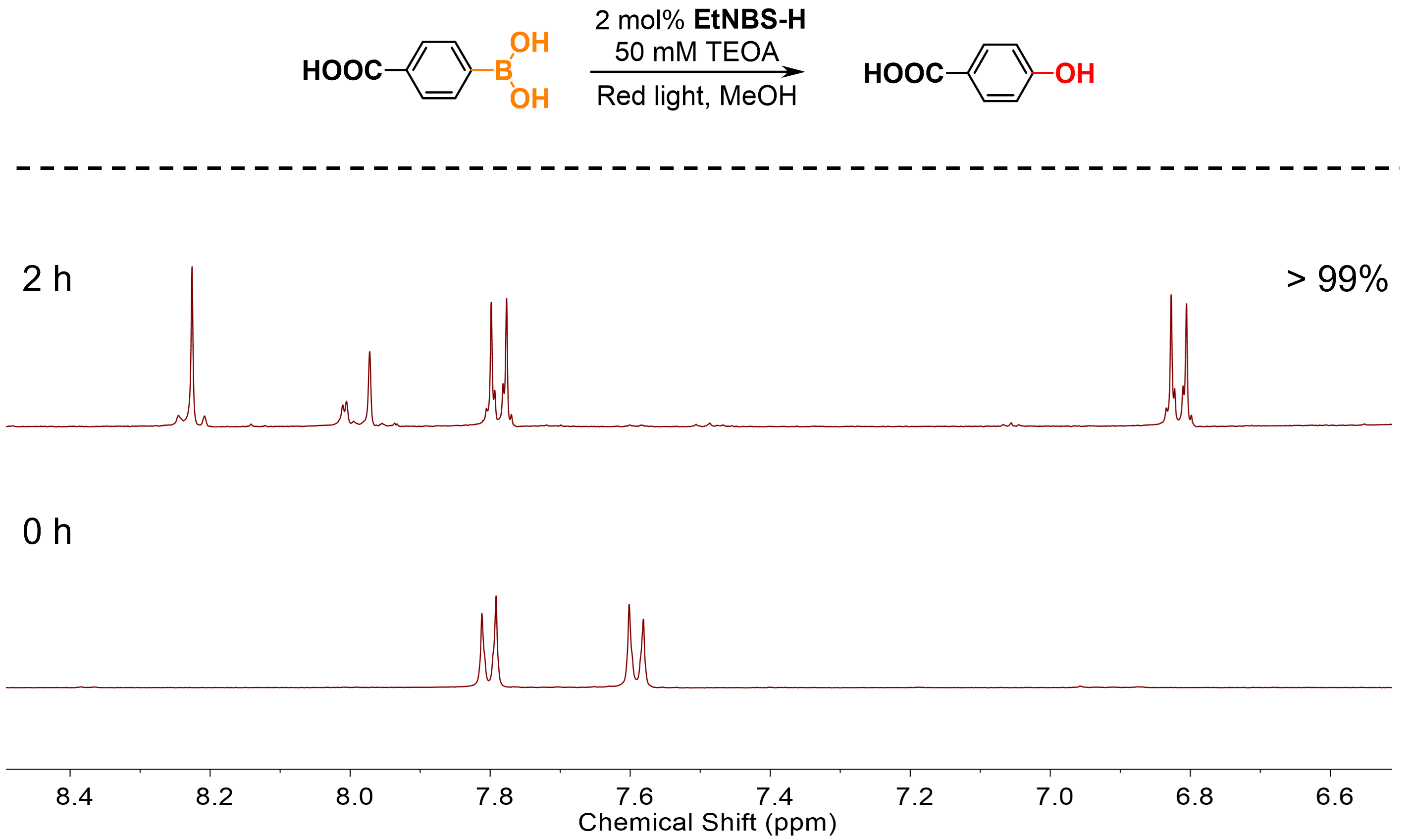


**Figure S26.** ^1^H NMR spectra of the products obtained from the photocatalytic oxidative hydroxylation of 4-carboxyphenylboronic in DMSO-*d_6_*.

**
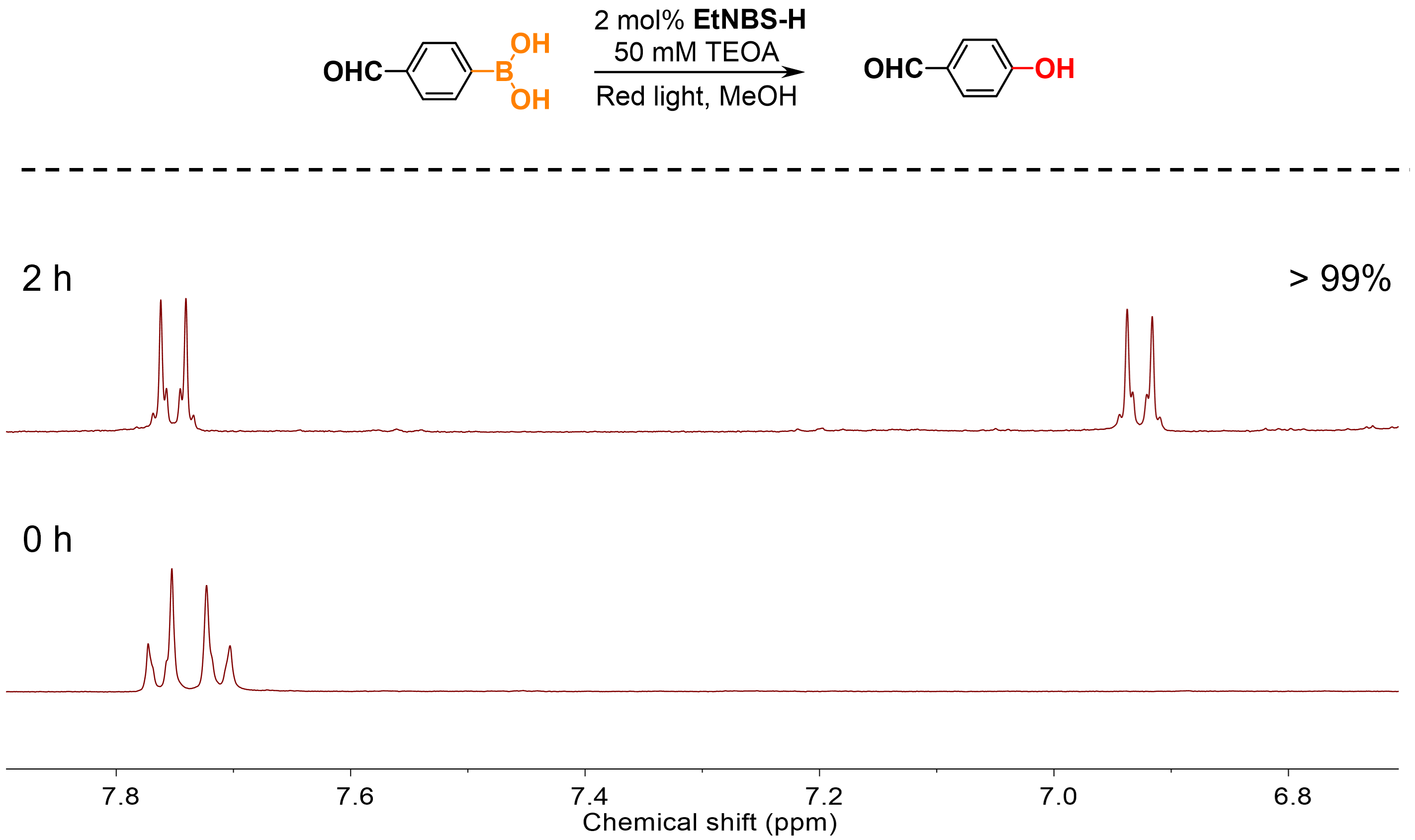
**

**Figure S27.** ^1^H NMR spectra of the products obtained from the photocatalytic oxidative hydroxylation of 4-formylphenylboronic acid in DMSO-*d_6_*.


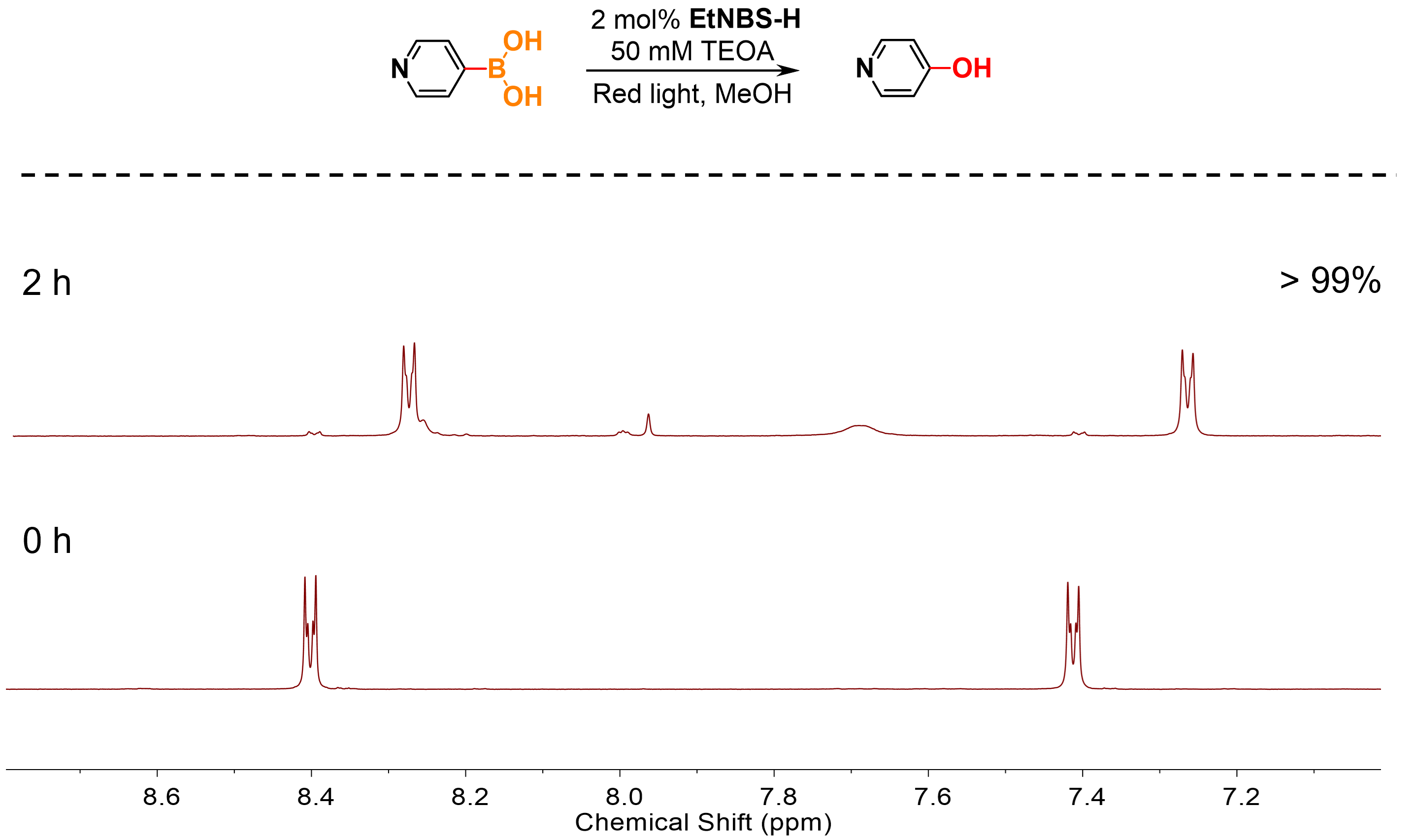


**Figure S28.** ^1^H NMR spectra of products from the photocatalytic oxidative hydroxylation of 4-boronopyridine in DMSO-*d_6_*.

**
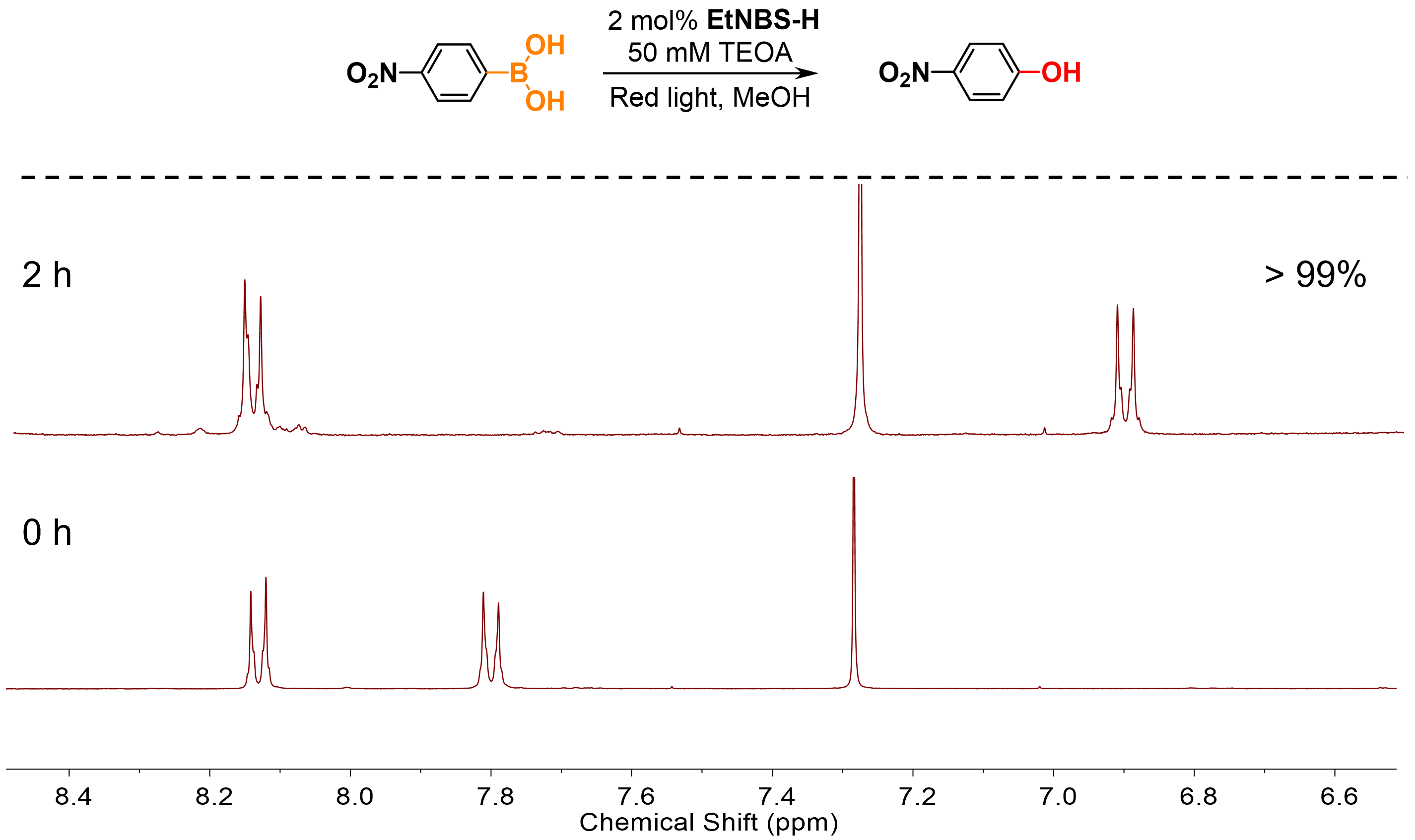
**

**Figure S29.** ^1^H NMR spectra of the products obtained from the photocatalytic oxidative hydroxylation of 4-nitrobenzeneboronic acid in CDCl_3_.


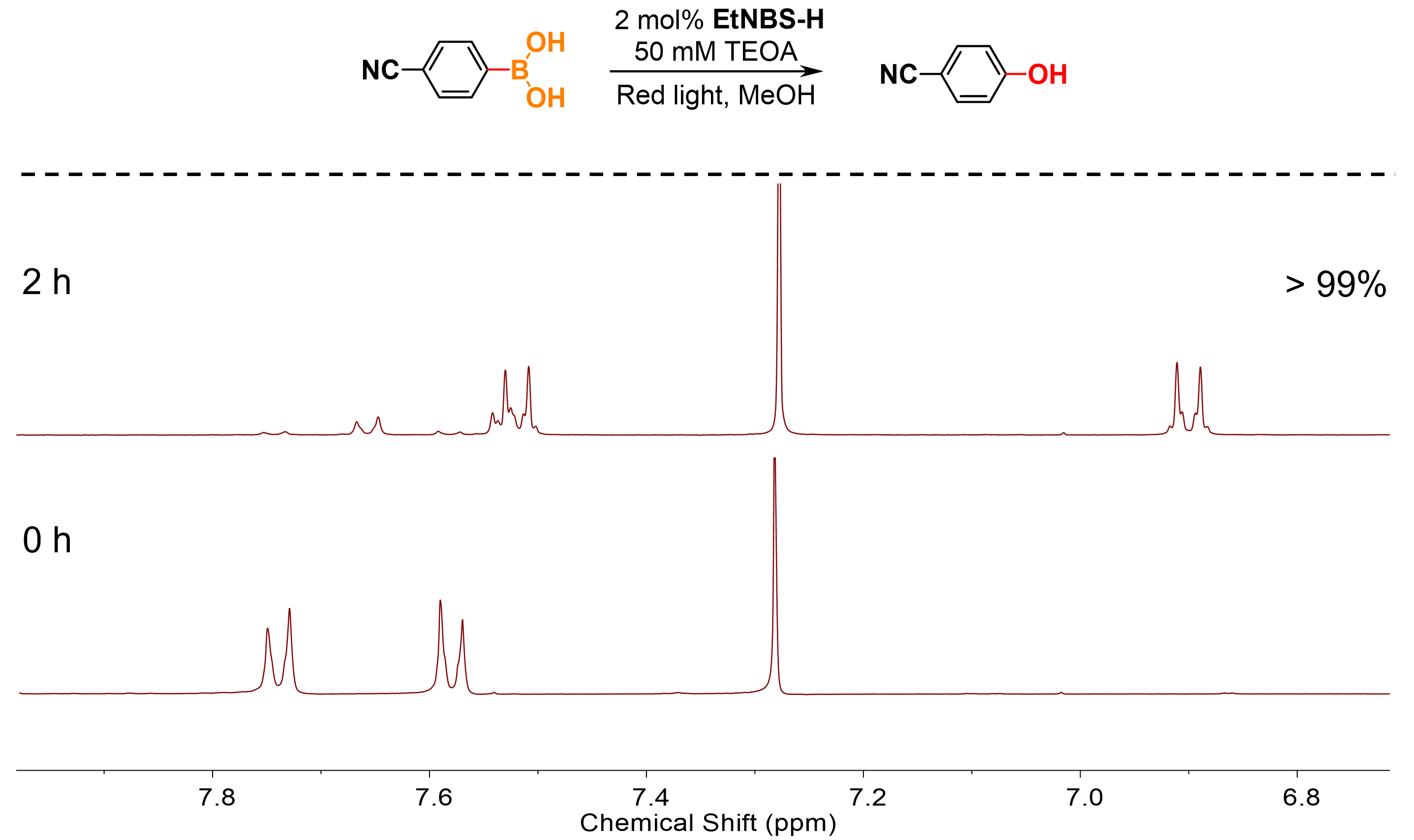


**Figure S30.** ^1^H NMR spectra of the products obtained from the photocatalytic oxidative hydroxylation of 4-cyanophenylboronic acid in CDCl_3_.

**
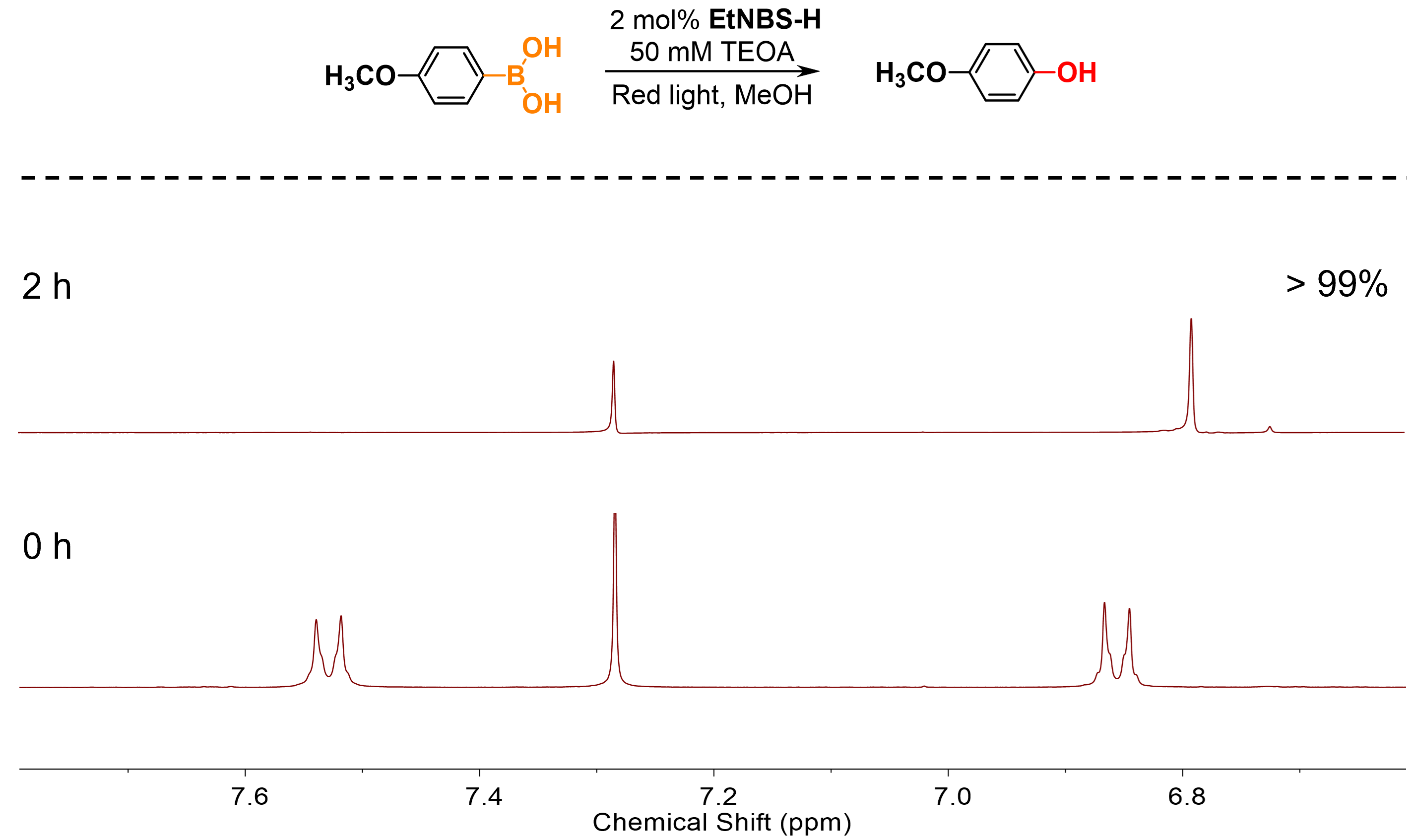
**

**Figure S31.** ^1^H NMR spectra of the products obtained from the photocatalytic oxidative hydroxylation of 4-methoxyphenylboronic acid in CDCl_3_.

**
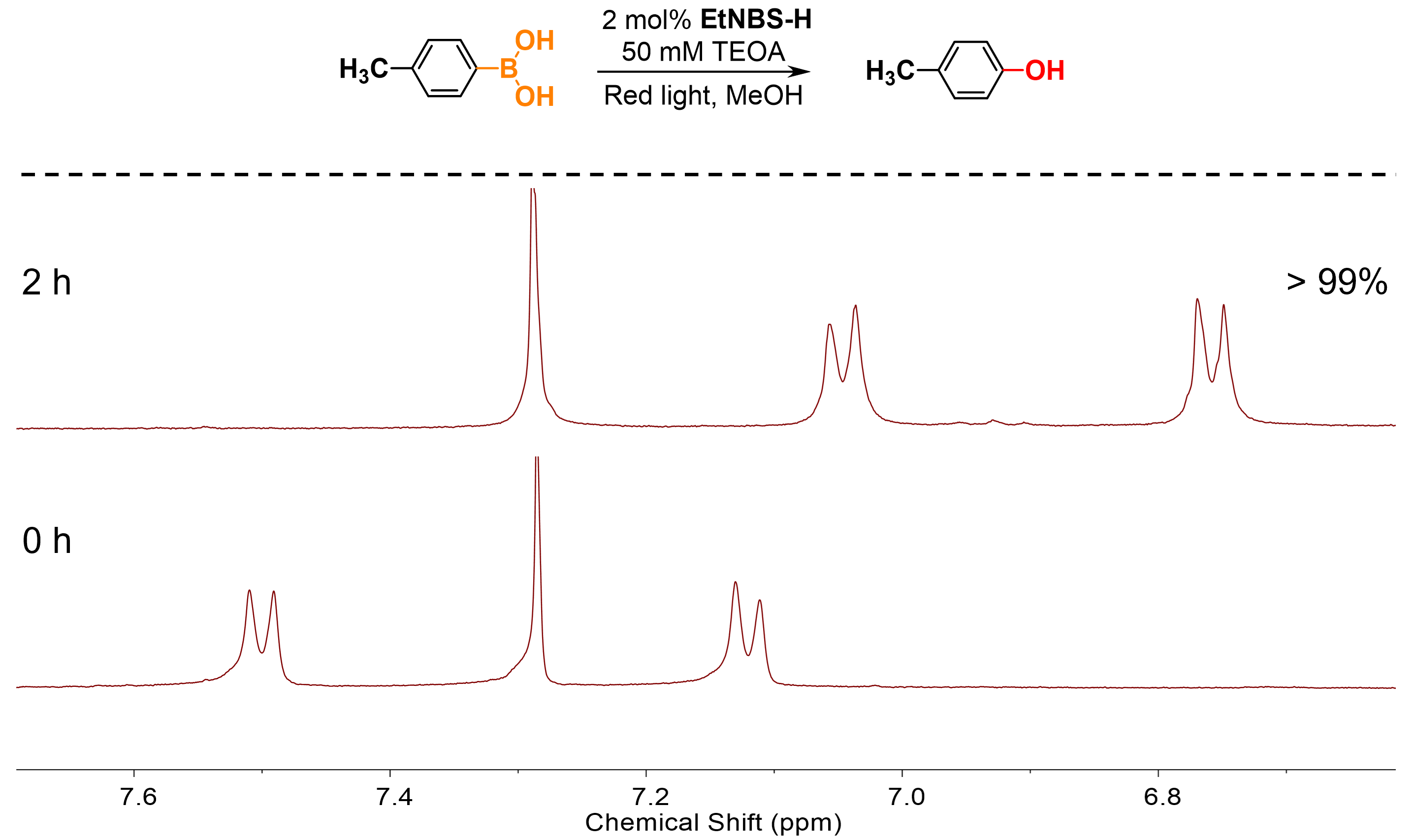
**

**Figure S32.** ^1^H NMR spectra of the products obtained from the photocatalytic oxidative hydroxylation of 4-tolylboronic acid in CDCl_3_.


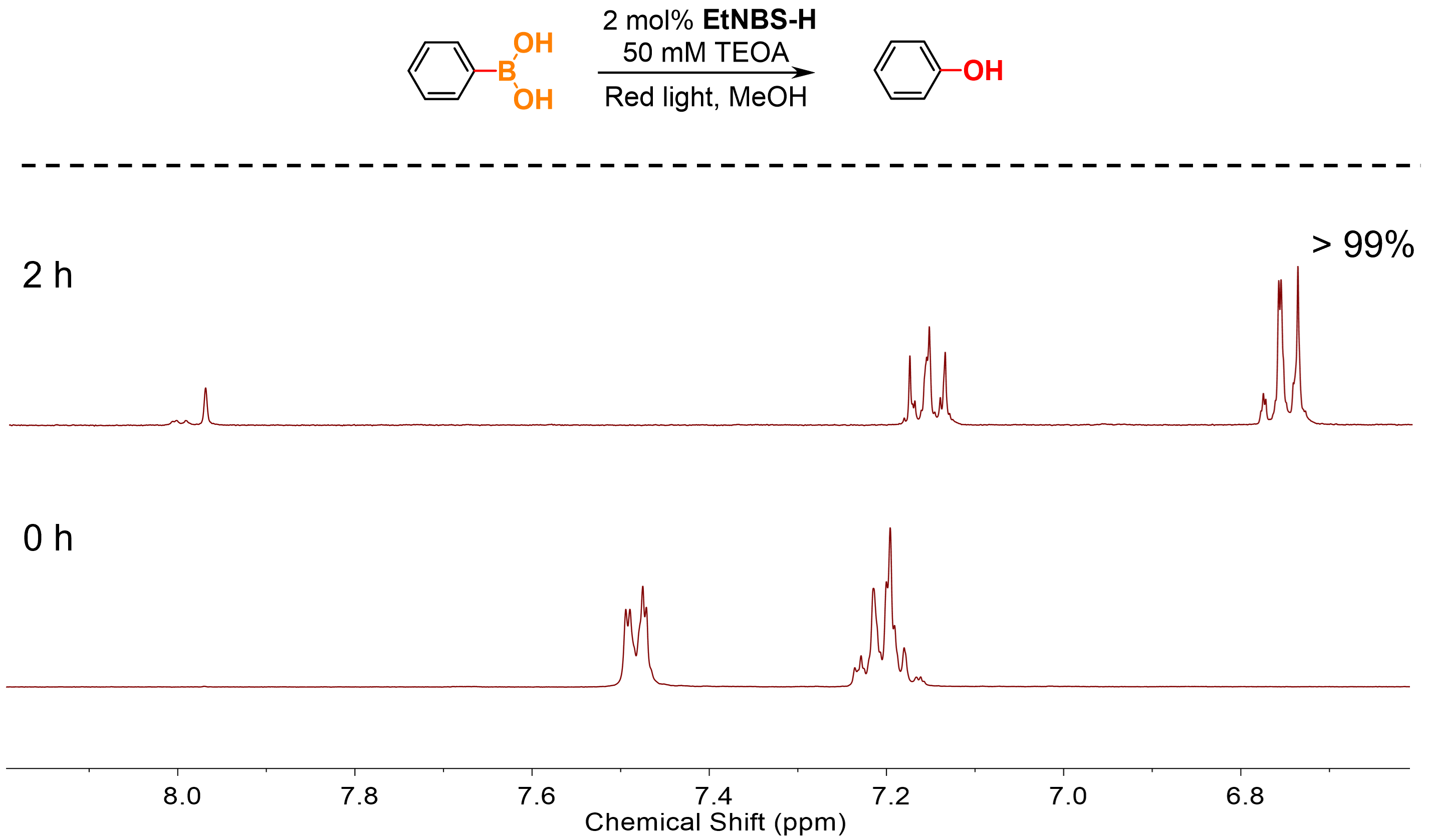


**Figure S33.** ^1^H NMR spectra of the products obtained from the photocatalytic oxidative hydroxylation of phenylboronic acid in DMSO-*d_6_*.


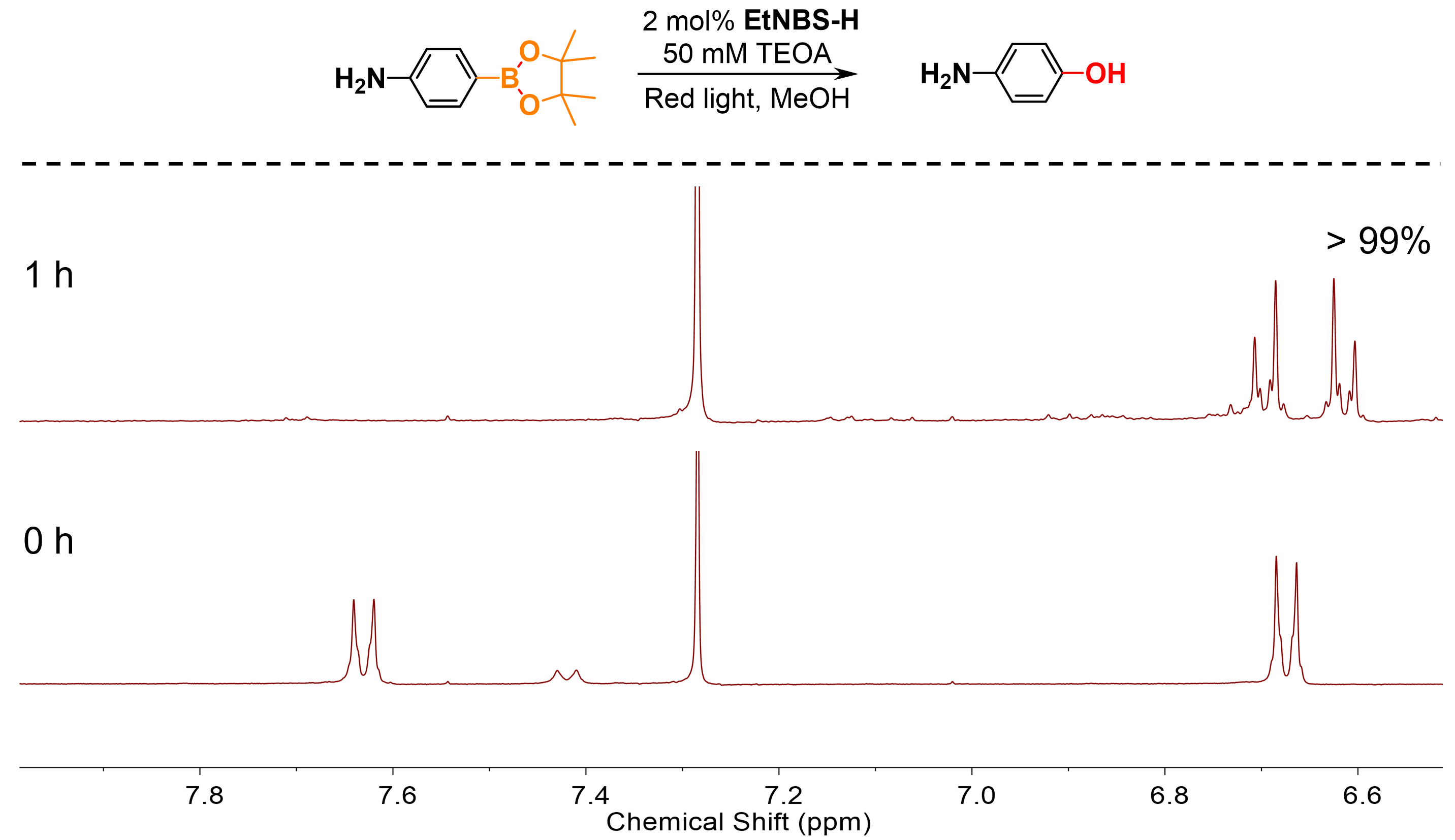


**Figure S34.** ^1^H NMR spectra of the products obtained from the photocatalytic oxidative hydroxylation of 4-aminophenylboronic acid pinacol ester in CDCl_3_.


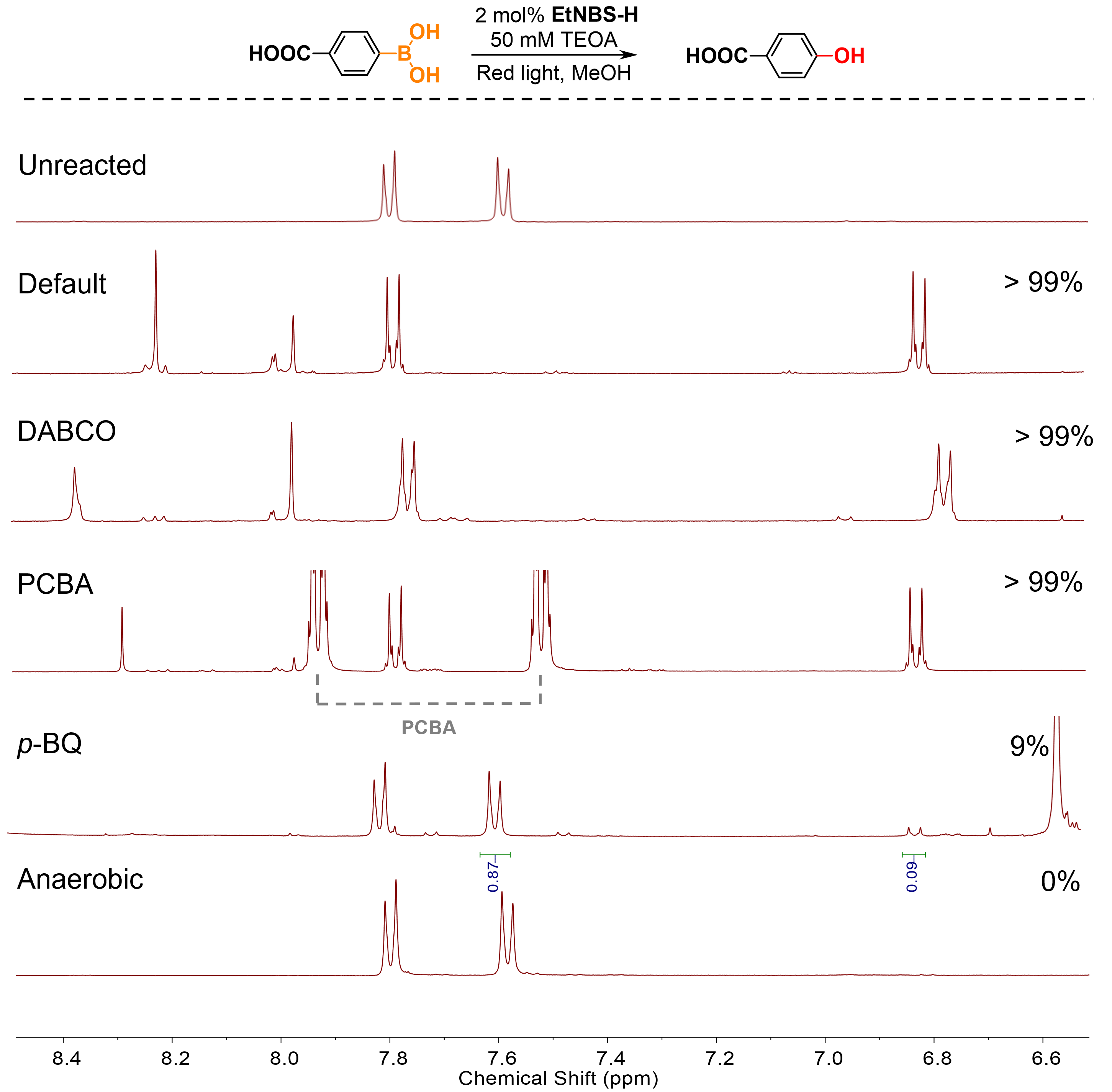


**Figure S35.** ^1^H NMR spectra of the products obtained from the photocatalytic oxidative hydroxylation of 4-carboxyphenylboronic in the presence of different quenchers in DMSO-*d_6_*. Reaction conditions: 4-carboxyphenylboronic (5 mM), TEOA (50 mM), quencher (25 mM), and **EtNBS-H** (2 mol%) in MeOH (2 mL), irradiated with red light for 2 h at room temperature under ambient atmosphere. Certain chemical shifts observed in the proton resonances of the products should be attributed to perturbations in the solvent environment upon the addition of DABCO.


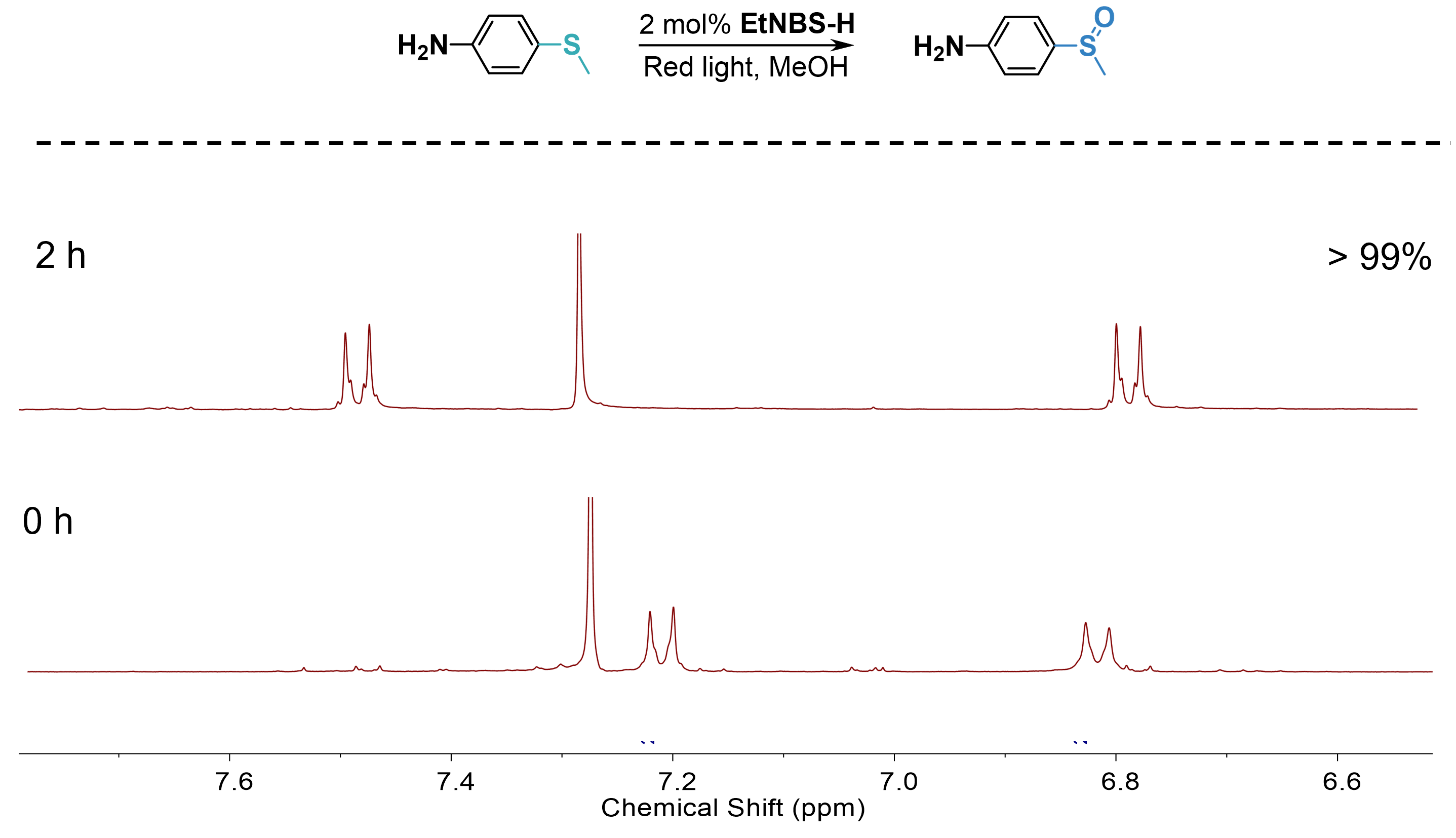


**Figure S36.** ^1^H NMR spectra of the products obtained from the photocatalytic oxidative reaction of 4-(methylmercapto) aniline in CDCl_3_.


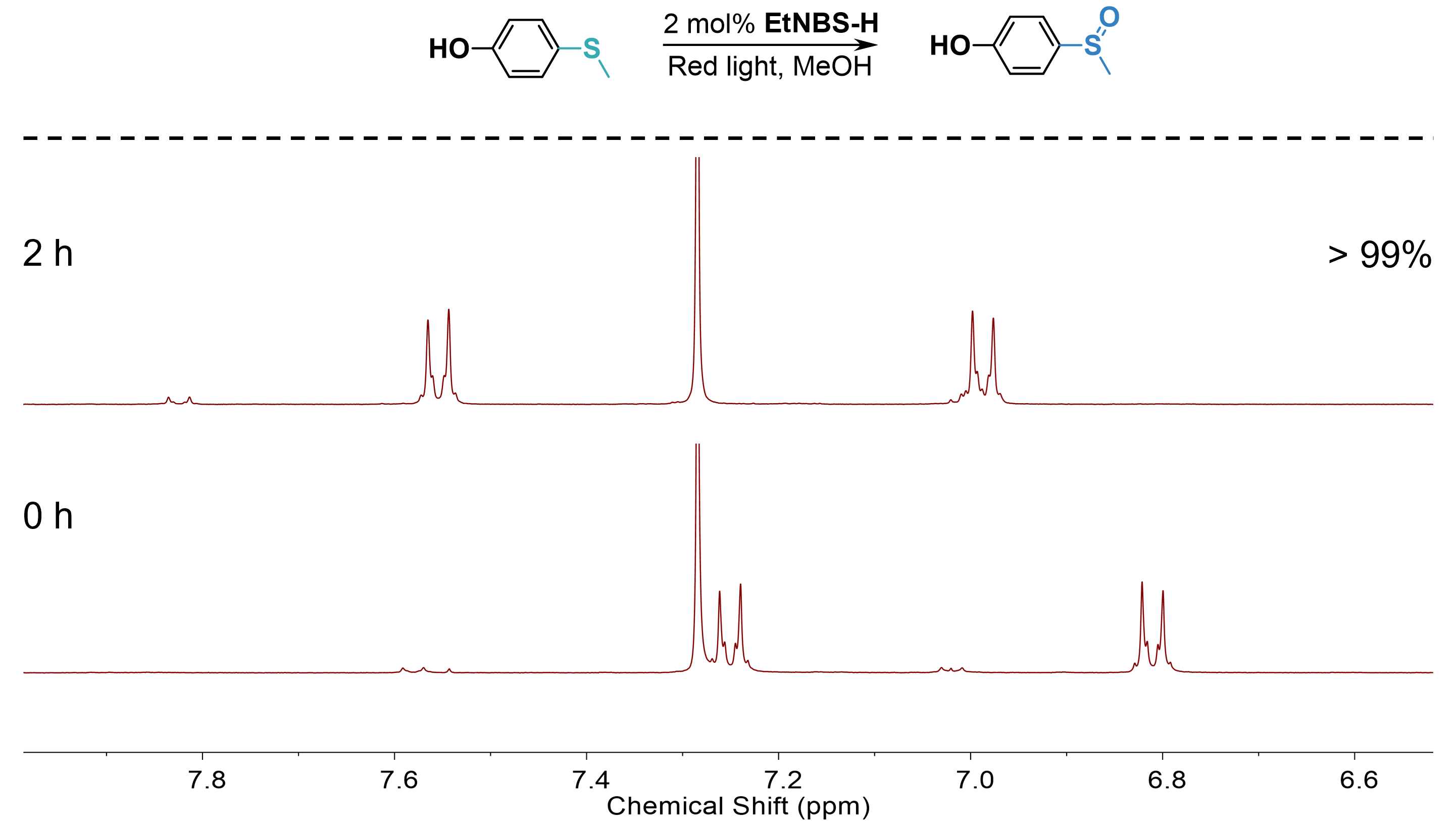


**Figure S37.** ^1^H NMR spectra of the products obtained from the photocatalytic oxidative reaction of 4-(methylsulphinyl)phenol in CDCl_3_.


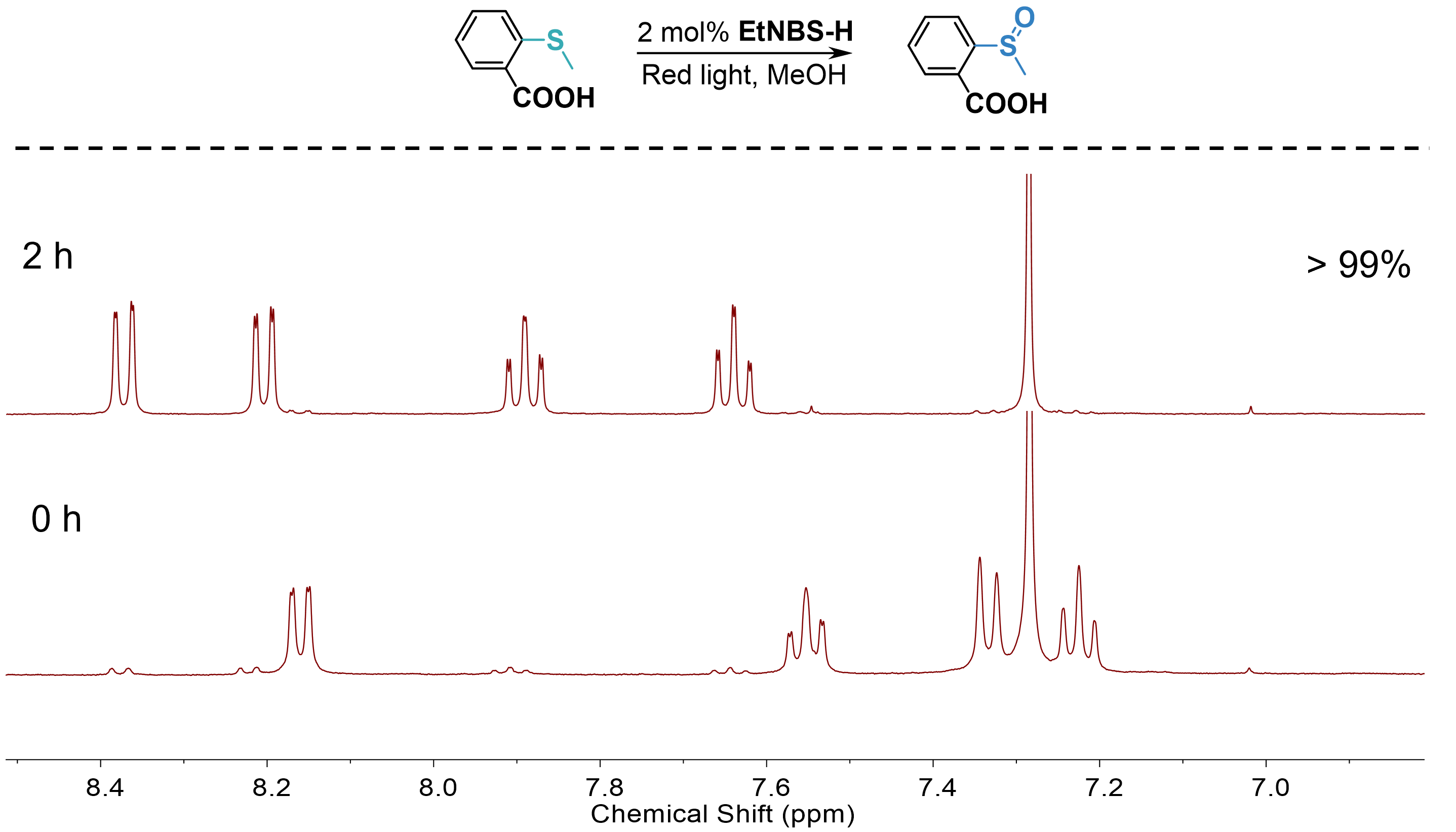


**Figure S38.** ^1^H NMR spectra of the products obtained from the photocatalytic oxidative reaction of 2-methylbenzothioic acid in CDCl_3_.


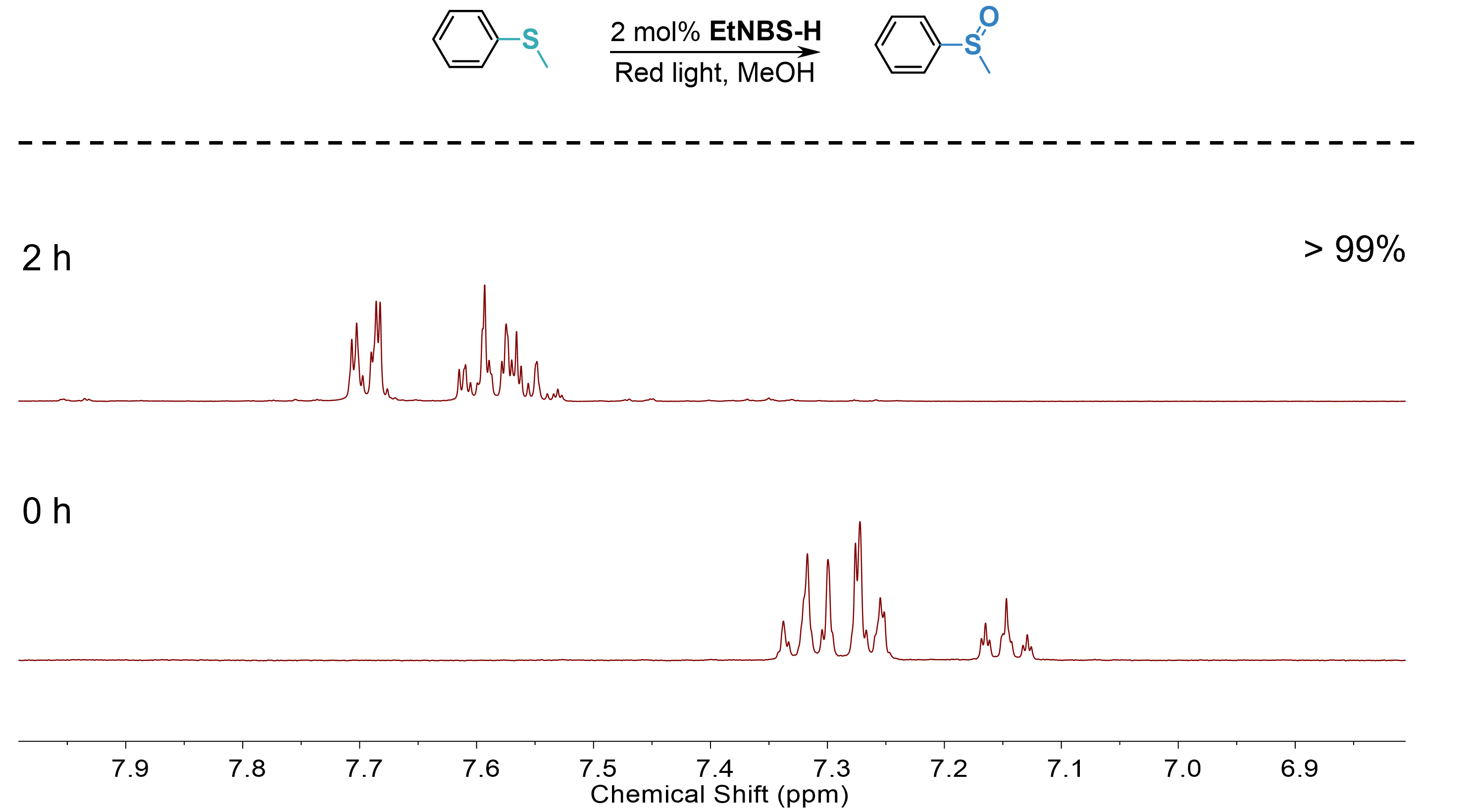


**Figure S39.** ^1^H NMR spectra of the products obtained from the photocatalytic oxidative reaction of thioanisole in DMSO-*d_6_*.


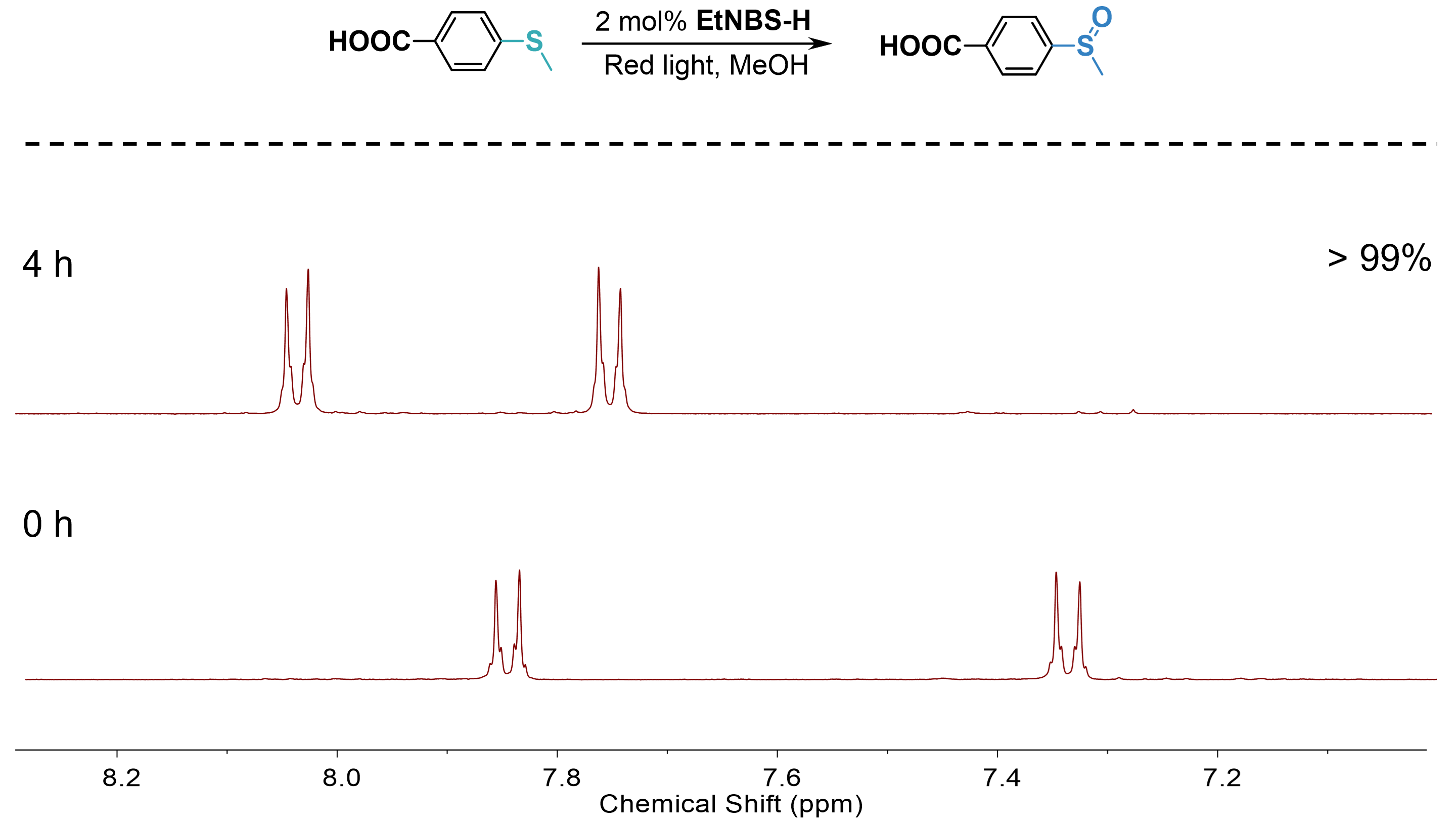


**Figure S40.** ^1^H NMR spectra of the products obtained from the photocatalytic oxidative reaction of 4-methylthiobenzoic acid in DMSO-*d_6_*.


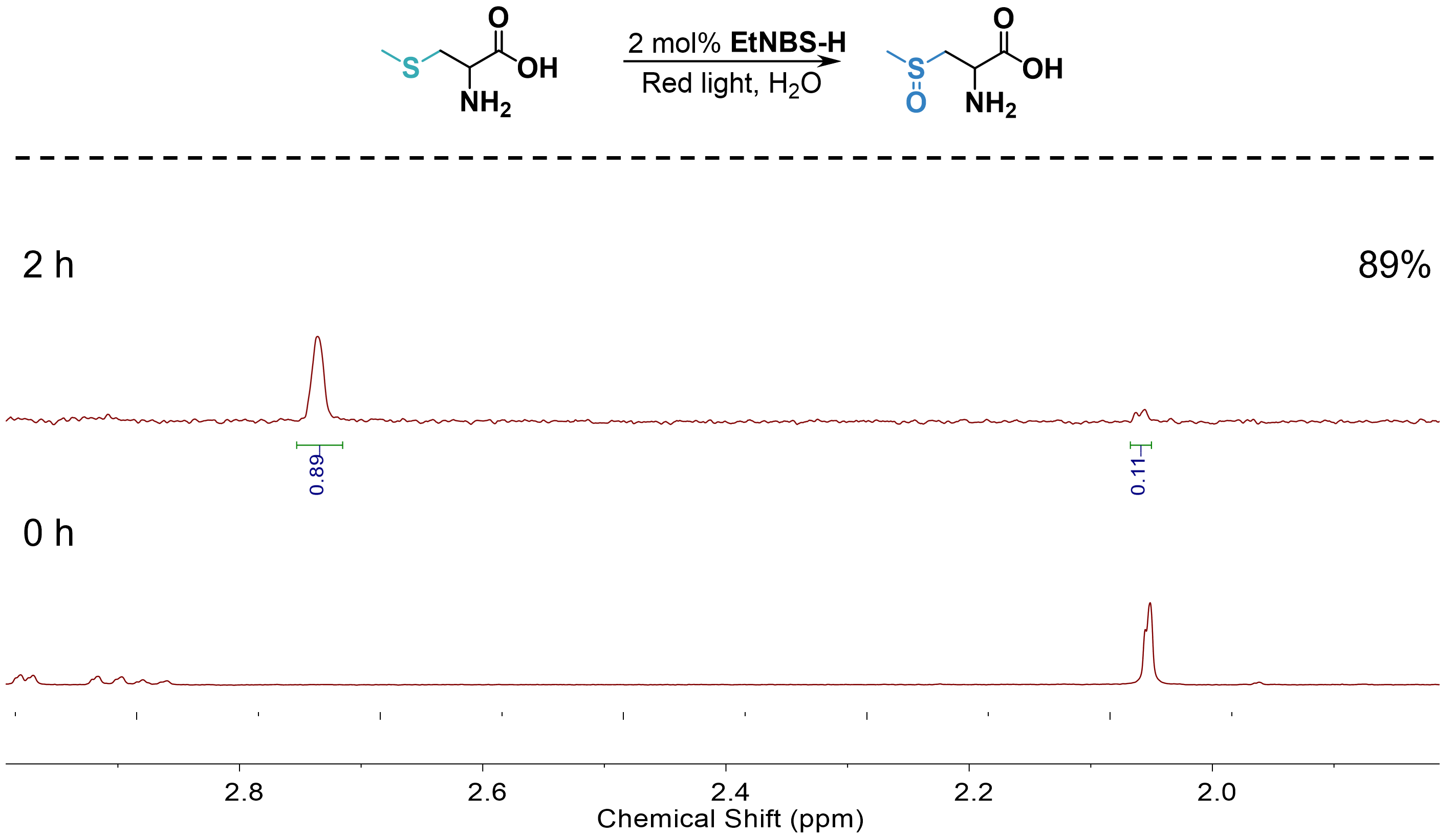


**Figure S41.** ^1^H NMR spectra of the products obtained from the photocatalytic oxidative reaction of S-methyl-L-cysteine in D_2_O.


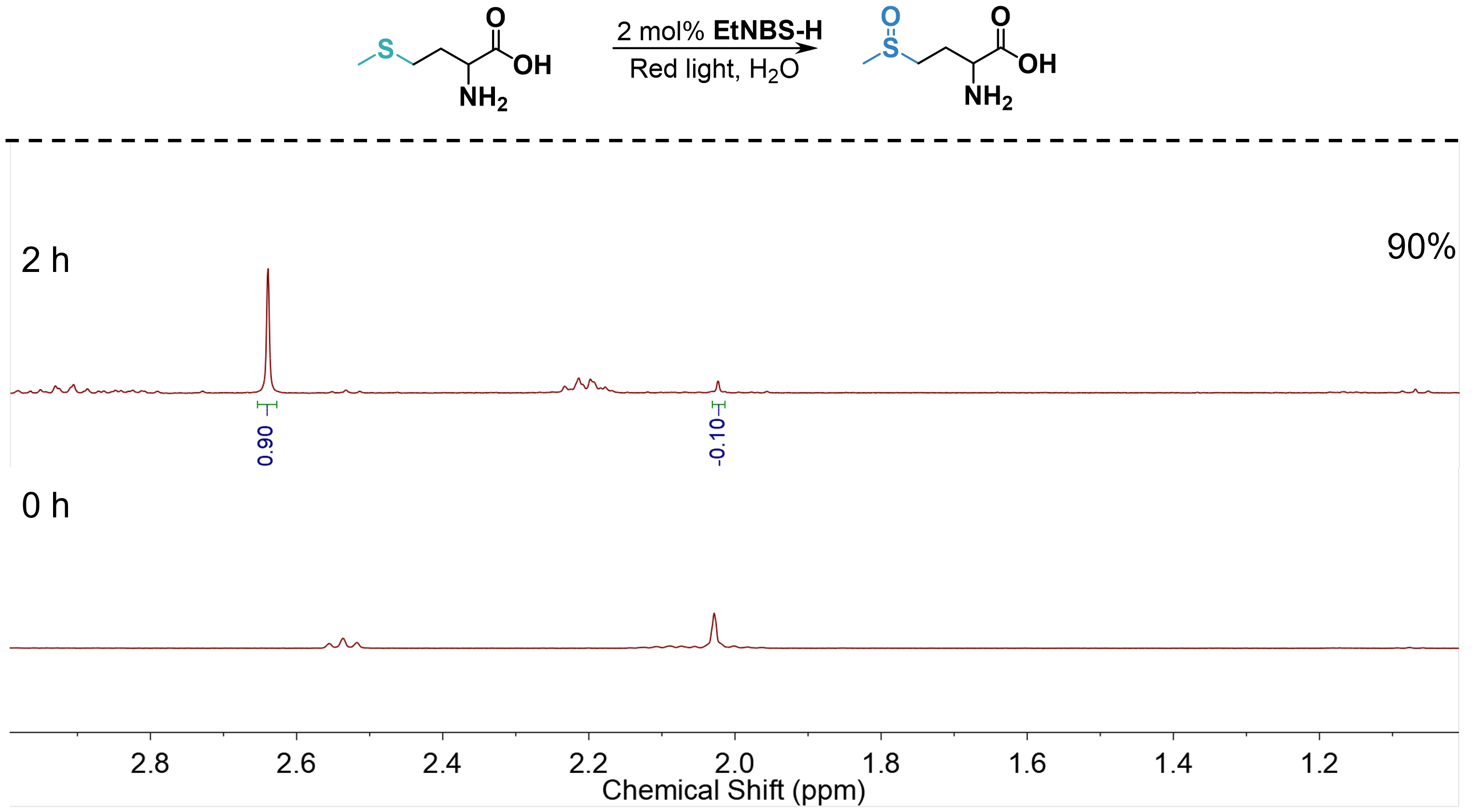


**Figure S42.** ^1^H NMR spectra of the products obtained from the photocatalytic oxidative reaction of DL-methionine in D_2_O.

**
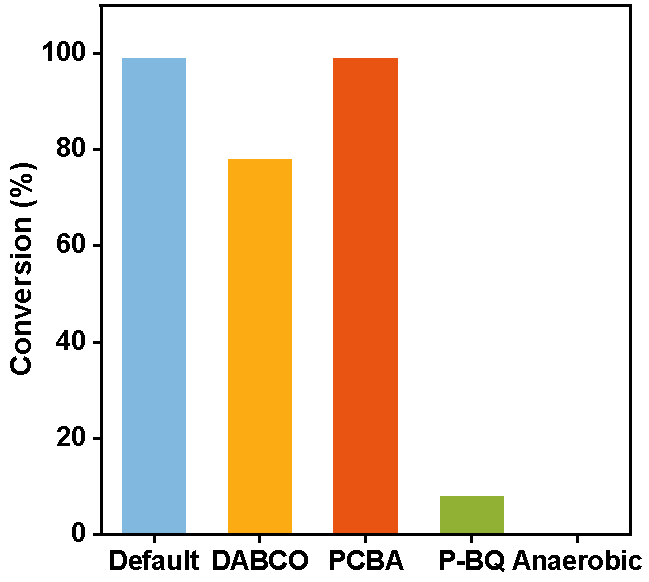
**

**Figure S43.** Photooxidation conversion of 4-methylthiobenzoic with or without quenchers. Default: without quenchers. DABCO: triethylenediamine (^1^O_2_). PCBA: 4-chlorobenzoic acid (•OH). *p*-BQ: p-benzoquinone (O_2_^−•^).


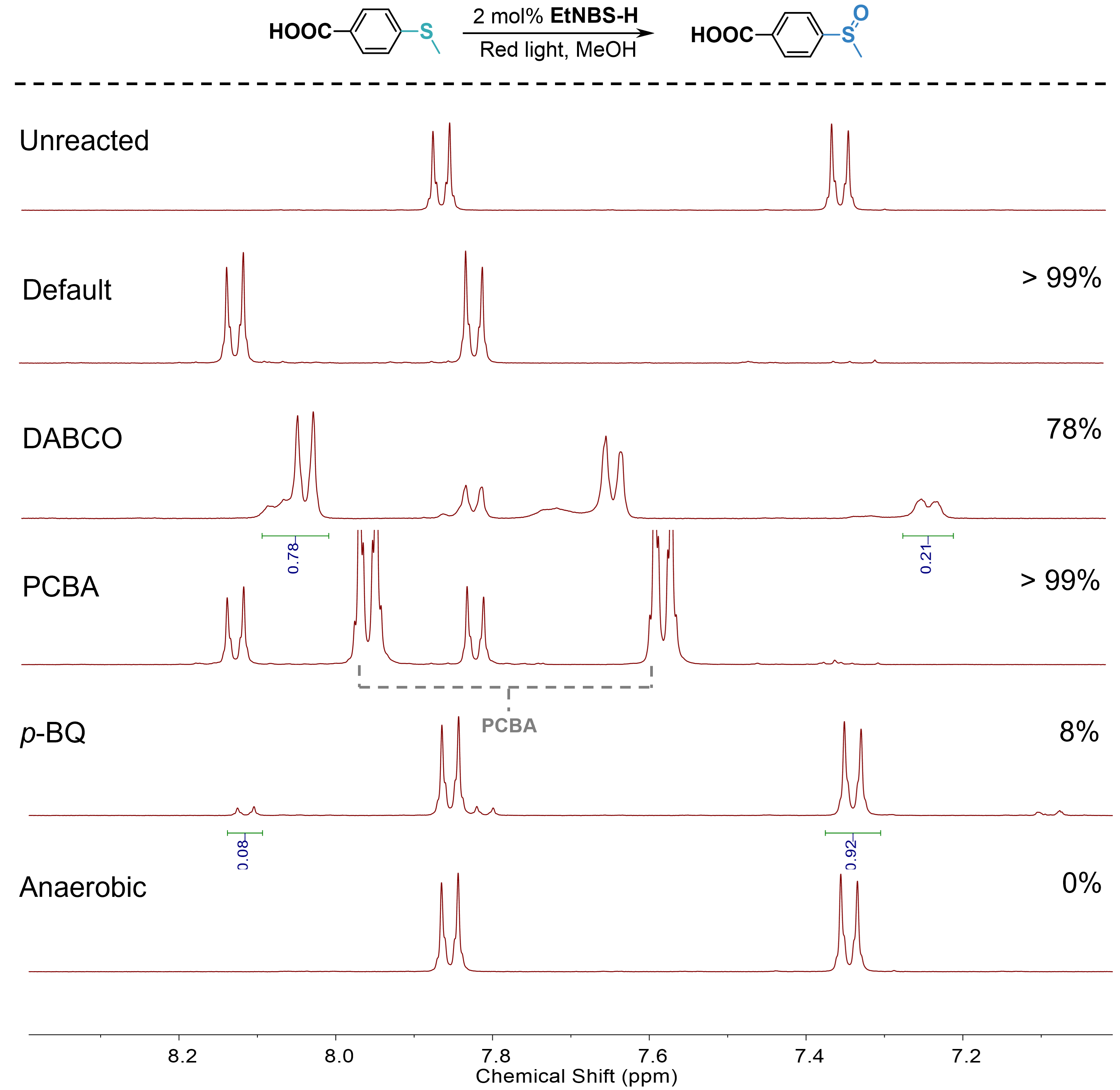


**Figure S44.** ^1^H NMR spectra of the products obtained from the photooxidation of thioethers in the presence of different quenchers in DMSO-*d_6_*. Reaction conditions: 4-methylthiobenzoic (5 mM), quencher (25 mM), and **EtNBS-H** (2 mol%) in MeOH (2 mL), irradiated with red light for 4 h at room temperature under ambient atmosphere. Certain chemical shifts observed in the proton resonances of the products should be attributed to perturbations in the solvent environment upon the addition of DABCO.


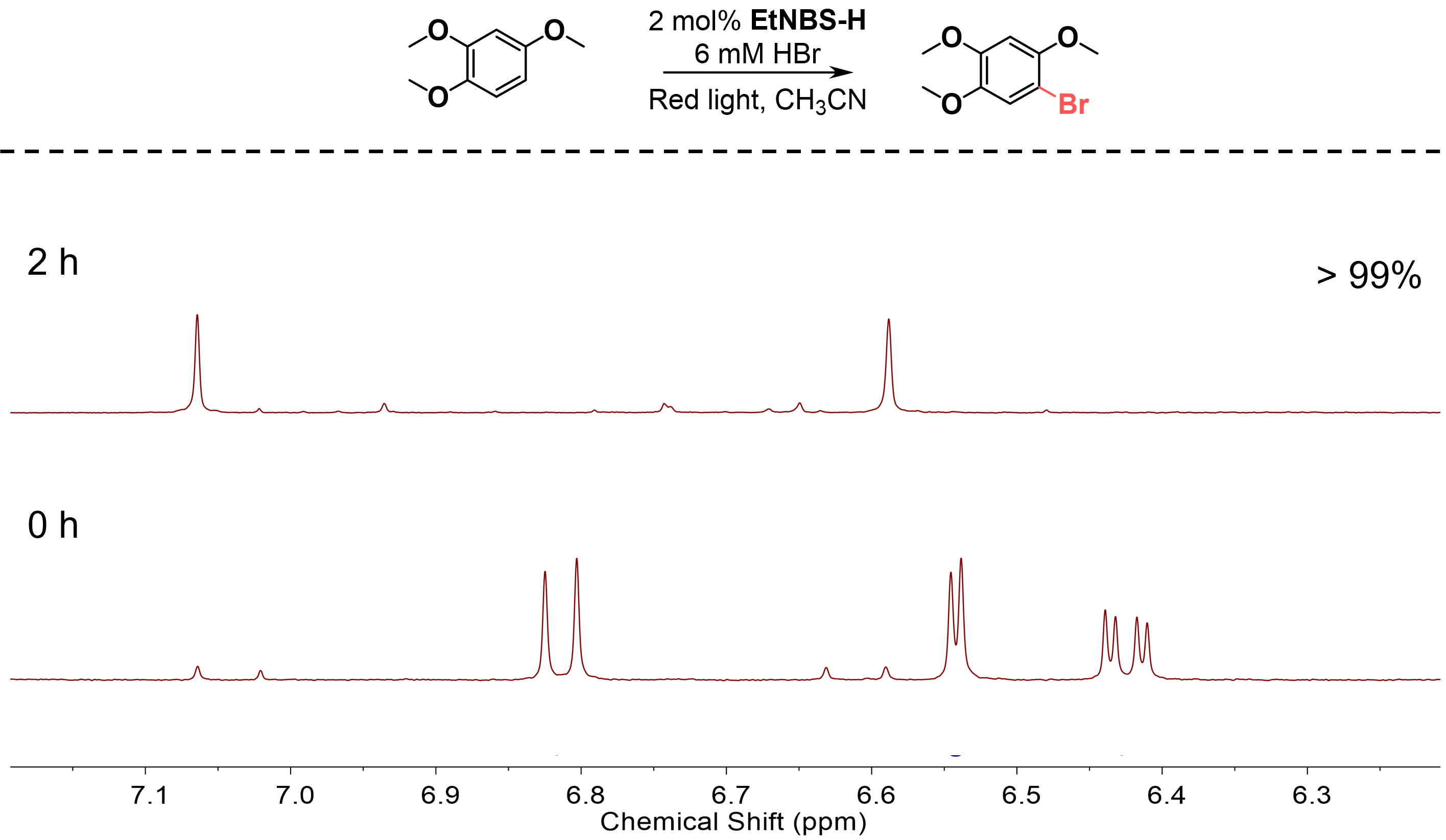


**Figure S45.** ^1^H NMR spectra of the products obtained from the photocatalytic bromination reaction of 1,3,4-trimethoxybenzene in CDCl_3_.


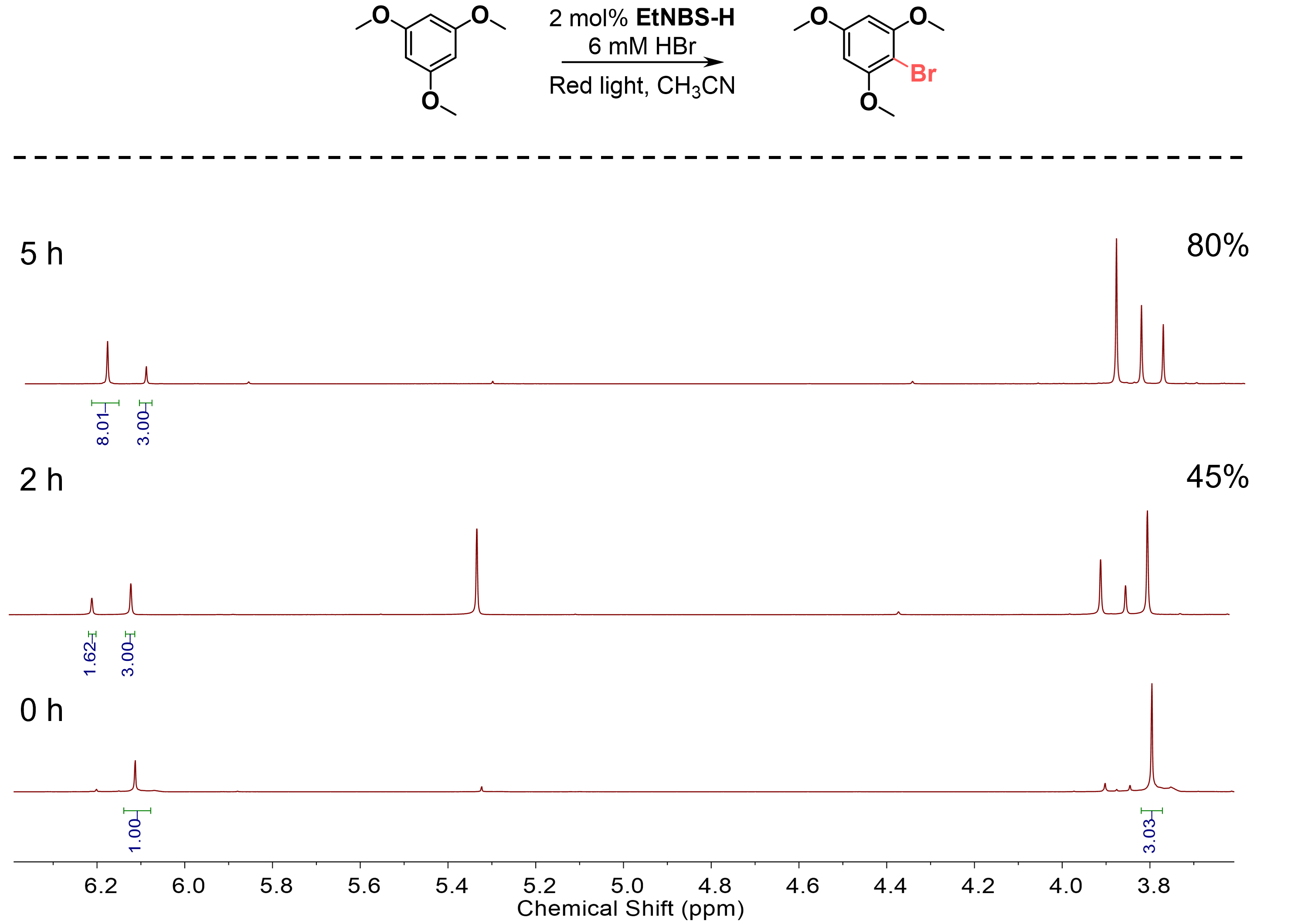


**Figure S46.** ^1^H NMR spectra of the products obtained from the photocatalytic bromination reaction of 1,3,5-trimethoxybenzene in CDCl_3_.


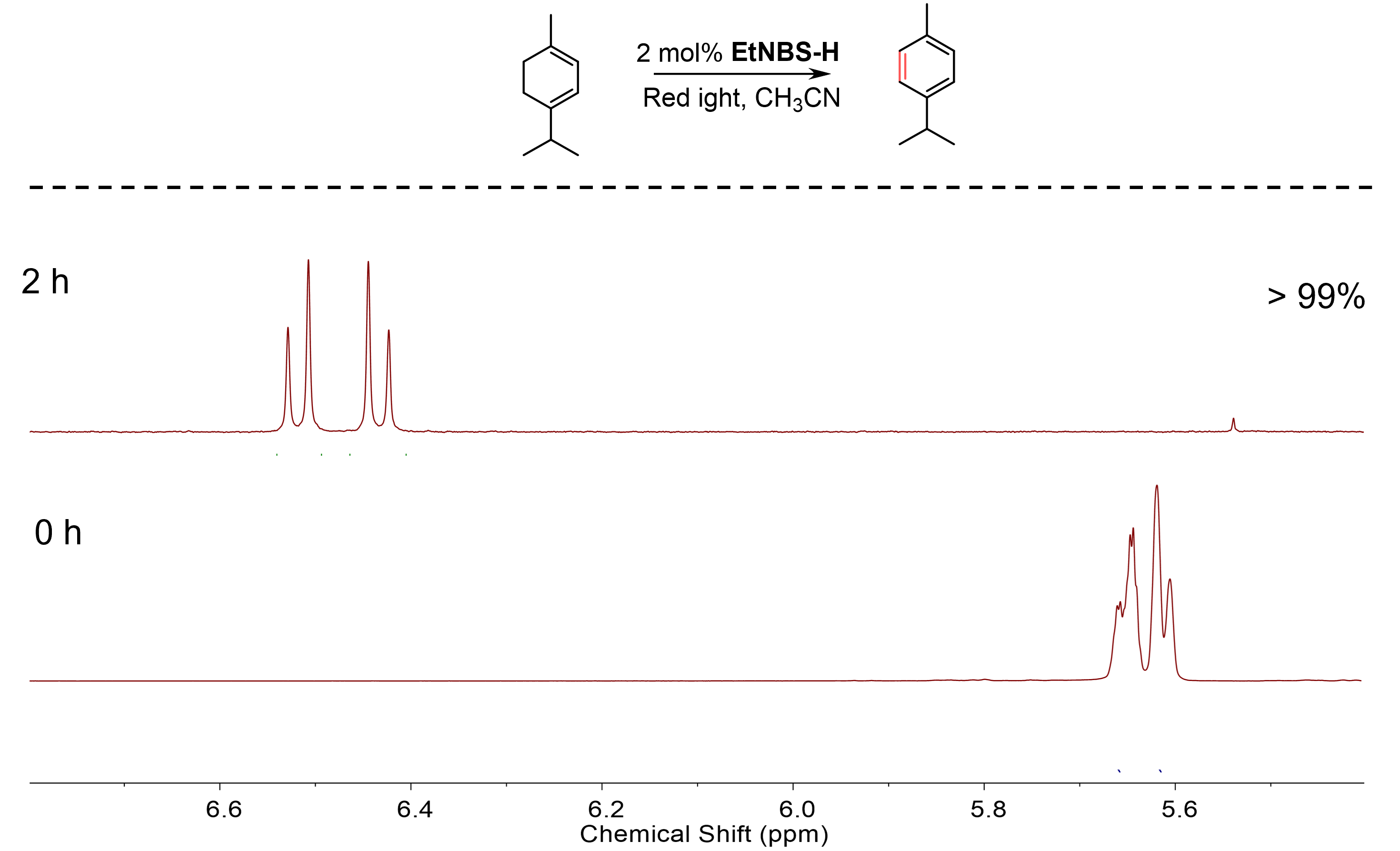


**Figure S47.** ^1^H NMR spectra of the products obtained from the photocatalytic oxidation reaction of α-terpinene in CDCl_3_.


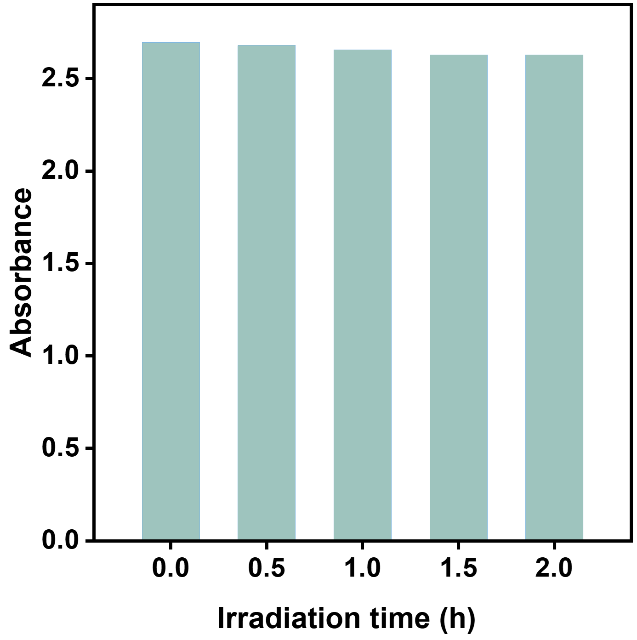


**Figure S48.** Absorbance decay of **EtNBS-H** (25 μM) in MeOH irradiation with red light for 2 h.


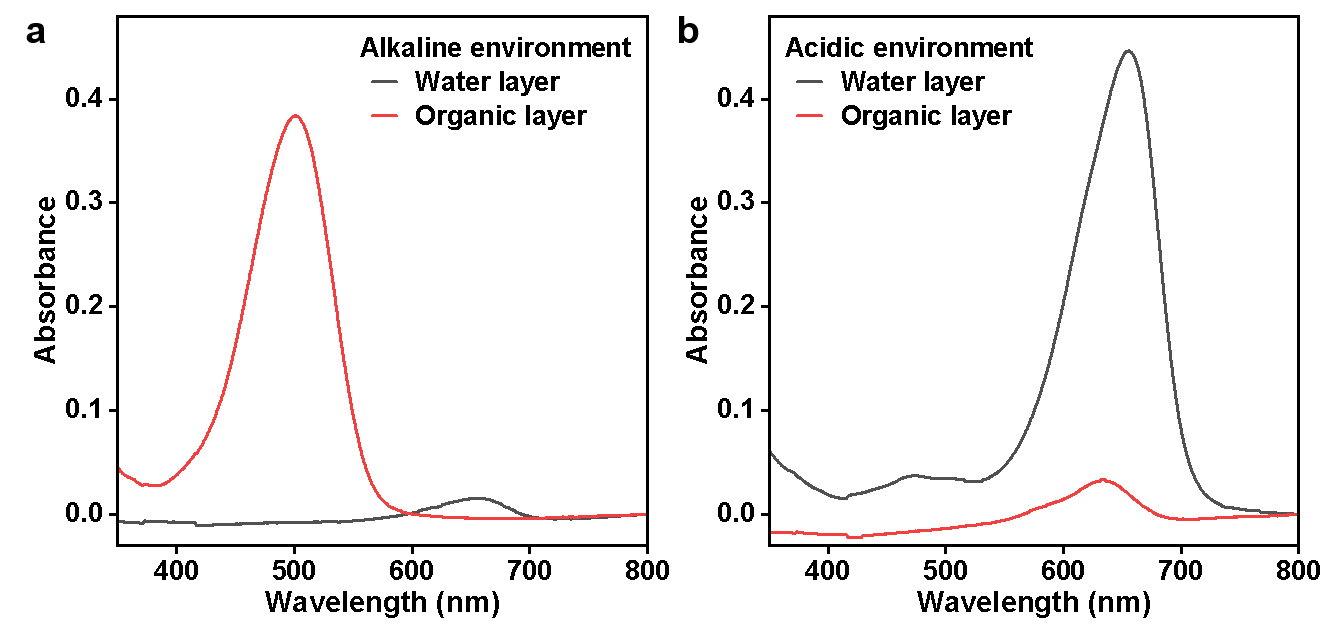


**Figure S49.** Absorbance for **EtNBS-H** in (a) alkaline and (b) acidic environments.


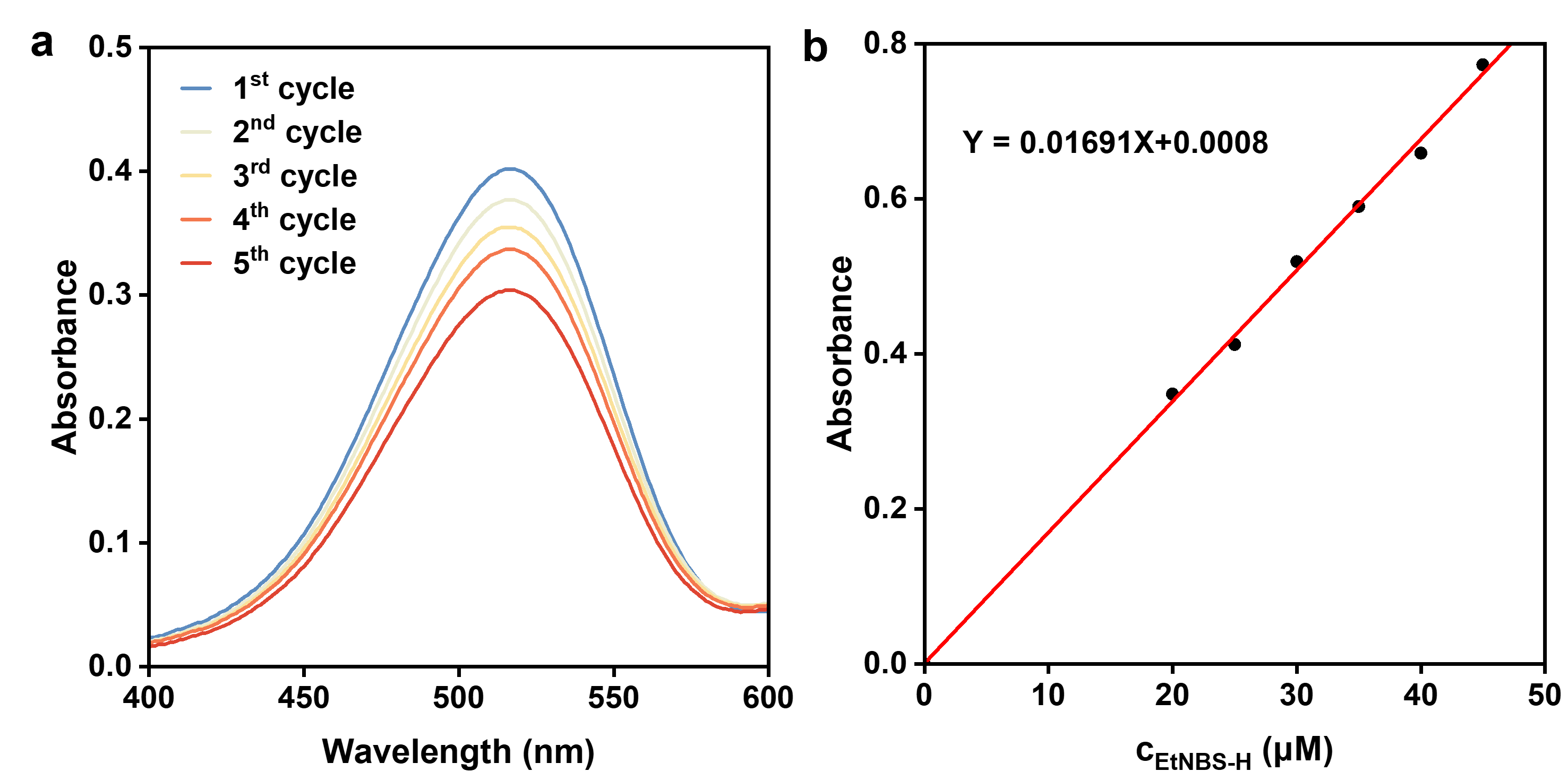


**Figure S50.** (a) Absorbance of **EtNBS-H** in alkaline environment with different number of cycles. (b) A linear relationship between the **EtNBS-H** concentrations and its absorbance intensity at 517 nm.

**Table S3.** **EtNBS-H** recovery with different number of cycles.

| Number of cycles | Absorbance | Calculate the concentration (μM) | Theoretical concentration (μM) | Recovery (%) |
| --- | --- | --- | --- | --- |
| 1^st^ | 0.402 | 23.74 | 25 | 94.96 |
| 2^nd^ | 0.377 | 22.26 | 25 | 89.04 |
| 3^rd^ | 0.355 | 20.96 | 25 | 83.83 |
| 4^th^ | 0.337 | 19.90 | 25 | 79.57 |
| 5^th^ | 0.304 | 17.94 | 25 | 71.76 |


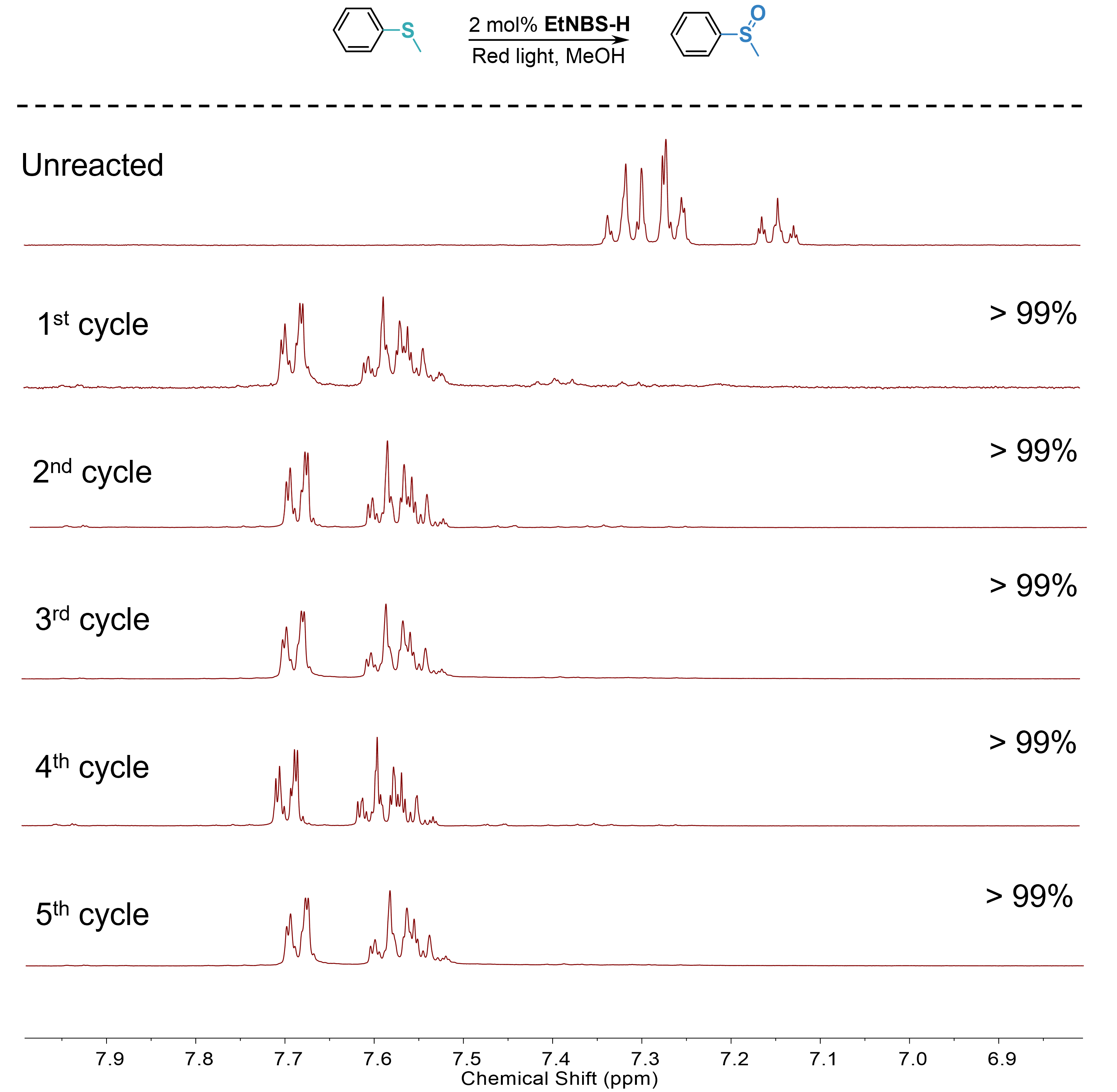


**Figure S51.** ^1^H NMR spectra of the products obtained from the photocatalytic oxidative reaction of thioanisole for 5 cycles in DMSO-*d_6_*.


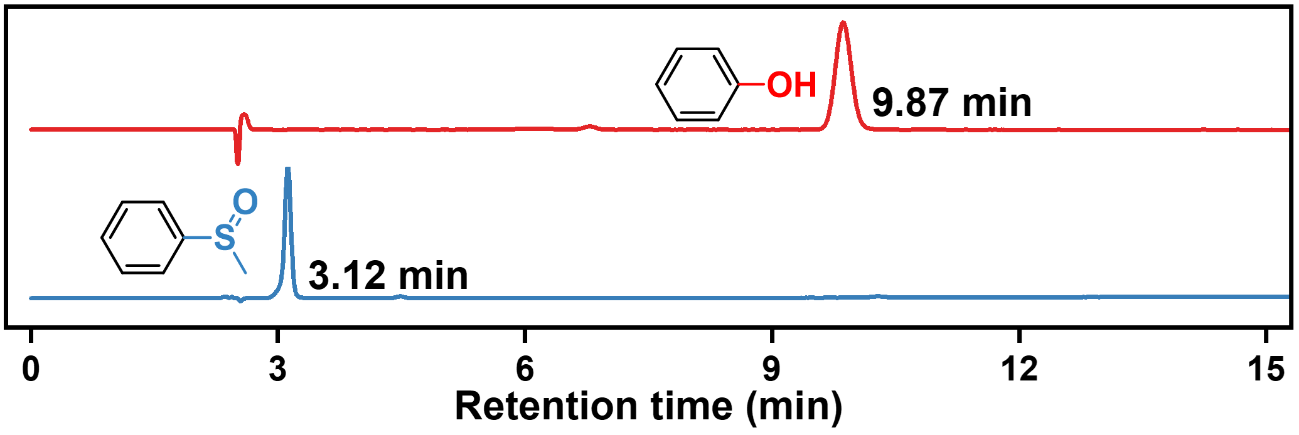


**Figure S52.** HPLC spectra of the standard product of phenol (upper, detection wavelength: 254 nm, methanol:water = 30 : 70, v/v) and (methylsulfinyl)benzene (lower, detection wavelength: 254 nm, methanol:water = 60 : 40, v/v).


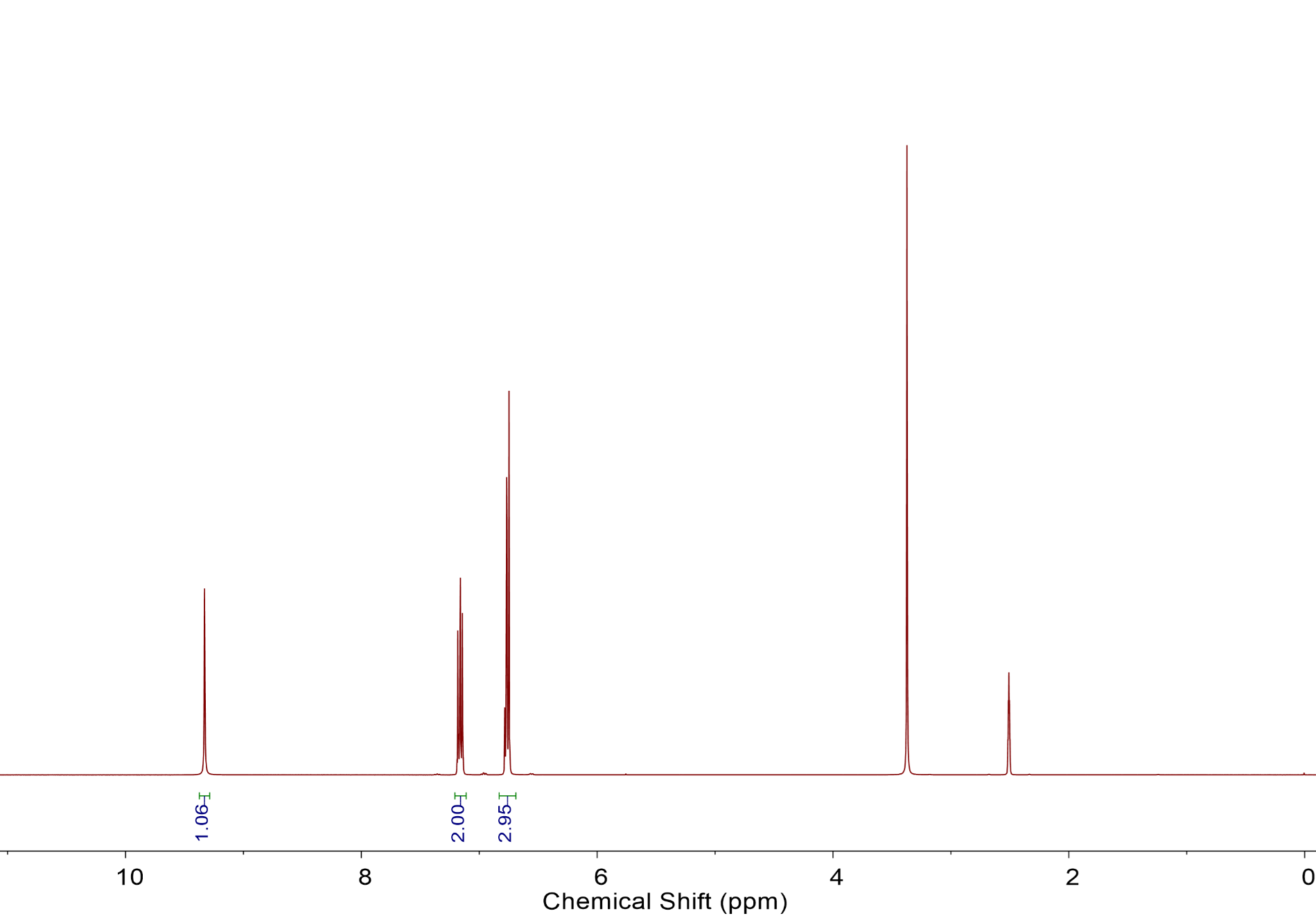


**Figure S53.** ^1^H NMR spectrum of the products obtained by extraction after photocatalysis with phenylboronic acid in DMSO-*d_6_*.


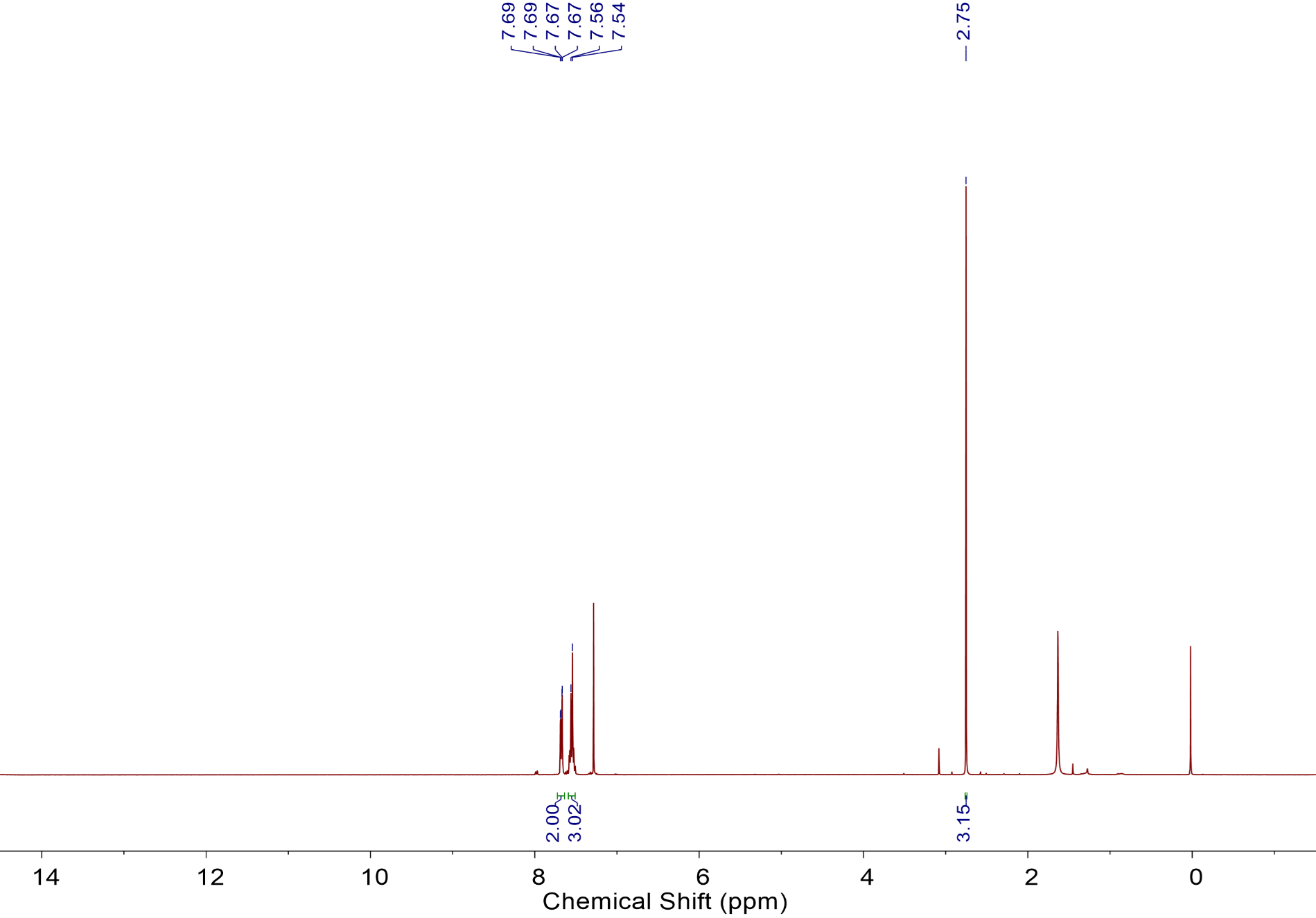


**Figure S54.** ^1^H NMR spectrum of the products obtained by extraction after photocatalysis with thioanisole in CDCl_3_.


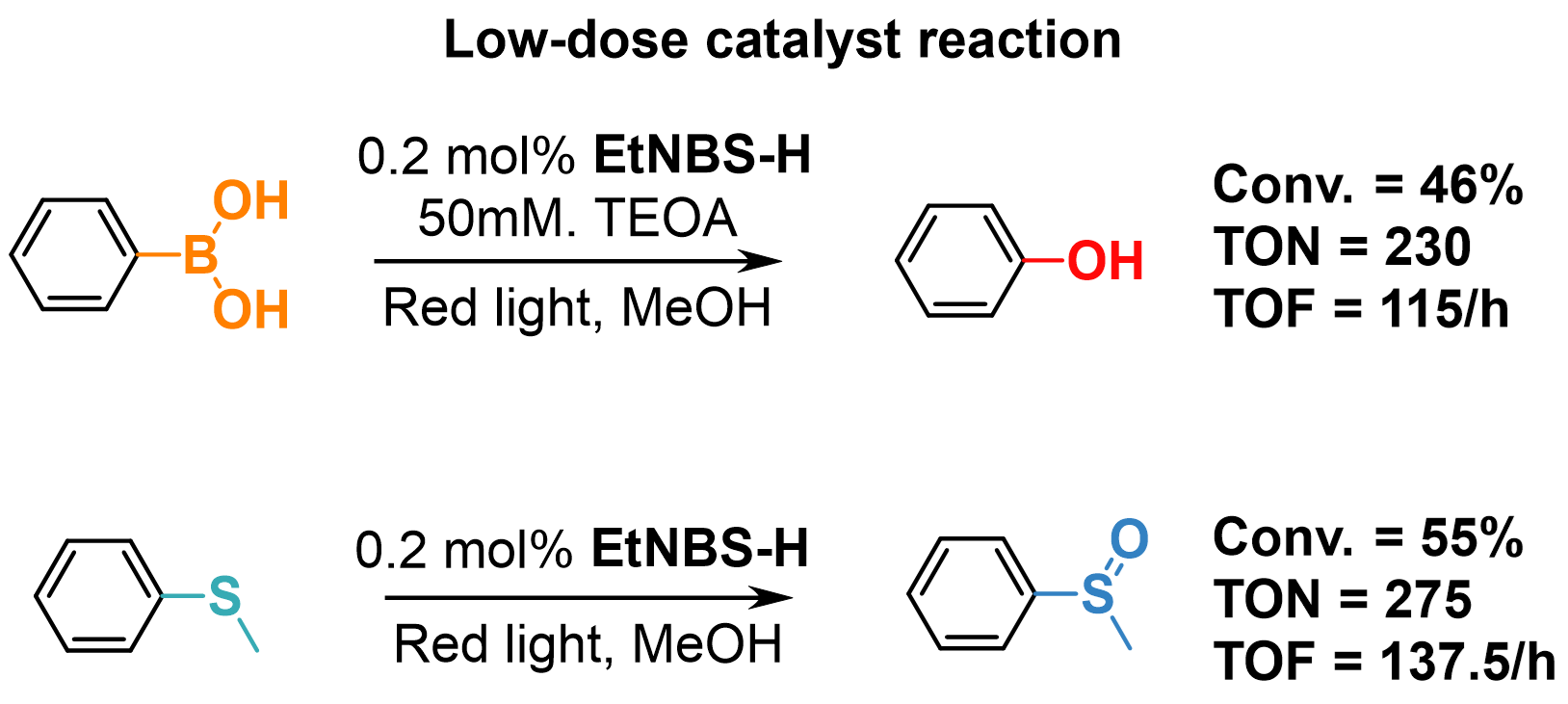


**Figure S55.** TON and TOF calculations for reactions.

**Table S4.** Ecological scale calculations of the photocatalytic reaction of phenylboronic acid.

| EcoScale = 100 - Sum of individual penalties Score on EcoScale: > 75, Excellent; > 50, Acceptable; < 50, Inadequate | | |
| --- | --- | --- |
| A) Calculation of penalty points: | | |
| Parameters | | Penalty points |
| 1. Yield | (100-100*yield)/2 = (100-96)/2 = 2 | 2 |
| 2. Price of reaction components (to obtain 10 mmol of end product) | (a) Substrate X = 1.242 g < $1 | 0 |
|  | (b) PC = 0.771 mg < $ 5 |  |
|  | Total price < $6 |  |
|  | Inexpensive (< $10) |  |
| 3. Safety | Triethanolamine (toxic、highly flammable) | 10 |
| 4. Technical setup | Inconventional activation technique (Photochemical reaction) | 2 |
| 5. Temperature and time | Room temperature, < 24 h | 1 |
| 6. Workup and purification | Liquid-liquid extraction | 3 |
| Total Penalty Points |  | 28 |
| B) EcoScale calculation: | | |
| EcoScale | = 100 - Total Penalty Points | 82 |

**Table S5.** Ecological scale calculations of the photocatalytic reaction of thioanisole.

| EcoScale = 100 - Sum of individual penalties Score on EcoScale: > 75, Excellent; >50, Acceptable; < 50, Inadequate | | |
| --- | --- | --- |
| A) Calculation of penalty points: | | |
| Parameters | | Penalty points |
| 1. Yield | (100-100*yield)/2 = (100-97)/2 = 3.4 | 1.5 |
| 2. Price of reaction components (to obtain 10 mmol of end product) | (a) Substrate X = 1.280 g < $1 | 0 |
|  | (b) PC = 0.763 mg < $ 5 |  |
|  | Total price < $6 |  |
|  | Inexpensive (< $10) |  |
| 3. Safety |  | 0 |
| 4. Technical setup | Inconventional activation technique (Photochemical reaction) | 2 |
| 5. Temperature and time | Room temperature, < 24 h | 1 |
| 6. Workup and purification | Liquid-liquid extraction | 3 |
| Total Penalty Points |  | 17.5 |
| B) EcoScale calculation: | | |
| EcoScale | = 100 - Total Penalty Points | 92.5 |
